# *Teg58*, a small regulatory RNA, is involved in regulating arginine biosynthesis and biofilm formation in *Staphylococcus aureus*

**DOI:** 10.1038/s41598-022-18815-3

**Published:** 2022-09-02

**Authors:** Adhar C. Manna, Stefano Leo, Sergey Girel, Víctor González-Ruiz, Serge Rudaz, Patrice Francois, Ambrose L. Cheung

**Affiliations:** 1 Department of Microbiology & Immunology, Geisel School of Medicine at Dartmouth, Hanover, NH 03755 USA; 2 Genomic Research Laboratory, Service of Infectious Diseases, Geneva University Hospitals and University Medical Center, Rue Michel-Servet 1, 1211 Geneva 4, Switzerland; 3 Institute of Pharmaceutical Sciences of Western Switzerland (ISPSO), School of Pharmaceutical Sciences, University of Geneva, Geneva, Switzerland; 4 University Medical Center, Rue Michel-Servet 1, 1211 Geneva 4, Switzerland

**Keywords:** Biofilms, Cellular microbiology

## Abstract

*Staphylococcus aureus* adapts to different environments by sensing and responding to diverse environmental cues. The responses are coordinately regulated by regulatory proteins, and small regulatory RNAs at the transcriptional and translational levels. Here, we characterized *teg58*, a SarA repressed sRNA, using ChIP-Seq and RNA-Seq analysis of a *sarA* mutant. Phenotypic and genetic analyses indicated that inactivation of *teg58* led to reduced biofilm formation in a process that is independent of SarA, *agr*, PIA, and PSMs. RNA-Seq analysis of *teg58* mutant revealed up-regulation of arginine biosynthesis genes (i.e., *argGH*) as well as the ability of the mutant to grow in a chemical defined medium (CDM) lacking l-arginine. Exogenous l-arginine or endogenous induction of *argGH* led to decreased biofilm formation in parental strains. Further analysis in vitro and in vivo demonstrated that the specific interaction between *teg58* and the *argGH* occurred at the post-transcriptional level to repress arginine synthesis. Biochemical and genetic analyses of various arginine catabolic pathway genes demonstrated that the catabolic pathway did not play a significant role in reduced biofilm formation in the *teg58* mutant. Overall, results suggest that *teg58* is a regulatory sRNA that plays an important role in modulating arginine biosynthesis and biofilm formation in *S. aureus*.

## Introduction

*Staphylococcus aureus* is the causative agent of numerous infections ranging from minor skin conditions to fatal systemic syndromes^1^. Its ability to cause extensive infections relies on an extensive arsenal of virulence and metabolic factors to facilitate each of the pathogenic steps including adherence, growth, mature biofilm formation and dispersal^1–3^. Expression of these factors are mediated by two-component regulatory systems, transcriptional regulators, and riboswitches/small regulatory RNAs (sRNAs)^4–6^.

sRNAs have emerged in the past decade as important players in the post-transcriptional regulation of target genes, enabling rapid environmental adaptation via posttranscriptional processing (e.g., mRNA stability and translation) as opposed to induced transcription via transcription factors^7,8^ which is a slower process. In contrast to Gram-negative bacteria, the RNA chaperone Hfq^9^ is not required for sRNA-dependent regulations in *S. aureus*^10–12^.

There are more than 300 potential sRNAs in *S. aureus* genomes, identified mainly by RNA-Seq^13–15^. The best described sRNA in *S. aureus* is RNAIII, a dual-function sRNA which, at 514-nt long, codes for delta toxin as well as functions as a sRNA that binds target mRNAs to promote mRNA degradation (e.g., for *spa* and *coa, sbi*, *ltaS*, *lytM*, *rot* and other mRNAs) or improve translation (e.g., *hla*, *map*, *mgrA*)^7,16,17^. RsaE has been shown to down-regulate several genes including direct interaction with arginase *rocF* mRNA, which is associated with arginine catabolism^18^. Not surprisingly, additional sRNAs were found (e.g., Teg27, *sprC,* Teg10, RasI, RsaD, *sprX2,* Teg41) to regulate assorted target mRNAs^19–24^ but their roles in biofilm formation have not been defined except for RsaI^22^.

Infections attributable to *S. aureus* biofilms are difficult to treat due to reduced efficacy of host innate immune defense, barrier to antibiotic penetration, and slower cellular growth vs. their planktonic counterparts^25–27^. Biofilm can be divided into different developmental stages including attachment, aggregation, maturation, and detachment^28^. While past studies have focused on adhesins (e.g., Aap, Fnb, SasG, Spa, ClfB, to name a few)^25–27^, biofilm matrix structures [PIA encoded by the *icaADBC* genes and regulated by IcaR, SarA, and other regulators)]^28,29^, biofilm maturation (secreted proteins and eDNA release)^30^ and dispersal [protease, nuclease, PSM (surfactant) and dispersin B]^25–27^, the role of metabolic factors on biofilm formation in *S. aureus* has not been well studied.

**Figure 1:**
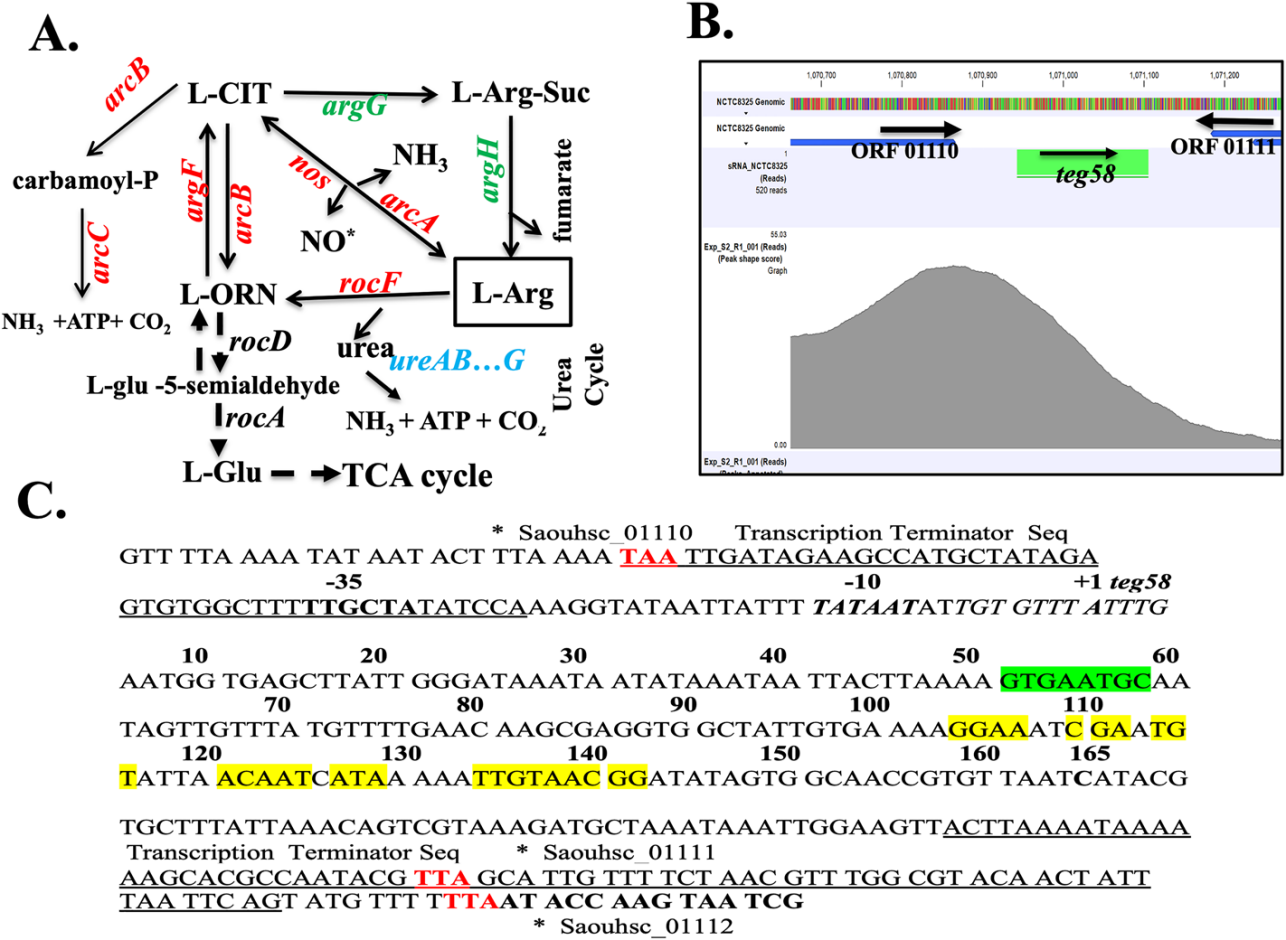
Characterization of sRNA *teg58*. (**A**) Putative arginine synthesis and catabolic pathways in *S. aureus*. Adopted from Ibberson &Whiteley^39^. CIT, citrulline; Arg-Suc, arginosuccinate; ORN, ornithine. (**B**) Normalized occupancy profile for SarA peak located between sRNA *teg58* (srn_2460) and Saouhsc_01110 open reading frame as analyzed by using CLC Genomics Workbench 11. Arrows indicated the direction of the genes or sRNA. (**C**) Nucleotide sequence of the *teg58* region was shown and marked, indicating putative promoter region, start site as derived from primer extension data, transcriptional terminator and translational stop sequences (red) of the up- and down-stream genes. The ChIP-Seq motif and published SarA binding operator sequences^40^ is in the same region as marked by red underline. Green and yellow marking indicate the potential interaction sites of *argG* and *argH* mRNAs/genes, respectively, with *teg58* as determined by Freiburg IntaRNA Tools^41^.

Arginine is one of the important amino acids, which can be utilized as an energy source and building blocks in protein synthesis^31^. Arginine was found to inhibit biofilm formation in several bacteria, possibly by neutralizing acidic pH, and/or inhibiting co-aggregation of cells^32–34^. In *S. aureus*, the primary pathway for synthesis of l-arginine from glutamate or proline via citrulline metabolism entails two genes, *argG* and *argH*, transcribed monocistronically. In contrast, catabolism of arginine entails several pathways (Fig. 1A): the deiminase pathway encompassing mostly ArcA and ArcB whereby arginine can be converted to l-citrulline^35–39^ and the arginase pathway where RocF (arginase) catalyzes the conversion of l-arginine to urea and l-ornithine, which can be converted to l-citrulline by ArgF. l-citrulline can be catabolized back to l-ornithine by ArcB. l-ornithine, which eventually generates l-glutamate by RocD and RocA, feeds to the TCA cycle^39^. Although, the predominant pathway for l-arginine catabolism in *S. aureus* is not well understood, several genes of the catabolism pathway are regulated by the carbon catabolite repressor (CcpA)^36,37^.

In this study, we characterized the sRNA *teg58* by analyzing ChIP-Seq and RNA-Seq results of a *sarA* mutant^40^. Genetic analysis revealed *teg58* to be repressed by SarA. Biofilm formation is significantly decreased in the *teg58* mutant in a manner independent of *agr*, SarA as well as matrix-related components PIA. RNA-Seq analysis of the *teg58* mutant revealed increased *argGH* transcription vs. the parent. Northern blots, growth analysis in chemically defined medium^42^ (CDM) with and without arginine, and mutagenesis studies involving *teg58* and *argGH* mRNA as well as half-life studies of *argGH* mRNA demonstrated that the sRNA-mRNA interaction is specific that affected production of arginine. Genetic and phenotypic analysis of genes in the arginine catabolic pathway suggested that these genes are not significantly involved in *teg58*-mediated biofilm formation in *S. aureus*. Collectively, our data provided the molecular linkage of sRNA *teg58* to repression of *argGH,* leading to biofilm formation in *S. aureus*.

## Results

### Characterization of sRNA *teg58* (srn_2460) in *S. aureus*

To characterize sRNA directly regulated by SarA, we have performed ChIP-Seq analysis with anti-SarA and anti-Myc antibodies using cell extracts of a *sarA* mutant of HG003 harboring pSK236^43^::*sarA-myc*, followed by pulldown with Protein A-Sepharose to yield SarA-DNA complexes^40^. One of the pull-down fragments identified was a DNA fragment upstream of *teg58* with a peak shape score of 46.0 and the p-value of 0.00 (Fig. 1B). In addition, RNA-Seq of isogenic *sarA* mutant strains revealed increase in *teg58* transcript in *sarA* mutant vs. respective parent (2.41-fold)^40^. Primer extension and 3´ RACE experiments were performed to map 5′- and 3′-ends of *teg58* with total RNAs isolated from isogenic *sarA* strains (Fig. S1B for 3′-RACE), showing the sRNA to be 165-nt long (Fig. 1C). Further analysis revealed no well-defined ribosome binding site and coding region within the *teg58* transcript, consistent with a non-coding sRNA (Fig. 1C). *Teg58*, located in the intergenic region at the 3’ end of a gene cluster encoding putative virulence factors (Fig. S1A), is conserved in multiple *S. aureus* genomes.

### SarA repressed sRNA *teg58* transcription

**Figure 2:**
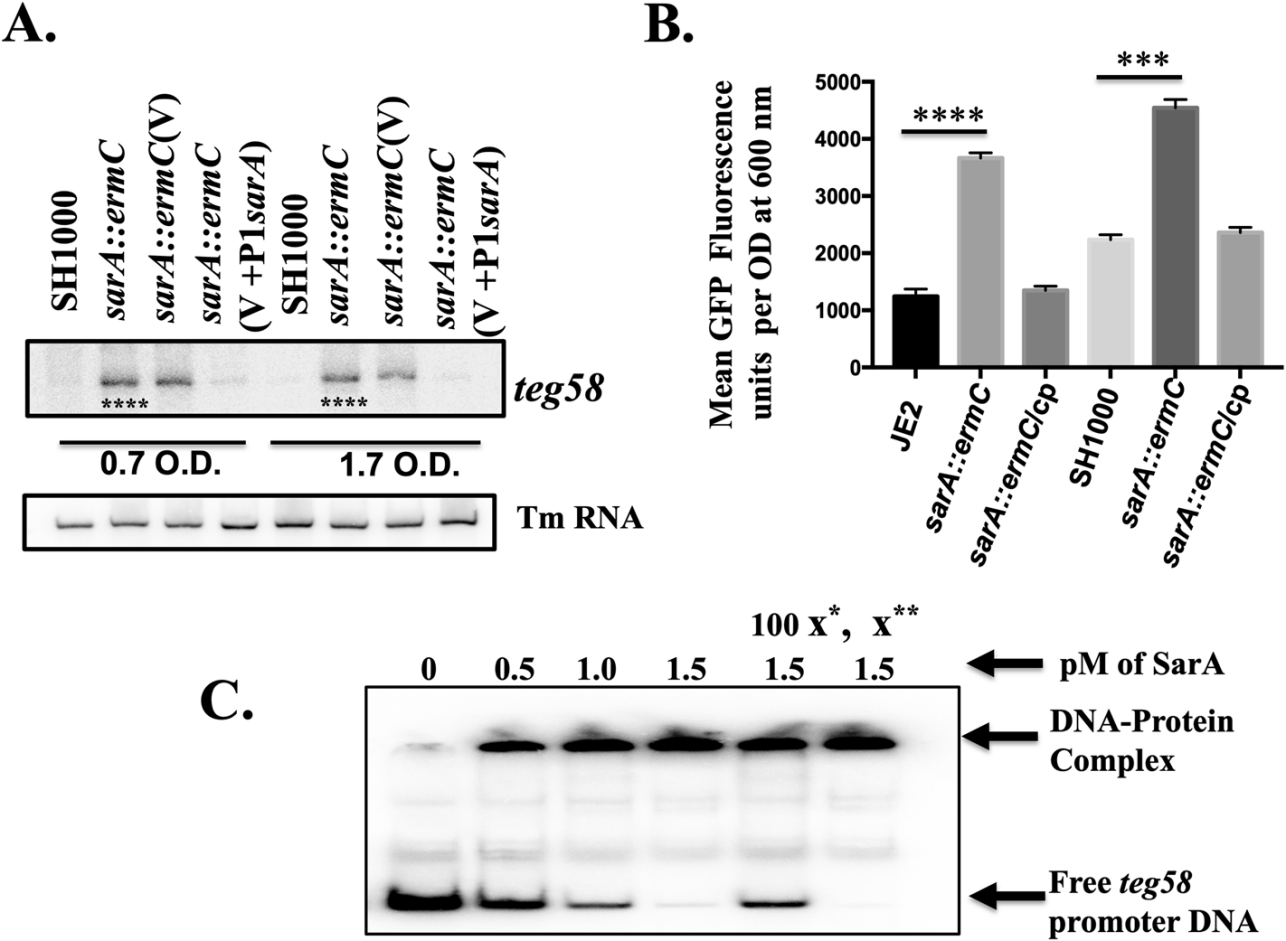
SarA repressed *teg58*. (**A**) Northern blot analysis of various strains as indicated. 15 μg of total cellular RNA was loaded to each lane. Tm RNA (NCTC8325 SRD:S329) is the loading control. V, indicating shuttle plasmid pSK236 control and V + P1*sarA*, indicating 590 bp containing P1 promoter region along with the *sarA* gene. The relative band intensity of various lanes was scanned by Image J software (NIH) and statistical significance was calculated by setting the wild type band intensity as 100. Original blots are presented in Supplementary Material files Fig. X1A,B. (**B**) *teg58* promoter analysis with fusion to GFP reporter system. A 210 bp *teg58* promoter fragment was cloned upstream of the promoterless *gfp*_*uvr*_ gene. Cp standing for chromosomal complementation of the *sarA* mutant. Means and standard deviations from triplicate experiments are shown. Student *t*-test (*P < 0.05, **P < 0.01; *** or ****P < 0.0001). (**C**) PhosphorImager image of 8.0% polyacrylamide gel showing binding of purified SarA protein to 210-bp promoter fragment of *teg58*. ASupplpproximately 0.02 pM of radiolabeled DNA fragment was used in all lanes. For competition assays, 100-fold molar excesses of the unlabeled promoter DNA (x*) and nonspecific competitor DNA (X**, a 200-bp internal fragment of the 16S *rRNA* gene) were added separately as shown. The arrows indicate the free labeled DNA or the DNA–protein complex. Original blot is presented in ementary Material files Fig. X1C.

For subsequent analysis, we have selected two different strain backgrounds, MRSA JE2 and MSSA SH1000. Northern blot analysis (Fig. 2A) showed increased *teg58* transcript level, ~ 4.9-fold increase at exponential and 11.6-fold increase at early post-exponential phases in the *sarA* mutant vs. the wild type while the level was restored to near parental level upon complementation with the shuttle plasmid pSK236::P1*sarA*, consistent with repression of *teg58* transcription by the *sarA* gene. Quantitative promoter activity of *teg58* fused to GFP fluorescence^44^ at the various phases of growth in assorted strains (only shown for the post-exponential phase of growth in Fig. 2B) confirmed northern analysis data. Gel shift assay demonstrated that SarA bound specifically to the *teg58* promoter region since unlabeled *teg58* fragment competed out binding to the labeled DNA, while non-specific 16S promoter fragment did not (Fig. 2C). The calculated binding affinity^45^ (K_d_ = [protein)_50% of DNA bound_] of SarA to the t*eg58* promoter fragment was approximately 0.7 × 10^–6^ M, indicating high binding affinity. Together, these results clearly demonstrated repression of *teg58* transcription by SarA.

### Reduced biofilm formation in *teg58* mutant is not dependent on PIA, *agr* and SarA

**Figure 3:**
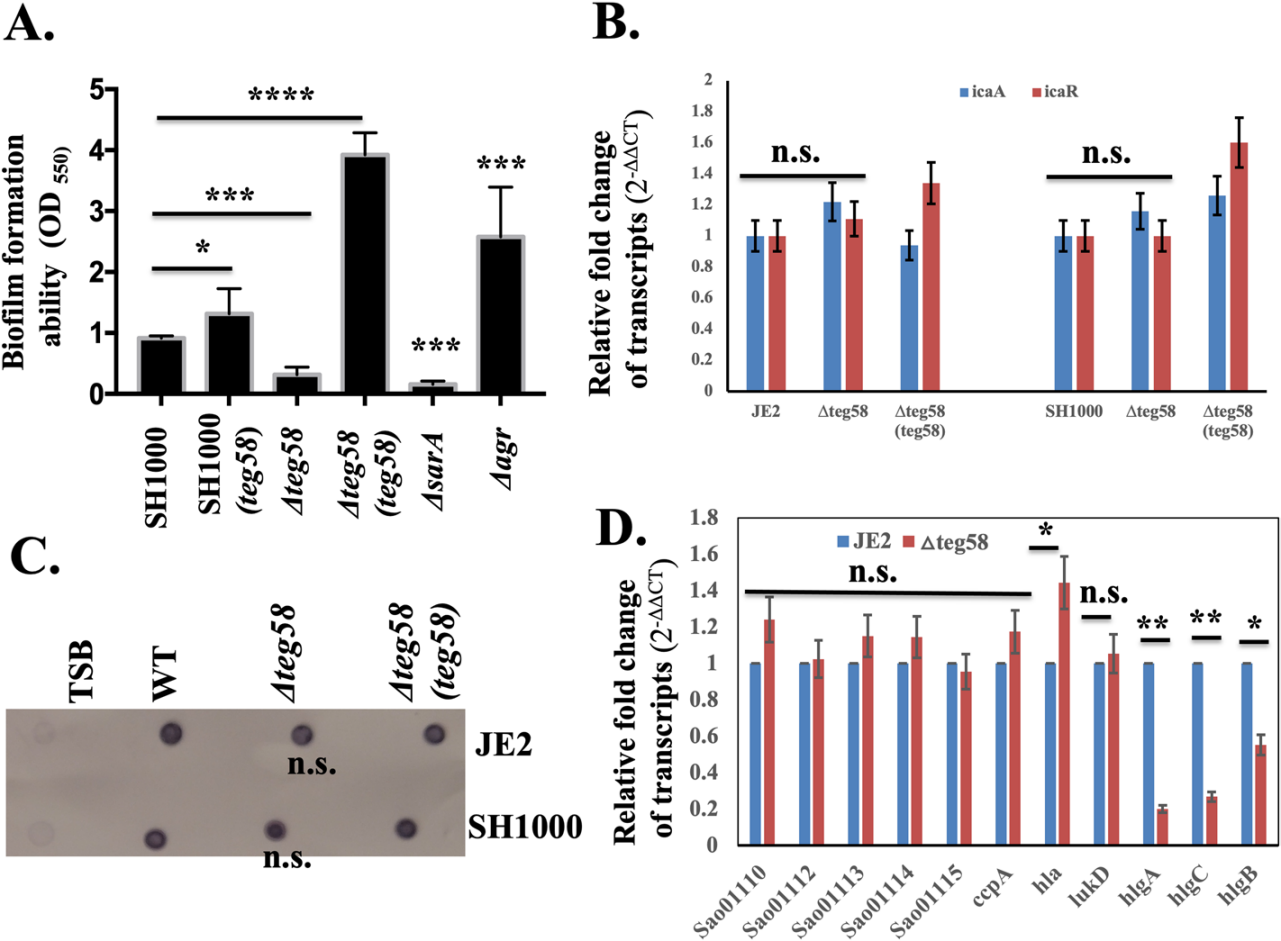
*teg58* mediated biofilm formation is independent of PIA production and regulation of the target genes. (**A**) Static biofilm assay with isogenic strains in SH1000 background in TSB plus 0.25% glucose for 24 h. The *sarA* and *agr* mutant strains were used as controls. Δ*teg58*, 220 bp chromosomal deletion strain; Δ*teg58*(*teg58*), *teg58* mutant complemented with pSK236 containing 450-bp *teg58* with its native promoter. Means and standard deviations from triplicate experiments are shown. Student *t*-test (*P < 0.05, *** or ****P < 0.001). (**B**,**D**) Real-time qRT-PCR analysis for various genes as indicated along with the *rpoB* control were evaluated by qRT-PCR (2^−ΔΔCT^) using total RNA isolated from the respective strains at 24 h grown biofilm cells in TSB. The data were normalized against *rpoB* as the reference transcript for qRT-PCR. Relative expression of the genes was plotted against the wild type set as 1.0. “n.s.” indicate “not significant”. Pairwise comparation with Student *t*-test (*P < 0.05, **P < 0.005). (**C**) Dot bot showing PIA production in various strains. PIA production was detected with an anti-*S. aureus* PIA antibody, showing no changed in PIA production due to the *teg58* deletion compared to the wild type and complement strains. Original blot is presented in Supplementary Material files Fig. X2.

To determine the role of *teg58* in biofilm formation, we have constructed deletion mutants of *teg58* in both JE2 and SH1000 and complemented these mutants with shuttle plasmid pSK236 containing *teg58* fragment (Table S1). As shown in Fig. 3A and Fig. S1C, biofilm was significantly decreased in *teg58* mutants in both backgrounds but increased in *teg58* plasmid complemented strains. Incidentally, optical density (OD600) of *teg58* mutant from supernatants of biofilm wells at 24-h of growth was higher than the parent/complemented strains (Fig. S1D), but cells scraped from biofilm (in triplicates) in wells at 1 and 2-h after inoculation revealed significant reduction of surface attached cells with the *teg58* mutant (53% and 30% of initial input cells at 1 and 2-h, respectively) compared to the wild type (885% and 1142% increased at 1 and 2-h, respectively) (Fig. S1E). To determine if any previously known factors of biofilm formation were affected due to *teg58* deletion, we assayed *icaADBC,* which encodes PIA, and *icaR* transcription in the *teg58* mutant by qRT-PCR analysis and did not find any alternations (Fig. 3B). We also analyzed PIA production by dot blots with anti-PIA antibodies, revealing little or no changes between *teg58* mutant and the parents/complemented strains (Fig. 3C).

Northern blot with a *sarA* probe (Fig. S2A) and Western blot with an anti-SarA monoclonal antibody (Fig. S2B) showed no major differences in *sarA* expression in isogenic *teg58* strains grown under biofilm conditions. The *agr* locus is a negative regulator of biofilm formation indicated that expression of RNAII (*agrDBCA*) and RNAIII did not change in the *teg58* mutant vs. the wild type/complemented strains (Fig. S2C). Transcription of *psm-*α, known to be involved in biofilm dispersal, was also not altered in the *teg58* mutants (Fig. S2C). Analysis of the butanol extract^46^ of filtered cultured supernatants containing PSMs (PSMα1-4, PSMβ1-2, and δ-toxin) by SDS-PAGE (Fig. S2D) revealed no visual changes in intensity of the PSM band in the *teg58* mutant vs. the wild type/complement, consistent with a lack of change in concentration of PSM peptides in the *teg58* mutant vs. isogenic parent as confirmed by Mass Spectroscopy. Analysis for nucleases, proteases, and DNases under control of *agr* and/or *sarA* systems were also not altered in the *teg58* mutants vs. the parent. Together, these results suggest that reduced biofilm formation in the *teg58* mutant does not involve *agr*, SarA, PSMs and other known biofilm inducing factors controlled by SarA and *agr*.

### RNA-Seq analysis of the *teg58* mutant

Based on the above studies, we hypothesized that *teg58* may regulate uncharacterized gene products involved in biofilm formation in *S. aureus*. To determine target genes regulated by *teg58* and the ensuing phenotypic involvement (e.g., biofilm formation) in *S. aureus*, we performed RNA-Seq analysis of various strains in SH1000. To detect differentially expressed transcripts between various strains, pairwise comparisons were performed, and statistical significance (FDR-adjusted P < 0.05) determined using the edgeR package^47^ as described previously^24^. Phage-encoded genes were eliminated from analysis. Based on a twofold change, transcriptional profiling revealed alteration of 116 genes (38 genes up-regulated and 78 genes down-regulated) in the *teg58* mutant vs. the wild type whereas a threefold change would yield 43 affected genes (8 up- and 35 down-regulated) in *teg58* deletion (Table 1 and Table S3). The altered genetic pathways include purine metabolism, arginine biosynthesis, transport systems, metabolic and virulence genes. Some of *teg58* neighboring genes (Fig. S1A, Table S4) including virulence factor were altered in the RNA-Seq dataset. Some of the virulence including neighboring genes (e.g., Saoushc_-01121/*hla*, -01954/*lukD*, -02708-10/*hlgA, C* and *B*, _01110/*efb*-1*,* -01112/*flipr*, -01113/HP, -01114/*efb*, -01115/*scip*) were confirmed by qRT-PCR (Fig. 3D). The data indicated that no significant alternation in transcript levels of the neighboring genes, although we are not ruling out the effect of *teg58* at the post-transcription stage, which needs further investigation.

**Table 1:** Selected *teg58*-regulated genes.

Gene designation	Gene name or description	Wild type SH1000 expression value, RPKM	ALC8717, Teg58 mutant expression value, RPKM	Fold Repressed; mut/wt	Fold activated; wt/mut
**Purine metabolic pathway**					
SAOUHSC_00019	Adenylosuccinate synthase, *purA*	1208	3919	3.24	
SAOUHSC_01016	Phosphoribosylglycinamide formyltransferase, *purN*	5369	12,434	2.31	
SAOUHSC_01017	IMP cyclohydrolase, *purH*	5769	14,143	2.45	
SAOUHSC_01018	Phosphoribosylamine glycine lipase, *purD*	6179	18,501	2.99	
SAOUHSC_01015	Phosphoribosylformylglycinamidine , *purM*	4763	9490	2.0	
SAOUHSC_01742	GTP pyrophosphokinase, *relA*	5431	13,658	2.51	
SAOUHSC_00373	Xanthine permease, *pbuX*	7804	1103		7.07
SAOUHSC_00372	Xanthine phosphoribosyltransferase, *xprT*	4545	769		5.91
SAOUHSC_02368	CTP synthase, *ctrA*	4998	12,820	2.56	
**Arginine biosynthesis pathway**					
SAOUHSC_00898	Argininosuccinate lyase, *argH*	9081	46,012	5.06	
SAOUHSC_00899	Argininosuccinate synthase, *argG*	6770	31,658	4.67	
SAOUHSC_01787	Arginine permease, *lysP*	42,011	10,666		3.94
SAOUHSC_02558	Urease subunit gamma, *ureA*	2734	641		4.26
SAOUHSC_02559	Urease subunit beta, *ureB*	3408	737		4.62
SAOUHSC_02561	Urease subunit alpha, *ureC*	3464	917		3.78
SAOUHSC_02565	Urease accessory protein, *ureD*	3966	1380		2.87
SAOUHSC_02562	Urease accessory protein, *ureE*	5208	1546		3.37
SAOUHSC_02563	Urease accessory protein, *ureF*	4050	1188		3.41
SAOUHSC_02564	Urease accessory protein, *ureG*	4811	1747		2.75
**Valine, leucine, and isoleucine biosynthesis pathway**					
SAOUHSC_02281	Dihydroxy-acid dehydratase, *ilvD*	147	501	3.41	
SAOUHSC_02282	Acetolactate synthase large subunit, *ilvB*	246	804	3.27	
SAOUHSC_02284	2-dehydropantoate 2-reductase, *ilvC*	315	906	2.88	
SAOUHSC_02283	Acetolactate synthase small subunit, *ilvH*	283	671	2.37	
SAOUHSC_02289	Threonine dehydratase catabolic, *ilvA*	1170	2698	2.3	
**Glycine, serine and threonine metabolism**					
SAOUHSC_02716	Dethiobiotin, *bioD1*	107	230	2.15	
SAOUHSC-02932	Choline dehydrogenase, *betA*	4988	879		5.67
SAOUHSC_02933	Betaine aldehyde dehydrogenase, *gbsA*	11,652	2229		5.22
**Virulence factors**					
SAOUHSC_01121	alpha-hemolysin, *hla*	3247	8832	2.72	
SAOUHSC_00069	Immunoglobulin G binding protein A, *spa*	1108	3256	2.94	
SAOUHSC_02710	Leukocidin S subunit, *hlgC*	3150	664		4.75
SAOUHSC_02709	Gamma-hemolysin component B, *hlgB*	3179	765		4.15
SAOUHSC_02708	Gamma-hemolysin component A, *hlgA*	2952	766		3.85
SAOUHSC_00051	Phospholipase C, *plC*	30,747	9699		3.17
SAOUHSC_02163	Beta-hemolysin, *hlb*	29,940	9333		3.21
SAOUHSC_02167	Involved in expression of fibrinogen binding protein, *scn_3*	409	99		4.12
**Cell wall, transporters, and membrane proteins**					
SAOUHSC_02901	Membrane spanning protein	23,101	2999		7.7
SAOUHSC_02888	Membrane spanning protein	275	847	3.08	
SAOUHSC_02767	Oligopeptide transporter putative substrate binding domain protein,*opp-1A*	948	3343		3.52
SAOUHSC_02766	Oligopeptide transporter membrane permease domain protein,*opp-1B*	577	1766		3.06
SAOUHSC_00235	PTS system, IIA component, *cmtB*	1995	479		4.16
SAOUHSC_00311	PTS system transporter subunit IIB	2817	769		3.66
SAOUHSC_02866	Putative antibiotic transport-associated protein, *mmpL8*	2457	499		4.91
SAOUHSC_00136	ABC transporter ATP-binding protein, *ssuB_1*	394	97		4.06
SAOUHSC_00138	ABC transporter permease protein, *ssuC*	515	123		4.17
SAOUHSC_02773	Aminobenzoyl-glutamate transport protein, *abgT*	6507	1928		3.37
SAOUHSC_02130	SecD -like transglycosylase, s*ceD*	710	204		3.49
**Metabolic enzymes**					
SAOUHSC_00135	Alpha-helical coiled-coil protein , *srpF*	2371	625		3.79
SAOUHSC_00299	Nucleoside recognition domain-containing protein	2492	10,462	4.2	
SAOUHSC_00113	Acetaldehyde dehydrogenase, *adhE*	6858	25,673	3.74	
SAOUHSC_00412	NADH dehydrogenase, subunit 5, *ndhF*	11,703	1775		6.59
SAOUHSC_00139	Acyl-CoA dehydrogenase	1066	341		3.13
SAOUHSC_00075	Siderophore staphylobactin biosynthesis protein, *sbnA*	92	21		4.41
**Transcriptional regulatory proteins**					
SAOUHSC_02271	Transcriptional regulator, *yedF*	1214	268		4.53
SAOUHSC_00706	Fructose repressor, *glcR*	18,040	5240		3.44
SAOUHSC_02570	Transcription regulator, HP	2238	580		3.86
**Uncharacterized proteins**					
SAOUHSC_00410	Uncharacterized conserved protein	19,136	2907		6.58
SAOUHSC_00355	Uncharacterized conserved protein, *yxeA*	192	41		4.63

### Impact of *teg58* on l-arginine biosynthesis and production

**Figure 4:**
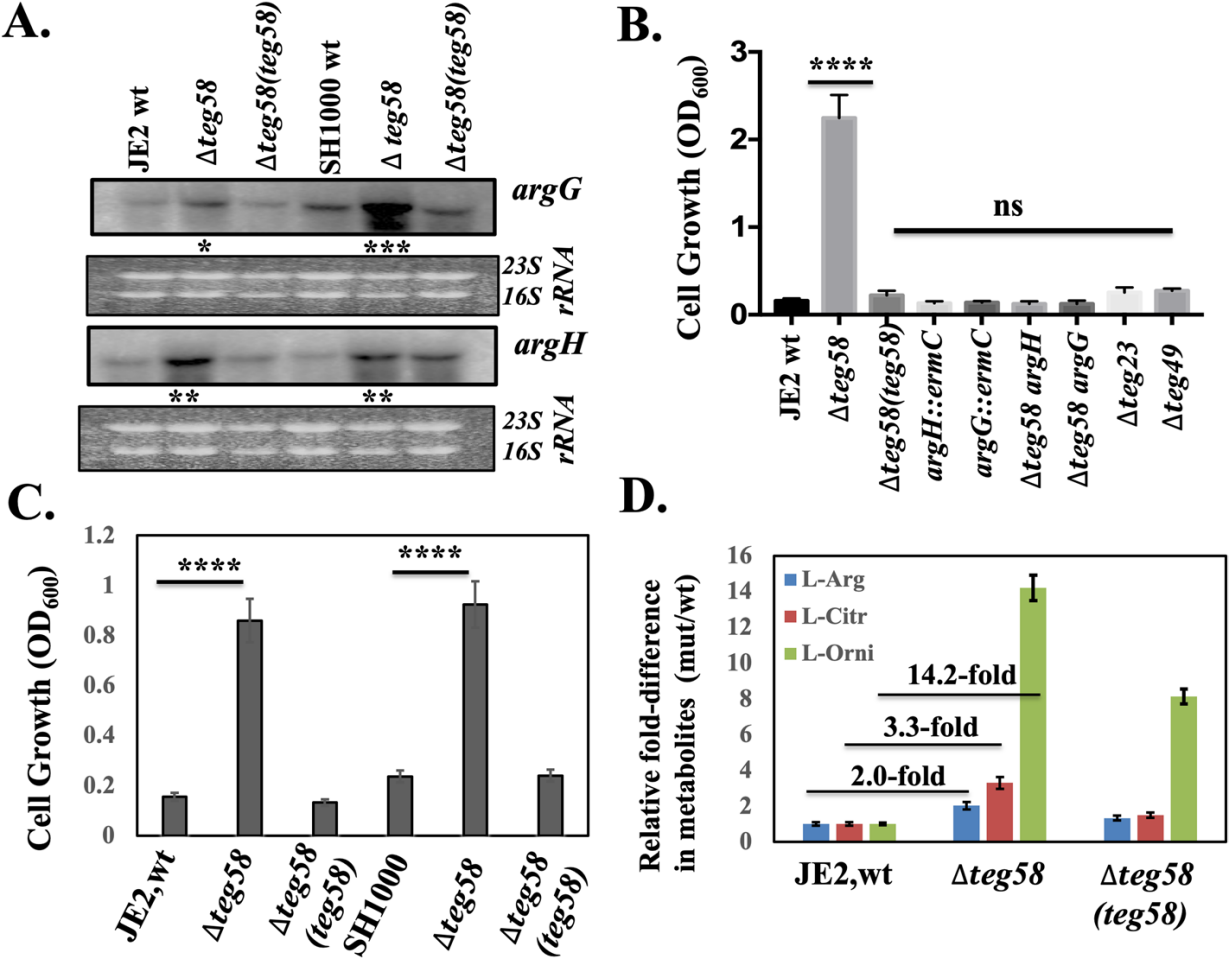
*teg58* effects l-arginine biosynthesis. (**A**) Northern blot analysis for various *teg58* derivative strains with *argG,* and *argH* probes representing genes involved in arginine biosynthesis pathway. A total of 10 μg of cellular RNA isolated from the post-exponential phase of growth was loaded onto each lane. Ethidium bromide-stained gel used for blotting, showing 16S and 23S rRNA bands, is shown as the loading control. The relative band intensity of various lanes was scanned by Image J software (NIH) and the statistically significant was calculated considering the wild type band intensity set as 100. Original blots are presented in Supplementary Material files Fig. X3A–C. (**B**,**C**) Growth analysis in CDM containing 1% glucose without supplemented l-arginine for various isogenic strains as indicated. Growth was performed for 48 h at 37 °C in an incubator shaker (**B**; aerobic) and incubated in filled and sealed 50 ml screw capped tubes (**C**; anaerobic). (**D**) Determination of comparative intracellular l-arginine, l-citrulline, and l-ornithine concentrations of the wild type and the *teg58* mutant of JE2 strains grown under aerobic conditions in TSB by LC–MS/MS mass spectroscopy. The asterisks indicate statistical significance between wild-type and isogenic mutant or various strains, determined using Student *t*-test (*, *P* < 0.05; **, *P* < 0.005; *** or ****, P < 0.0001) or “n.s” indicate “not significant”.

RNA-Seq analysis indicated that *argG* (4.6-fold)*,* and *argH* (5.0-fold), as part of an operon, were significantly upregulated while *ureA-G* encoding urease subunits (converting urea to ammonium as a downstream catabolism event) were down regulated (2.7–4.6-fold) in the *teg58* mutant (Table 1). Genes involved in arginine catabolism such as *rocF* (~ 1.0-fold), *argF* (~ 1.12-fold), *arcA* or *arcB* (~ 1.5-fold) were not markedly altered in the *teg58* mutant (Table S4). Northern analysis indicated a significant increase in *argGH* transcript level in the *teg58* mutant (~ 1.8–5.5 fold in Fig. 4A) vs. parent but returned to parental level upon complementation (Fig. 4A). Growth analysis in CDM^42^ without l-arginine (containing 1% glucose) for 48 h (Fig. 4B) indicated that the *teg58* mutant grew well while the wild type, isogenic complement as well as control sRNA mutants (*teg49* and *teg23*) grew poorly in this arginine-depleted medium. As expected, the double ∆*teg58*/*argG/H* or *single argG/H*::*erm*C mutants failed to grow in CDM without l-arginine, suggesting that enhanced *argGH* expression for arginine biosynthesis in the *teg58* mutant is likely responsible for growth in arginine deficient medium (Fig. 4B). Under static anaerobic (Fig. 4C) conditions, the *teg58* mutant retained growth pattern like the aerobic setting. Growth analysis of the isogenic *teg58* strains in TSB (Fig. S3A) and CDM (Fig. S3B) revealed no growth defect due to deletion or complementation. We also found no alternation in transcription of *ccpA*^22,35^ known to activate biofilm formation in a glucose-rich environment (Fig. 3D) and is involved in the biosynthesis of arginine via the urea cycle utilizing proline as the substrate.

For confirmation, ultra-high performance liquid chromatography—high resolution tandem mass spectrometry was used to analyze l-arginine and its metabolite using intracellular fractions of isogenic *teg58* strains grown under aerobic condition (Fig. 4D). The data indicated ~ twofold increase in intracellular l-arginine concentration in the *teg58* mutant compared to the wild type, correlating with increased *argGH* transcription and higher growth in arginine-deficient CDM for the *teg58* mutant.

### Role of l-arginine in biofilm formation in *S. aureus*

**Figure 5:**
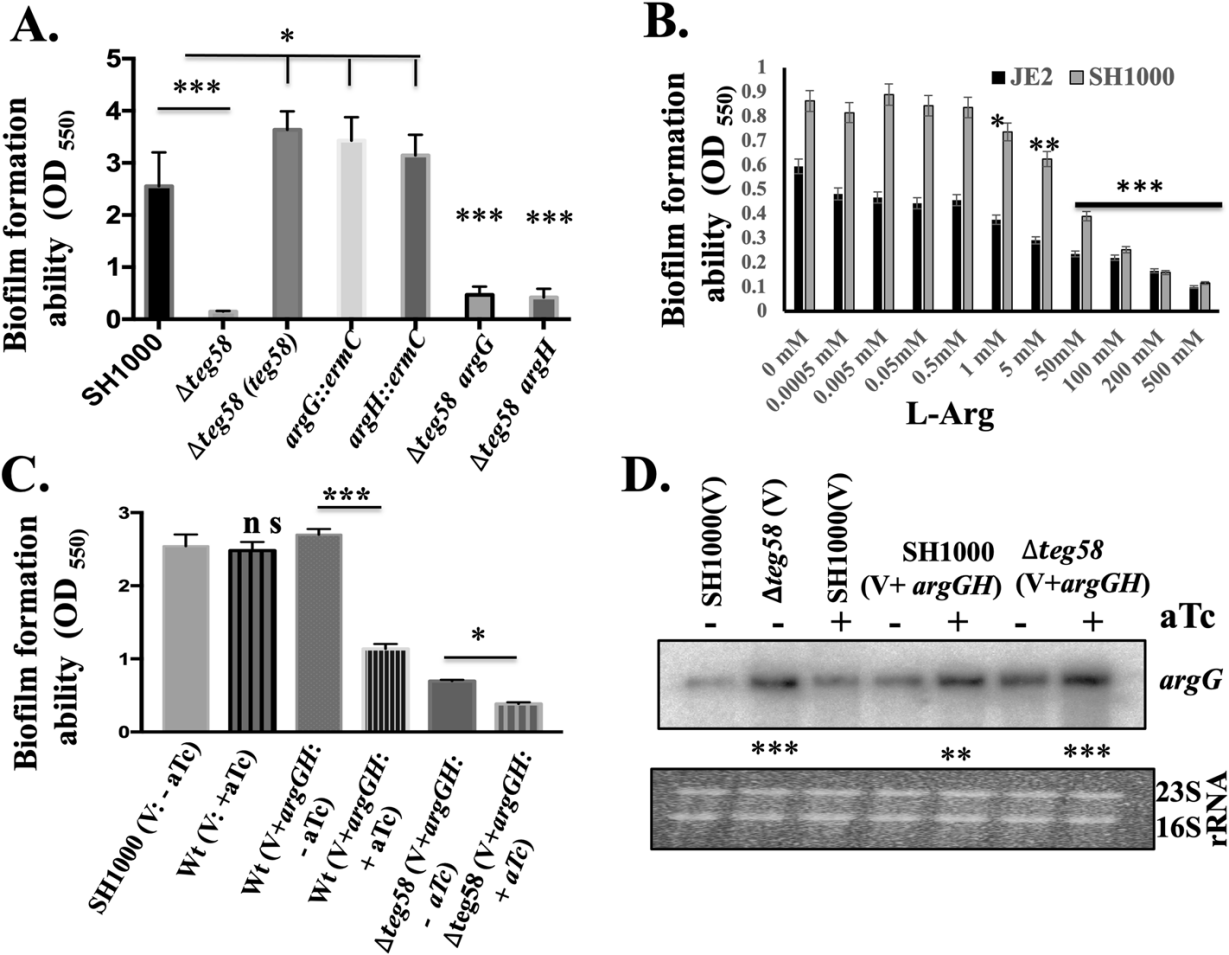
Inhibition of biofilm formation by l-arginine. (**A**) Static biofilm formation for various strains in complete CDM containing 1% glucose for 24 h in a microtiter plate (Costar, USA). (**B**) Biofilm formation for various strains for 24 h in complete CDM containing 1% glucose and various concentrations of exogenous l-arginine in a microtiter plate format. (**C**) Biofilm formation for various strains as indicated in TSB supplemented with 0.25% glucose for 24 h in a microtiter plate under induced (+ ATc) (500 ng/ml) and uninduced (-ATc) conditions. V represents pALC2073 as the vector control; V + *argGH*, represents pALC2073 with *argGH* including ribosome binding site cloned under *tetO*-inducible promoter; ATc stands anhydrotetracycline which is inducer for the pALC2073 system. (**D**) Transcript analysis of 10 μg of total cellular RNAs isolated from cultures in (**C**) under various conditions and probed with *argG* probe. “+” and “−” indicate the inducible and uninducible conditions. The relative band intensity of various lanes was determined by Image J software (NIH) and the statistically significant was calculated considering the wild type as 100. Original blot is presented in Supplementary Material files Fig. X4. Means and standard deviations from triplicate experiments are shown. Student *t*-test (*P < 0.05, **P < 0.01; *** or ****P < 0.001) and “n.s” indicate “not significant”.

l-arginine plays a critical role in growth and biofilm formation in several bacterial species by neutralizing acidic pH, inhibiting biofilm formation, and preventing co-aggregation of cells^32,33,48^. However, the role of arginine in biofilm formation in *S. aureus* has not been defined. Biofilm assays with assorted *teg58* mutants in complete CDM (containing 1% glucose) found similar biofilm defect (Fig. 5A) as seen in TSB (Fig. 3A). Importantly, inactivation of *argG* or *argH* also led to increased biofilm formation (Fig. 5A) while the double mutants (∆*teg58*/*argG* or ∆*teg58*/*argH*) exhibited reduced biofilm formation, akin to the ∆*teg58* mutant, implying the role of additional factor(s) other than *argGH* that may be involved in reducing biofilm formation in the *teg58* mutant. In fact, our RNA-Seq analysis (Table 1) indicated that *relA,* involved in stress-responsive alarmone (p)ppGpp production and biofilm dispersion in multiple bacterial species^49^ including *S. aureus*^50^, or l-arginine dependent factors yet to be identified, could also be involved in reducing biofilm formation in the *teg58* mutant. We are actively investigating the role of *relA* in biofilm formation in *S. aureus* as well as in combination with *argGH* in the *teg58* mutant. Another possibility could be increased *argGH* mRNA that binds to *teg58*, therefore, decreases titers of free *teg58*. Decreasing *teg58* titers alters the abundance of an alternative mRNA that *teg58* interacts with, resulting in increased biofilm formation or increasing biofilm dispersal. Addition of l-arginine to CDM (Fig. 5B and Fig. S4C) or TSB (Fig. S4A,B) also reduced biofilm formation, with increasing concentrations l-arginine, beginning at ~ 1 mM (Fig. 5B).

To verify the role of intracellular arginine in repressing biofilm formation, we overexpressed *argGH* genes in a *tetO*-inducible vector, pALC2073^51^, under induced and uninduced condition (Fig. 5C and Fig. S4D). Results indicated a noticeable decrease in biofilm formation in the parent under induced vs. uninduced condition, correlating with significant induction of the *argGH* transcript as detected by Northern blots in induced cells (Fig. 5D). Growth analysis in CDM lacking arginine indicated that the wild type grew faster under induced than uninduced condition, implying overexpression of intracellular *argGH* leads to enhanced cell growth (Fig. S3C). Overall, these analyses showed that extracellular l-arginine and/or intracellular overexpression of *argGH* can repress biofilm formation in *S. aureus*.

### Specific interaction between sRNA *teg58* and *argGH* mRNA for arginine production

**Figure 6:**
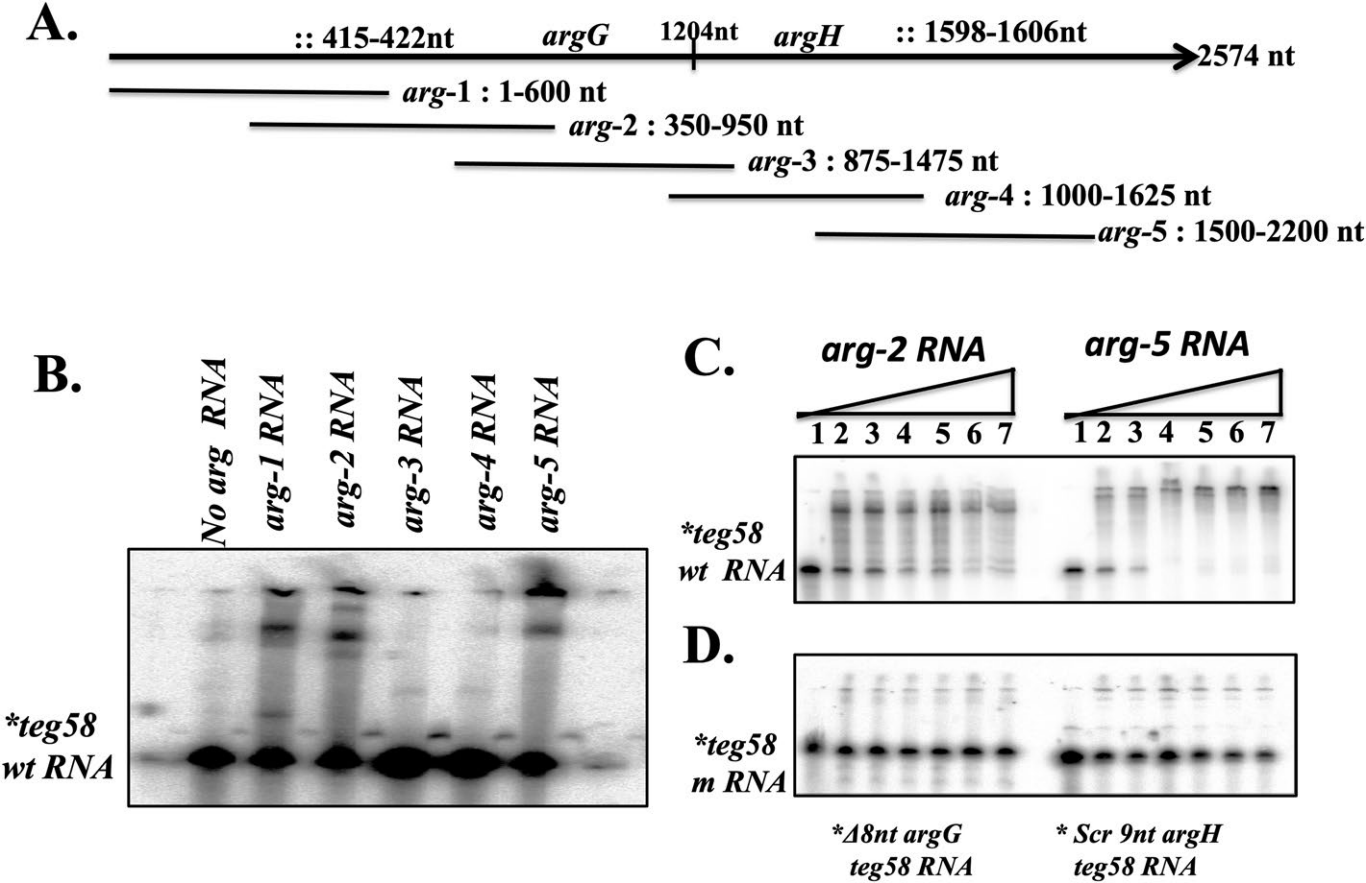
Potential sRNA::mRNA interactions in vitro between *teg58* and *argGH* transcripts. (**A**) Representation of various truncated fragment of *argGH* along with site of interactions used for mobility shift experiments. The symbol “::” denotes potential sites of interaction between *teg58* and *argGH* mRNA. (**B**) Gel mobility shift assays of *teg58* RNA binding to various truncated *argG* or *argH* RNA fragments. Samples containing 5’ end-labeled 170-nt long transcript of *teg58* RNA (1 nM) and tenfold molar excess of different truncated region of *argGH* RNA were heated to 70 °C and slowly cooled to room temperature, loaded onto 6% native polyacrylamide gel containing 5% glycerol, run at 4 °C, dried and phosphorimaged. The *arg*-1 and *arg*-2, and *arg*-5 truncated region of *argGH* mRNA, corresponding to coding regions of the *argG* and *argH* genes, respectively, interacted with *teg58* sRNA in a manner consistent with in silico analysis, while two truncated fragments that were predicted not to interact did not cause any mobility shift. (**C**) Phosphorimage of a 6% nondenaturing polyacrylamide gel showing the titration of 5’ end-labeled *teg58* RNA fragment with increasing concentrations of truncated *argGH* RNA fragments. Lanes 1–7, mobility of the RNA band in the presence of 0, 10, 20, 50, 100, 200, and 300 -fold molar excess of the *arg*-2 or *arg*-5 RNA. (**D**) Phosphorimage of a 6% nondenaturing polyacrylamide gel showing the titration of 5’end-labeled *teg58* fragment as indicated with increasing concentrations of mutated *argGH* RNA fragments in a manner analogous to panel C. Original blots for B,C, and D are presented in Supplementary Material files Fig. X5A–C.

To determine whether *teg58* affects *argGH* at the transcriptional or post-transcriptional level, we performed *gfp*_*uvr*_ reporter gene fusion with the *argGH* promoter (P_*argGH*_::*gfp*_uvr)_ in pALC1484^44^ in isogenic *teg58* strains, showing no significant differences in mean GFP fluorescence per OD_600nm_ values between the wild type and *teg58* mutant strains of JE2 (15,464 ± 800 vs. 16,350 ± 760) and SH1000 (10,604 ± 612 vs. 9897 ± 700), indicating that *teg58* does not regulate *argGH* transcription. In silico analysis using IntaRNA tool^41^ to locate potential site for sRNA-mRNA interactions (Fig. 6A and Fig. S5) revealed one 8-nt site (nt 51–58 in *teg58*) (Fig. 1C and Fig. S5A) with exact match to *argG* (nt 415–422 from start codon) and another 39-nt including gaps (nt 104–142 in *teg58*) (Fig. 1C and Fig. S5B) for plausible interaction with *argH* (nt 403–435 from the *argH* start codon). Notably, 9-nt within this site in *argH* (nt 403–411 or nt 1598–1606 in the *argGH* transcript) showed perfect pairing with *teg58* (nt 134–142). Since the entire *argGH* transcript, at ~ 2600-nt long, is not suitable for resolution by mobility shift assay^52,53^, hence, we have divided the *argGH* transcript into five different overlapping fragments (*arg*-1 (1–600-nt), *arg*-2 (350–950 nt), *arg*-3 (875–1475 nt), *arg*-4 (1000–1625 nt), and *arg*-5 (1500–2200) (Fig. 6A). We conducted mobility shift assays with a radiolabeled ~ 170 nt *teg58* fragment and larger fragments of *argGH* mRNAs as cold interacting RNAs. As shown in Fig. 6B, only *arg*-1, *arg*-2 and *arg*-5 RNA fragments showed interacting bands with the native *teg58* RNA, in good agreement with the *in-silico* prediction of sites of interaction. In contrast, *arg*-3 and *arg-*4 mRNA fragments lacking homology with *teg58* did not bind to *teg58* in the assay. The *teg58* probe also shifted in a concentration dependent manner with *arg-2* and *arg-5* RNA fragments (Fig. 6C). When the same experiments were performed with *teg58* probe lacking the 8-nt interaction site for *argG* or scrambled 9-nt interaction site of *argH* for *teg58* (without disrupting significant *teg58* structure; Fig. S5D,E), we did not detect any interacting bands (Fig. 6D), suggesting that *teg58*-*argGH* interaction is specific in vitro.

### *Teg58* and *argGH *interactions validated in vivo growth and biofilm formation, and *argGH* mRNA stability

**Figure 7:**
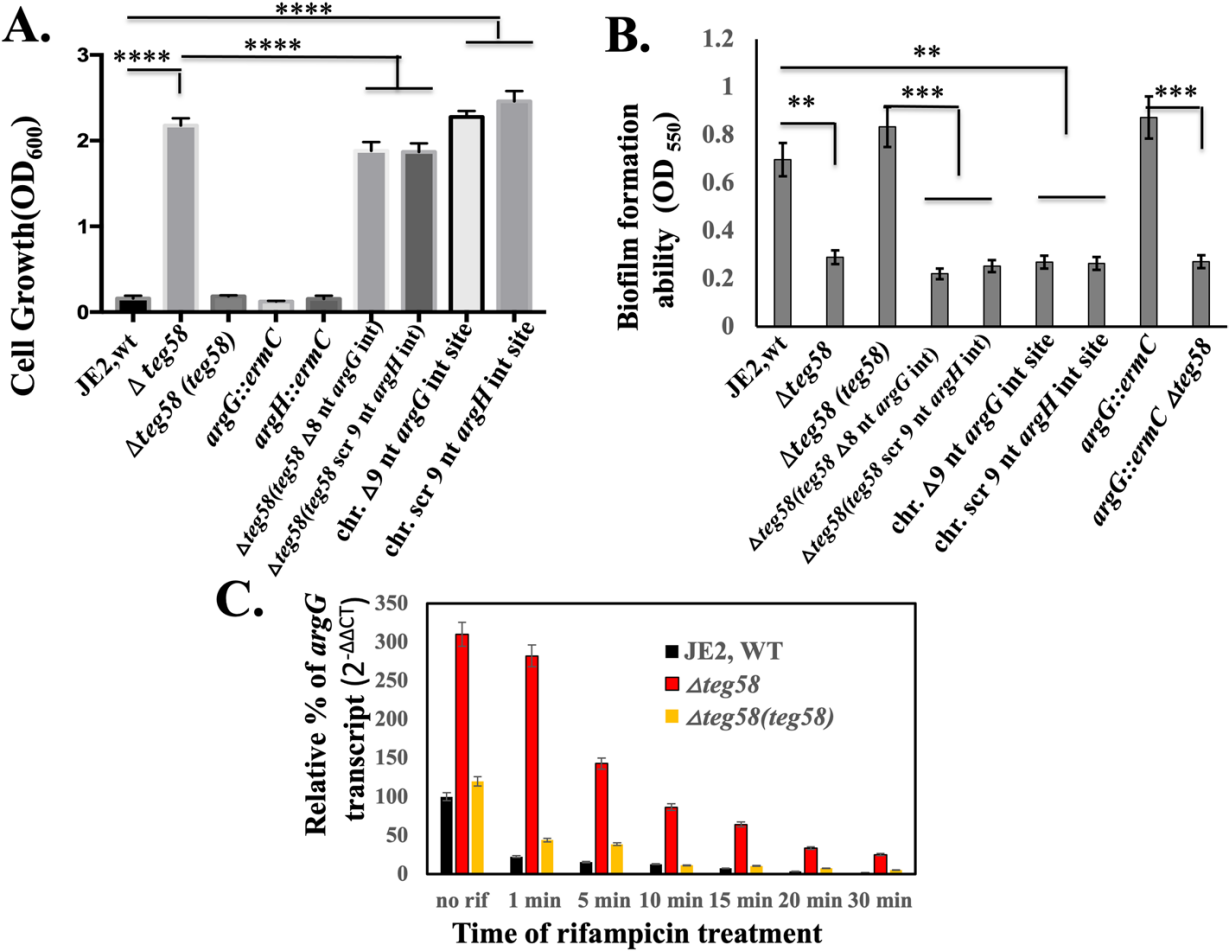
In vivo validation of potential sRNA::mRNA interactions between *teg58* and *argGH* transcript and mRNA stability in *S. aureus* cells. (**A**) Growth analysis in CDM containing 1% glucose without supplemented l-arginine for various isogenic strains as indicated. These strains include the wild type JE2, isogenic *teg58* mutant; *teg58* mutant complemented with native *teg58*, *argG::ermC*; *argH::ermC; teg58* mutant with shuttle vector pSK236 carrying *teg58* with the deletion of 8-nt interaction site for *argG*; *teg58* mutant with shuttle vector pSK236 carrying *teg58* with the 9-nt interaction site for *argH* scrambled; chromosomal mutation of the *argG* interaction site in *teg58* (9-nt or 3 codons/AFA deletion); chromosomal mutation of the *argH* interaction site in *teg58* (9-nt scrambled or SLQ to SLT). (**B**) Biofilm formation for various strains as indicated and described in (**A**) in TSB with added 0.25% glucose for 24 h in a microtiter plate. (**C**) *argG* mRNA stability in various strains was evaluated by qRT-PCR (2^−ΔΔCT^) using total RNA isolated from respective strains at the exponential phase of growth after treatment with rifampicin (200 μg per mL) at different time points. The data were normalized against *rpoB* as the reference transcript for the qRT-PCR. Relative expression of the gene (*argG*) in the wild type or *teg58* mutant was plotted against no rifampicin treatment set as 100%. Means and standard deviations from triplicate experiments are shown. Student *t*-test (**P < 0.01; *** or ****P < 0.001).

To verify *in* vivo RNA-RNA interaction between *teg58* and *argGH* mRNAs, we complemented the *teg58* mutant with pSK236 containing *teg58* fragment but with a deletion of 8-nt putative *argG* or a scrambled 9-nt *argH* interaction site followed by growth and biofilm analysis. Both mutated *teg58* fragments on a plasmid mirrored the growth of the *teg58* mutants in CDM without l-arginine (Fig. 7A) whereas the native *teg58* fragment phenocopied the wild type. Chromosomal deletion of the 9-nt of *argG* or scrambling 9-nt site (coding for SLT instead of SLQ) of *argH* for interactions with *teg58* in JE2 yielded growth in CDM lacking l-arginine (Fig. 7A), thus mimicking the growth pattern of the *teg58* mutant instead of the wild type. Thus, the predicted interactions between *teg58* and *argGH* mRNA are important to prevent production of arginine for growth in media lacking exogenous l-arginine.

Besides growth, we also ascertained biofilm formation. As shown in Fig. 7B, the *teg58* mutant, harboring the recombinant pSK236 carrying *teg58* with specific *argG* or *argH* mutation or chromosomal mutation of *argG* or *argH* at the putative interaction sites, failed to restore biofilm formation to the level of the parent. In addition, RNA stability studies for the *argGH* transcript in isogenic *teg58* strains by qRT-PCR (Fig. 7C) clearly showed that the *argGH* transcript is more stable in the *teg58* mutant than the wild type/complemented strains. The *argGH* mRNA half-life, a parameter for RNA stability, as defined by reduction of the transcript abundance by a factor of two, was determined to be ~ 1 min in the wild type and about ~ 5 min in the *teg58* mutant. Taken together, these results from our in vitro and in vivo studies strongly suggested that failure to base-pair *teg58* with *argGH* mRNA due to mutation stabilizes the transcript and increases arginine biosynthesis and production which represses biofilm formation in *S. aureus.*

### *Teg58*-mediated biofilm production is independent of l-arginine catabolism

**Figure 8:**
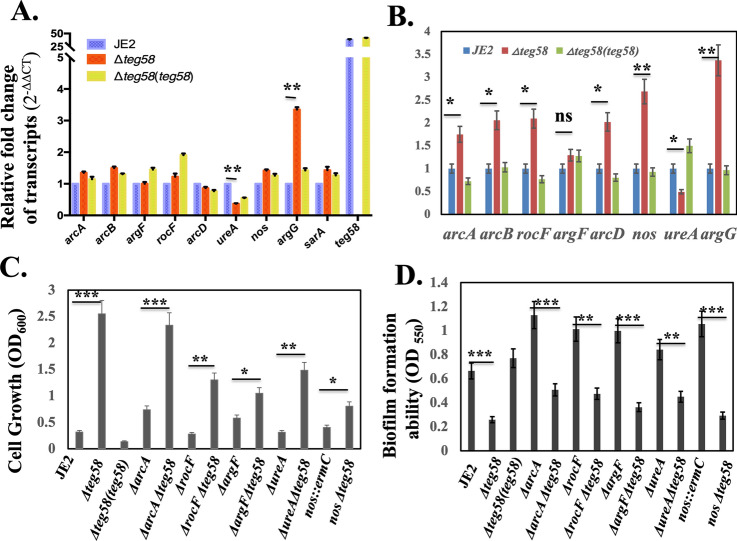
Analysis for the l-arginine catabolic pathway genes in *teg58*-mediated biofilm formation. (**A**,**B**) Real-time qRT-PCR analysis, along with the *rpoB* control, were evaluated by qRT-PCR (2^−ΔΔCT^) using total RNA isolated from the respective strains at 24 h grown biofilm cells (**A**) and post-exponential phase of aerobically grown cells (**B**) in TSB. The data were normalized against *rpoB* as the reference transcript for qRT-PCR. Relative expression of the genes was plotted against the wild type set as 1.0. (**C**) Growth analysis in CDM lacking l-arginine for various arginine catabolic pathway mutant strains as indicated. Growth was performed for 48 h at 37 °C in an incubator shaker. (**D**) Biofilm formation for various strains in TSB with 0.25% glucose for 24 h in a microtiter plate. Means and standard deviations from triplicate experiments are shown. Student *t*-test (*P < 0.05, **P < 0.01; ***P < 0.001) and “n.s” indicate “not significant”.

We have established above that reduced biofilm formation in the *teg58* mutant is due, at least in part, to increased expression of *argGH* and ensuing production of l-arginine. As arginine level is balanced by biosynthesis and catabolism, we wanted to determine if reduced arginine catabolism contributes to the observed phenotype in the *teg58* mutant. Analysis by qRT-PCR of the putative catabolism genes including *arcA, arcB,* and *arcD* (antiporter) (transcribed as a single transcript), *argF* and *rocF* from biofilm grown cells revealed no significant differences (0.8 to 1.5-fold) between the *teg58* mutant and the parent except for reduced *ureA* expression (~ 0.38 fold) (Fig. 8A) in the *teg58* mutant. Failure to restore the *ureA* transcript in the complemented *teg58* strain to wild type level as detected by qRT-PCR (Fig. 8A) suggested that regulation of the *ureA*/urease pathway genes by *teg58* may be different from the classic arginine catabolism pathway. Results also indicated that *argG* and *sarA* as well as *teg58* (~ 38 fold in the wild type vs. mutant) remained active in biofilm cells (Fig. 8A). We also found no significant alterations in intracellular l-citrulline concentrations, the catabolite of ArcA, ArgF and Nos enzymes, between biofilm cells of the wild type and the *teg58* mutant using a colorimetric microtiter plate method^54^ (Table 2). In contrast, the ratio of citrulline content in *teg58* mutants vs. wild types (JE2 and SH1000) grown under aerobic shaking condition revealed an increase of 1.7 and 2.2-fold, respectively, consistent with the data from UHPC-HRMS/MS (Fig. 4D). To confirm that increased l-citrulline level in aerobic or planktonic cells is associated with elevated transcript level of the arginine catabolism genes, qRT-PCR of aerobic grown cells in TSB was performed (Fig. 8B), showing elevated transcript levels (1.2–2.7 fold) of *arcA* (1.7-fold), *arcB, rocF*, and *arcD* (~ 2.0-fold), *argF* (1.2-fold), *argG* (~ 3.0-fold), and *nos* (~ 2.7-fold) in the *teg58* mutant, consistent with increased l-citrulline (Table 2) and l-ornithine levels (Fig. 4D) in cells grown under aerobic conditions. As aerobic and biofilm growth conditions (with glucose) are diverse, these data demonstrated that the catabolic pathway genes are relatively quiescent in biofilm cells but are more active in aerobic growth.

**Table 2:** Estimation l-citrulline in various *S. aureus* strains.

Strains	Normal aerobically grown cells µM/mg proteins	Biofilm cells µM/mg proteins
JE2, wt	95.92 ± 14	238.52 ± 22
JE2∆*teg*58	163.21 ± 23	246.66 ± 20
JE2∆*teg58 (teg58*)	73.82 ± 18	252.59 ± 18
SH1000	60.74 ± 20	109.87 ± 12
SH1000∆*teg58*	132.09 ± 16	113.08 ± 10
SH1000∆*teg58 (teg58*)	68.88 ± 21	91.85 ± 20

To further investigate the role of catabolic genes in arginine pathways, we have constructed single and double mutants of *arcA, rocF, argF*, *nos,* and *ureA* along with the *teg58* mutation in JE2. Analyses of growth in CDM lacking l-arginine showed that the single mutant *arcA*, and *argF* mutants grew poorly but slightly higher than the parent while the *rocF* mutant grew even slower. Notably, there was no growth difference in the *ureA* and *nos* mutant vs. the parent (Fig. 8C). Importantly, the double mutants all showed increased growth as compared to the single *arcA, rocF, argF, nos,* and *ureA* mutants, but less than that of the single *teg58* mutant (Fig. 8C). About biofilm formation, these single catabolic mutants showed higher biofilm formation in TSB compared to the wild type (Fig. 8D), consistent with reduced activity of the catabolic pathway, whereas the double mutants with *teg58* exhibited significant reduction in biofilm formation compared to the respective single mutant strains. These results clearly suggest that decreased biofilm formation is due to the inactivation of *teg58* rather than the deletion of the arginine catabolic pathway genes. Deleting the *ureA* gene linked to the production of ammonium (NH_4_^+^) from urea and downstream from an arm of the arginine catabolic pathway enzymes, led to mild increase in biofilm formation in the *ureA* mutant vs. the parent (Fig. 8D). Overall, these results showed that the arginine catabolic pathway genes are not likely to impart any major role in *teg58*-mediated biofilm formation, but the arginine biosynthesis pathway genes do.

## Discussion

In this report, we have identified a small regulatory RNA, *teg58*, by using ChIP-Seq and RNA-Seq analysis and characterized *teg58* that involved in biofilm formation in *S. aureus.* Remarkably, biofilm formation repressed by *teg58* is not possibly tied to known and tested protein-based factors. In addition, factors involved in maturation of biofilm and detachment are not significantly altered in the *teg58* mutant (Table 1 and Table S3).

l-arginine is a well-characterized precursor molecule for the biosynthesis of other amino acids and serves as an alternative energy source during anaerobic metabolism^38^. Various analyses disclosed that the *argGH* transcript was significantly upregulated in the *teg58* mutant. Indeed, growth analysis with and without exogenous arginine as well as arginine determination in *teg58* mutant are consistent with this observation. In silico analysis followed by biochemical and genetic studies strongly implicates specific sRNA-mRNA interaction between *teg58* and *argGH* mRNA for arginine production in the *teg58* mutant. Thus, biosynthesis of l-arginine is one of the major metabolic factors in the repression of biofilm formation in the *teg58* mutant vs. the parent whether directly or indirectly which yet to be identified.

We also ruled out the possibility that reduced catabolism of arginine would contribute to increased arginine level. Most of the genes in the chromosomal *arc* operon or a second copy of the arginine deiminase pathway (ADI) in the ACME element in MRSA strains^55^, are not regulated by *teg58* in biofilm cells as disclosed by qRT-PCR and enzymatic analysis in biofilm cells. Although various assays such as qRT-PCR, enzymatic and LC–MS/MS determinations for l-citrulline, and l-ornithine from aerobic grown cells in TSB revealed higher expression of some catabolic gene products, these results implied differential regulation of l-arginine metabolic activities between biofilm and aerobic grown cells in the *teg58* mutant. We believed that higher production of l-arginine in *teg58* mutant leads to higher activities of arginine metabolic pathway genes under aerobic growth condition. Interestingly, arginine permease (*lysP*) was found to be downregulated (~ fourfold) while *arcD* (antiporter) was unaltered in biofilm (Fig. 8A) but increased 2.0-fold in aerobically grown (Fig. 8B) cells of the *teg58* mutant, likely to minimize import of arginine while arginine can be synthesized to meet biological need under biofilm-growing condition.

We surmise that increased arginine production in the *teg58* mutant likely neutralizes acidic pH arising from glucose metabolism, in part to reduce the need to hydrolyze urea (by ureases) to form ammonia to elevate both the pH_in_ and the pH_out_. We are further investigating the growth phenotype of *teg58* mutant that can grow at acidic pHs (~ 5.0–5.5) compared to the wild type/complemented strains, suggesting that possibly increased intracellular l-arginine concentration likely enables the cells to sustain the growth of the *teg58* mutant under acidic pH condition. The acid tolerance of the *teg58* mutant with respect to growth is of clinical interest because neutrophils are formidable defender based in part on its ability to lower pH, generation of ROS, proteases, and host defense peptides inside the phagolysosomes. Therefore, it will be interesting to find out the exact role of *teg58* at acidic pHs and the role ureases play under acidic pH conditions.

Arginine has been reported to inhibit biofilm formation in several oral bacterial species by reducing insoluble extracellular polysaccharide production, resulting in a fragile biofilm architecture^33,34^. Biofilm assays (Fig. 5 and Fig. S4) demonstrated that biofilm formation can be inhibited by exogenously l-arginine or endogenous overexpression of arginine biosynthesis genes, a new finding in *S. aureus.* However, the exact manner by which arginine impacts biofilm formation in *S. aureus* is not clearly defined. We acknowledged that discrepancy in biofilm formation ability for the double mutants (*argG* or *H*) of *teg58* compared to the single mutant *argG* or *H* that could be due to other factors such as *relA* (Table 1) involved in biofilm dispersion^49,50^ or l-arginine mediated gene products, which yet to be identified in *S. aureus*. Our preliminary results indicated that the transcript level of *relA* by qRT-PCR and (p)ppGpp production determined by metabolomic are three–fivefold more in *teg58* mutants than the wild type and biofilm formation is up in *relA*_syn_ mutant. We are actively investigating the role of *relA* in *S. aureus* biofilm formation as well as in *teg58* mutant in combination with *argGH* system. Several metabolic transcriptional regulators such as CcpA, PurR, CodY and SrrAB are known to monitor metabolite levels and respond by directly modulating the transcription of genes utilized for central metabolism, energy generation and pathogenesis^56^, but expression of these genes was not significantly altered in the *teg58* mutant (Table S4). It remains unclear whether other modes of regulation monitor arginine level via the *teg58* pathway.

**Figure 9:**
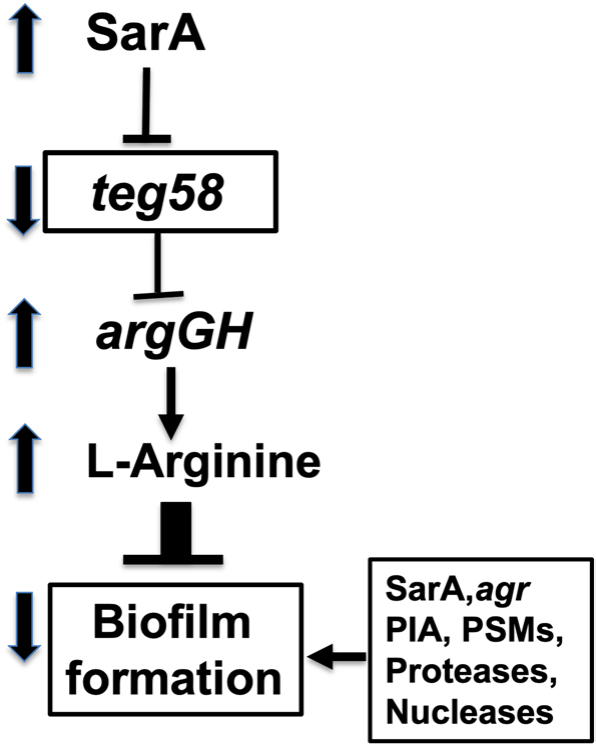
Proposed *teg58*-mediated pathway in biofilm formation via arginine production in *S. aureus*. SarA repressed *teg58*, a sRNA that represses *argGH* for l-arginine synthesis and production. *teg58* mediates biofilm formation independent of SarA, *agr, ica* (PIA), *psms* (PSMs), proteases (*aur, sspA, sspB, splA-F*) and nucleases (*nuc* and *nuc2*).

Overall, our results indicated that *teg58*, a sRNA repressed by SarA, is involved in biofilm formation in a process that potentially involves arginine production at the post-transcriptional level via *teg58*-*argGH* mRNA interaction (Fig. 9). We have also demonstrated that the catabolic pathway genes for arginine are not involved in *teg58*-mediated biofilm formation. Notably, RNA-Seq analysis revealed several up-regulated genes in the purine metabolism pathway in the *teg58* mutant vs. the parent (Table1). Interestingly, we have found increased amount of stringent molecule, (p)ppGpp involved in biofilm dispersal^57^, in the *teg58* mutant, which is under investigation. Whether combination of l-arginine and (p)ppGpp synthesis are responsible for decreased biofilm formation in the *teg58* mutant remains to be defined.

## Materials and methods

### Bacterial strains, plasmids, and primers

All strains, plasmids, and primers used in this study are listed in the Supplementary Tables S1and S2. *S. aureus* strains were grown in Tryptic Soy Broth (TSB; Benton Dickinson, NJ) or chemically defined medium (CDM) containing 1% glucose^42^ or on TSB agar or CYGP supplemented with antibiotics when necessary (5 μg/ml of erythromycin, and 10 μg/ml of chloramphenicol). Cultures were grown aerobically in a 1:5 flask-to-volume ratio, with shaking at 250 rpm at 37 °C. Luria–Bertani (LB) broth or agar was used for growing *Escherichia coli* supplemented with suitable antibiotics when necessary (100 μg/ml of ampicillin).

### Genetic manipulations in *E. coli* and *S. aureus*

All primers used for cloning, PCR, qRT-PCR, mutant construction, and plasmid construction can be found in Supplementary Table S2. Construction of mutant strains was performed using pMAD vector^58^ as described previously^23,24^. Briefly, to construct a *teg58* deletion mutant, the *teg58* sRNA region together with flanking sequences on both sides was amplified by PCR with primer pair *teg58* F and R to yield a 2.3 kb region flanking with *BamHI* sites (Table S2), using chromosomal DNA from strain SH1000 as a template. The 2.3 kb PCR fragment was digested and cloned into the cloning vector pUC19 at the *BamHI* site in *E. coli*. A 220-bp fragment comprising *teg58* was deleted by PCR with primer pair *teg58* ko F and R (Table S2). The fragment containing *teg58* deletion was excised with *BamHI* and cloned into the temperature-sensitive shuttle vector pMAD in *E. coli* IM08B. Plasmid isolated from IM08B^59^ was authenticated by digestion and sequencing and then introduced into JE2 and SH1000 strains by electroporation. Transformants were selected at 30 °C on erythromycin and X-gal (5-bromo-4-chloro-3-indoxyl-beta-d-galactopyranoside) containing plates. Mutant construction and confirmation were performed as described previously^23,24^. Utilizing the same vector (pMAD) and method, we constructed in-frame deletion mutants of the *arcA, rocF, argF*, and *ureA* genes in JE2. The double mutants of these genes with sRNA, *teg58*, were constructed using JE2△*teg58* (ALC8715) and the respective gene deletion in pMAD construct. The nitric oxide synthase (*nos*) mutants in JE2 and JE2△*teg58* (ALC8715) were constructed by phage transduction from the lysate of NE library strain NE27^11^ and confirmed by chromosomal PCR and DNA sequencing.

To complement the *teg58* deletion strains, a 450-bp fragment encompassing the *teg58* sRNA with 170-bp upstream and 101-bp downstream was cloned into shuttle plasmid pSK236^43^ in *E. coli* IM08B strain^59^. The construct was verified by plasmid restriction analysis and DNA sequencing, then electroporated into parental and isogenic *teg58* mutant strains. To create the transcriptional fusion, a 210-bp *teg58* promoter or 202-bp *argGH* promoter fragment was amplified by PCR using chromosomal DNA of *S. aureus* strain JE2 and primers with flanking *EcoRI* or *XbaI* sites and cloned into shuttle plasmid pALC1484^44^ to generate the transcriptional fusion of the *teg58* or *argGH* promoter to the *gfp*_*uvr*_ reporter gene. The effect of endogenous arginine on biofilm formation was evaluated with an anhydrotetracycline-inducible promoter system to yield a gradient of *argGH* expression. The polycistronic *argGH* coding region, together with the upstream ribosomal binding site, was ligated downstream of the *xylR/tetO* promoter in pALC2073 containing the *tetR* repressor^51^.

#### Isolation of total cellular RNA, northern blot hybridization

Isolation of total cellular RNA from various growth phases or biofilm cells and subsequent analysis by Northern blot hybridization were performed as described previously^24^. Biofilm cells were scrapped from the wells of 96-well plate and pooled before harvesting. For sRNA, 15 μg each of total RNAs were separated onto 8% polyacrylamide/8 M urea gels and transferred them onto Hybond-N + membranes (Amersham, USA). For mRNA, 10 μg each of total RNAs were separated onto 1% agarose-formaldehyde gels and transferred them onto membranes. DNA fragments encoding the open reading frames of the *sarA*, *agrA, agr* RNAIII, *icaA, icaR, argG, argH,* sRNA *teg58, Tm RNA,* and other genes were amplified by PCR or excised from the plasmids containing the desired genes with restriction endonucleases and then gel purified. For detection of specific transcripts, gel-purified DNA probes were radiolabeled with [α-^32^P]-dCTP by using the random-primed DNA labeling kit (Roche Diagnostics GmbH) and hybridized under aqueous-phase conditions at 65 °C. The blots were subsequently washed, exposed for overnight to PhosphoImager screen and scanned. Each of the experiments was repeated at least three times independently.

#### Chromosome immunoprecipitation (ChIP) assay

ChIP experiment was performed as described^60^. Detailed method and analysis of ChIP-Seq were described in our previous publication^40^.

#### Primer extension and 3′ RACE assays

Mapping of the 5′-end of *teg58* sRNA transcript by primer extension was performed using the primer 5´-ACAATAGCCACCTCGCTTGTTCA-3′, complementary to the *teg58* RNA strand and located from nt positions 112 to 90 downstream from the mapped putative transcriptional start site. Primer extension and 3´ RACE assays to map the 5´- and 3´- end of *teg58* sRNA transcript, total cellular RNA was isolated from the wild type and *sarA* mutant strains, purified, and assays were performed as described previously^23^. Reverse transcription was carried out as described elsewhere by using total RNA isolated from wild-type SH1000 or the *sarA* mutant as described previously^24^. Finally, 3′ RACE-PCR products were sequenced, and sequences were aligned with *teg58* region of the NCTC8325 annotated genome sequence.

### qRT-PCR analysis

For real-time qRT-PCR experiments, strains SH1000 or JE2, isogenic *teg58* mutant, and *teg58* complemented *teg58* mutant were grown in TSB media to post-exponential phase of growth (~ OD_600nm_ of 1.4). Total RNA was isolated from planktonic, or biofilm cells as described earlier. Quantitative real-time PCR (qRT-PCR) was performed as previously described^24^. Briefly, 5 μg of isolated RNA was treated with Turbo DNaseI, purified, and 500 ng was used as a template for making cDNA by using RevertAid H minus first strand cDNA synthesis kit (Thermo Scientific, USA). SsoFast EvaGreen supermix (Bio-Rad, USA) was used to generate standard curves for the cDNA concentration/crossing point (C_p_) for the target genes and the reference gene, *rpoB*. The mean target transcriptional expression level for the three transcript measurements was calculated. The threshold cycle (2^−ΔΔCT^) method was used to calculate relative changes in gene expression, using triplicate samples, Bio-Rad’s CFX Maestro software was used to analyze the qRT-PCRs. Control reaction mixtures containing master mix and primers, but no cDNA was also analyzed.

### RNA-Seq library preparation and analysis

Samples for transcriptome sequencing (RNA-Seq) were prepared from the following strains, the wild type SH1000, and its isogenic derivative *teg58* mutant (ALC8717) and the isogenic *teg58* mutant complemented *teg58* strain (ALC8749). Briefly, overnight cultures were diluted 1:100 in fresh TSB (an initial OD_600_ of about 0.04–0.05) and grown at 37 °C for 4 h. Two replicate cultures for each sample were grown, and total cellular RNAs from two biological replicates were extracted as described previously^24^. RNA was quantified and 1 µg of total RNA was ribo-depleted with the bacterial Ribo-Zero kit from Illumina. The truseq total RNA stranded kit from Illumina was used for the library preparation, and quantity and quality were measured/assessed. The libraries were pooled at equimolarity and loaded at 2 nM for clustering. Oriented 50 bases single-read sequencing was performed on the Illumina HiSeq 4000 sequencer yielding a minimum of 50 million mapped reads per sample. Final RNA-Seq analysis and data analysis were carried out using previous described procedures^24^. Statistical analyses were done in R v3.2.3 using the edgeR package^47^ following the bioinformatics protocol described^61^.

### Biofilm assay

Quantification of biofilm formation on abiotic surfaces was performed as described previously^24^. Briefly, *S. aureus* strains grown overnight in TSB were diluted (1:50) in TSB supplemented with 0.25% glucose or CDM containing 1% glucose and l-arginine (0.5 mM). This cell suspension was used to inoculate sterile 96-well “U” bottom polystyrene microtiter plates (Costar, Corning Inc., USA) in triplicate. After 24 h of incubation at 37 °C, wells were gently washed three times, air dried, and stained with 0.1% crystal violet dye. Wells were rinsed 3–4 times, dried for overnight, solubilized in 30% acetic acid, and solubilized dye was transferred to a fresh plate and read at 550 nm (Tecan Infinite M1000 Pro, Austria). Each assay was repeated at least three times in separate experiments.

### PIA detection

PIA production in *S. aureus* was detected by Dot blot as described by Cramton et al.^62^. Briefly, cells were grown overnight in TSB supplemented with 0.25% glucose, the optical density was determined, and the same number of cells from each culture was resuspended in 50 μl of 0.5 M EDTA (pH 8.0). Cells were incubated for 7 min at 100 °C and centrifuged to pellet the cells, and the supernatant was incubated with 10 μl of proteinase K (10 mg/ml) for 30 min at 37 °C. After incubation, 10 ul of Tris-buffered saline (pH 7.5) containing 0.01% bromophenol blue was added, and 5 μl was spotted on a nitrocellulose membrane, dried, blocked with 3% bovine serum albumin for 2 h., and incubated overnight with an anti-*S. aureus* PIA antibody (1:250) (gifted from Dr. M. Otto, NIH). Bound antibodies were detected with a secondary alkaline phosphatase conjugated anti-rabbit immunoglobin G (IgG) antibody (Jackson Laboratory) diluted 1:5000. Finally, blot was detected in developing solution.

### Butanol extraction of PSMs

A comparative PSMs analysis for various strains was performed by extracting PSMs from various *S. aureus* culture supernatant using previously described procedure^46^. Briefly, various *S. aureus* strains were grown for biofilm formation in plastic plates in TSB medium containing 0.25% additional glucose for 24 h, scrapped from the wells, the OD_650nm_ measured and centrifuged to obtain the culture supernatants which were then sterile filtered to remove any cells. One third (v/v) of 100% n-butanol was added to the spent supernatants, shaken vigorously at 37 °C for 2 h, and centrifuged. The upper (butanol) layer was removed and dried by vacuum centrifugation. To visualize PSMs, extracts were dissolved in TE buffer (10 mM Tris–Cl pH7.6 and 1 mM EDTA), mixed with 6 X SDS PAGE gel loading buffer, separated on 12% PAGE-SDS gels, and stained with Coomassie brilliant blue.

### DNA and RNA electrophoretic mobility shift assay

Gel shift experiments for the binding of SarA to the radiolabeled promoter fragment (210 bp) of *teg58* was performed as previously described^5,59^. The sRNA-mRNA interaction was performed according to the described procedures by Morita et al.^52^. Briefly, ~ 2.6 kb long *argGH* coding region was divided into five different overlapping fragments (1–600 bp, 350–950 bp, 875–1475 bp, 1000–1625 bp, and 1500–2200 bp). These DNA fragments and *teg58* were amplified by PCR using upstream primer containing T7 promoter sequence and purified using GeneJET PCR purification kit (Thermo Fisher Scientific, USA). RNA was synthesized using HiScribe T7 In Vitro Transcription kit (NEB Biolabs, USA) according to the manufacturer’s protocol, treated with RNase-free DNase I and purified using GeneJET RNA purification kit (Thermo Fisher Scientific, USA). Ten pmol of various synthesized *teg58* sRNAs were dephosphorylated with alkaline phosphatase, purified, end-labeled with γ^32^P ATP, purified with a 6% polyacrylamide—6 M urea gel and eluted with elution buffer (0.5 M ammonium acetate, 10 mM magnesium acetate, 1 mM EDTA, 0.1% SDS) at 4 °C for overnight followed by purification and ethanol precipitation. The pellets were dissolved in sterile RNA grade water to a 0.1 pmol per μl. 0.2 pmol of ^32^P-labeled *teg58* RNA was mixed with 0.4 pmol of various truncated *argGH* mRNAs in 20 μl of binding buffer (20 mM Tris–Cl pH8.0, 1 mM MgCl_2_, 20 mM KCl, 1.0 mM Na_2_HPO_4_–NAH_2_PO_4_, pH8.0) and incubated at 70 °C (or 37 °C) for 5 min. After heating, the samples were slowly cooled to 30 °C, mixed with 0.2 vol of loading buffer, loaded onto a 4% polyacrylamide native gel in 0.5 × TBE containing 5% glycerol and ran at 4 °C until the bromophenol blue migrates to 2/3 of the gel. The gel was vacuum dried on Whatman paper, exposed to PhosphorImager screen, and developed.

### Western blotting and immunodetection

Whole cell extracts from *S. aureus* wild types or various mutant strains were prepared from cells grown to different growth phases as described previously^24^. The concentration of total proteins from clear lysates was determined by using the Bio-Rad BCA protein assay dye using bovine serum albumin as the standard. Western blotting and detection with primary antibody (anti-SarA) and goat anti-mouse secondary antibody conjugated with alkaline phosphatase and 1-Step NBT/BCIP blotting substrate (Thermo Scientific) were performed as described previously^24^.

### RNA stability assay by real-time qRT-PCR

For RNA stability experiments, strains SH1000 or JE2, isogenic *teg58* mutant, and *teg58* complemented *teg58* mutant were grown in TSB media to an OD_600_ of 1.2 (mid-log phase of growth). After the addition of rifampin to 200 μg per ml, samples were collected between 0- and 30-min. Total RNA was isolated as described earlier and quantified by measuring the absorbance at 260 nm. 5 μg of isolated RNA was treated with Turbo DNaseI, purified, and 500 ng was used as a template for making cDNA by using RevertAid H minus first strand cDNA synthesis kit (Thermo Scientific, USA). qRT-PCR was performed as described earlier in qRT-PCR section using *argG* specific primers.

### Measurement of the enzymatic activities of the arginine catabolic pathway by determining l-citrulline concentrations

The l-citrulline concentration was determined by a colorimetric 96-well microtiter plate assay as described^54^. Briefly, various cells were grown in shaking tubes or biofilm formation growth in 96-well plastic microtiter plate for 24 h. Biofilm cells were scrapped, pooled, centrifuged, and washed once with 0.1 M sodium phosphate buffer (pH 6.2). The cell pellets were resuspended in 0.5 ml of 0.1 M phosphate lysis buffer (25 mM Tris-pH7.0, 150 mM NaCl, and 0.1 mM EDTA) and lysed in combination with 0.1-mm-diameter zirconia-silica beads in a Fast Prep reciprocating shaker. The concentration of the lysate proteins was determined with the Bio-Rad protein assay solution (Bio-Rad, USA), using bovine serum albumin as the standard.

The calibration curve was obtained using l-citrulline (L7629; Sigma-Aldrich, USA) concentrations between 0 and 400 μM in a volume of 60 μl in a 96-well polystyrene microtiter plate (Costar RIA/EIA clear flat bottom plate) in triplicates. A color developing reagent (COLDER) solution was freshly prepared by mixing 1 vol of solution A [80 mM DAMO (diacetyl monoxime, B0753, Sigma-Aldrich) and 2.0 mM thiosemicarbazide, T33405, Sigma-Aldrich] and 3 vol of solution B [3.0 M H_3_PO_4_, 6.0 M H_2_SO_4_, and 2 mM NH_4_Fe(SO_4_)] and storing in the dark before use within 1 h. Then 200 μl of COLDER was added to each well and a microseal “B” adhesive seal (Bio-Rad, USA) was used for plate closure. The plate was placed onto the bottom of a preheated aluminum block, covered with a preheated glass plate, and incubated in an oven at 95 °C for 15 min. After cooling down for 10 min, the sample absorptions were measured in a plate reader (Tecan Infinite M1000 Pro, Austria) at 540 nm. The values were plotted against the concentrations of l-citrulline to obtain a standard curve.

Measurement of the arginine catabolic pathway enzymatic activities lead to l-citrulline production under different conditions of various strains was performed using fixed amount (50 or 100 μg) of various cell lysates. Fixed amount of cell lysate was added to the well in triplicates and the volume was adjusted to 55 μl with 0.1 M sodium phosphate buffer (pH 6.2). The enzymatic reaction was initiated with addition of 5 μl of 80 mM MMA (monomethylated l-arg, M7033, Sigma-Aldrich, USA), a substrate, and incubated for 30 min at 37 °C in a water bath. The reaction was stopped by the addition of 200 μl COLDER solution and the concentration of l-citrulline as determined as described above. One unit is defined as the amount of enzyme that produces 1 μM of l-citrulline per minute under the experimental conditions. The enzymatic activities for various strains were calculated based on the l-citrulline standard curve. Each assay was repeated at least three times in separate experiments.

### Determination of l-arginine by UHPLC-HRMS(/MS)

The wild type and *teg58* mutant of JE2 were grown under aerobic conditions. Cells were collected by centrifugation and washed with ice-cold 150 mM ammonium acetate before adding 1 ml of ice-cold MeOH:H_2_O 80:20 (v/v) onto the pellet for metabolism quenching and cell content extraction. Cells were lysed by a bead beater (FastPrep) and supernatants were separated from pellets by centrifugation, collected into fresh tubes and evaporated. Dry extracts were redissolved in H_2_O/200 mM NH_4_OAc pH 9.05/MeCN 30:10:60 (v/v/v) prior to LC–MS analysis. Chromatographic separation was performed on an Acquity I-Class UHPLC system (Waters, Milford, USA), equipped with a flow-through needle (FTN) autosampler, heated column compartment and binary solvent delivery system with a delay volume of 80 μl. The samples were injected onto Acquity™ BEH Z-HILIC (2.1 × 150 mm, 1.7 µm, 95 Å) column and temperature of the column compartment was maintained at 30 °C. Gradient elution was performed in 35 min. On-line coupled QExactive™ Focus mass spectrometer (Thermo Fisher Scientific, USA), featuring heated electrospray ionization (HESI II) ion source, orbitrap mass analyzer with mass selection quadrupole and higher energy dissociation (HCD) collision cell, supplied with N_2_ as a collision gas had been used for acquisition of the MS/MS data. Mass accuracy was calibrated below 2 ppm using Pierce LTQ Velos calibration solution according to the requirements of the manufacturer. All operations were controlled by Xcalibur software supplied by vendor. MS/MS experiments were performed in top3-DDA mode with an absolute collision energy (CE) stepping of 10/20/50 eV at R = 35,000 (m/z 200), 1 microscan, fixed injection time of 90 ms (30 ms/step) and AGC target of 2e5 charges. Quadrupole isolation width was set to 1 Da, isolation threshold of 1e4 and precursor exclusion for 3 s were applied. Survey scan parameters for top3-DDA experiment were identical to those used in full scan measurements except the number of microscans, which was set to 1. Reference spectra were obtained from an injection of 1 ppm solution (ACN/H_2_O, 3:1) of commercially available l-arginine (Sigma-Aldrich) or other metabolites.

### Software analysis

All statistical analysis and graph production were done using GraphPad Prism 9 and Microsoft excel programs. All data were obtained from at least three independent experiments with multiple replicates unless stated otherwise. Student’s t-test was applied to analyze data on CFU, biofilm assays, and qRT-PCR. A p-value of < 0.05 was considered as statistically significant. Scanned images from non-saturated exposed PhosphorImager were analyzed densitometrically using ImageJ 1.51 g (NIH). Cropping of the original files were performed using Image J and Adobe Photoshop CS6 programs.

## Supplementary Information

Supplementary Information 1.

Supplementary Information 2.

Supplementary Information 3.

## Data Availability

The datasets generated during and/or analyzed during the current study are available from the corresponding author upon reasonable request. Raw RNA sequencing data have been deposited at ENA (https://www.ebi.ac.uk/ena) under the following name: PRJEB48126.
