# Supplementary material for: Teg58, a small regulatory RNA, is involved in regulating arginine biosynthesis and biofilm formation in Staphylococcus aureus
Source: Sci Rep. 2022 Sep 2;12:14963. doi: 10.1038/s41598-022-18815-3 (PMC9440087; doi:10.1038/s41598-022-18815-3)
Supplement: Supplementary file 1 — Supplementary Information 1. [file 41598_2022_18815_MOESM1_ESM.pdf]

SUPPLEMENTARY INFORMATION FILES:

Teg58, a Small Regulatory RNA, is Involved in regulating Arginine Biosynthesis and Biofilm Formation in *Staphylococcus aureus*

Adhar C Manna[1]*, Stefano Leo[2], Sergey Girel[3,4], Víctor González-Ruiz[3,4], Serge Rudaz[3,4], Patrice Francois[2], and Ambrose L. Cheung[1]

[1] Department of Microbiology & Immunology, Geisel School of Medicine at Dartmouth, Hanover, New Hampshire 03755, USA

[2] Genomic Research Laboratory, Service of Infectious Diseases, Geneva University Hospitals and University Medical Center, Rue Michel-Servet 1 CH-1211 Geneva 4, Switzerland

[3]Institute of Pharmaceutical Sciences of Western Switzerland (ISPSO), [4]School of Pharmaceutical Sciences, University of Geneva, Geneva, Switzerland. University Medical Center, Rue Michel-Servet 1, 1211 Geneva 4, Switzerland

Running title: sRNA *teg58* involve in biofilm formation

*Address correspondence to Adhar C. Manna, Adhar.C.Manna@Dartmouth.edu.

SUPPLEMENTAL Figures S1-5, and Tables S1 and S2:

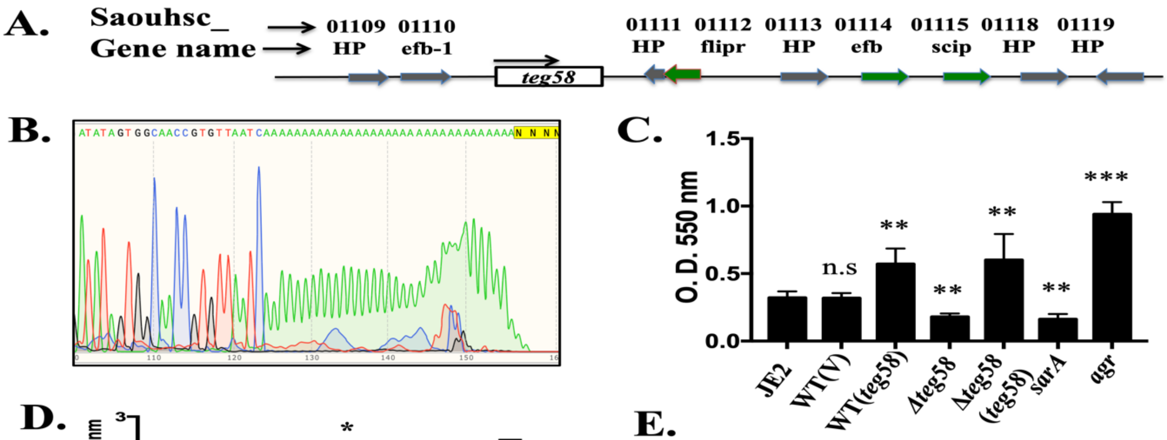

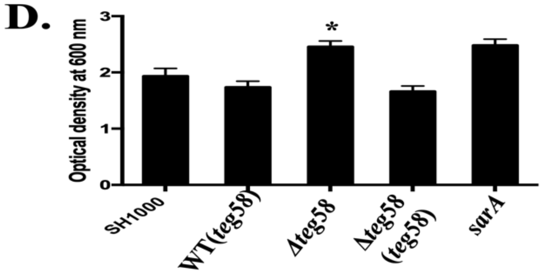

E.

Strain	% from Input cells, 1 h	% from input cells, 2h
JE2	885%	1142%
Δ*teg58*	53%	30%
Δ*teg58* (*teg58*)	1222%	1335%

Figure S1. Organization of various genes in the *teg58* region and biofilm formation mediated by *teg58*. A. Genetic cluster near *teg58* sRNA containing several virulence factors. HP, hypothetical protein; *efb-1,* fibrinogen-binding protein–like protein; *flipr*, formyl peptide receptor-like inhibitory protein; *efb*, fibrinogen binding protein; *scip*, staphylococcal complement inhibitory protein. **B**. 3´-RACE DNA sequencing reactions indicating the 3´ -end of *teg58*. **C.** Static biofilm assay with isogenic strains of JE2 in TSB with 0.25% glucose for 24 hours. The *sarA* and *agr* mutant strains are used as controls. WT (V) or WT(*teg58*), WT harboring pSK236 vector or containing 450-bp *teg58* with its native promoter. Δ*teg58*(*teg58*), *teg58* mutant complemented with pSK236 containing 450-bp *teg58* with its native promoter. **D.** Measured optical density or cells density of the planktonic biofilm cells from the static biofilm grown wells for various strains. **E.** Table showing the attached biofilm cells that were scrapped from the biofilm wells of panel **C,** plated, and CFUs were counted. Data were presented as percentage cells attached from the initial input CFUs. The asterisks in figure indicate statistical significance between wild-type and isogenic mutant or complement strains, determined using Student *t*-test (*, $P < 0.05$; **, $P < 0.005$; *** or ****, P <0.0001) or "n.s" indicates "not significant".

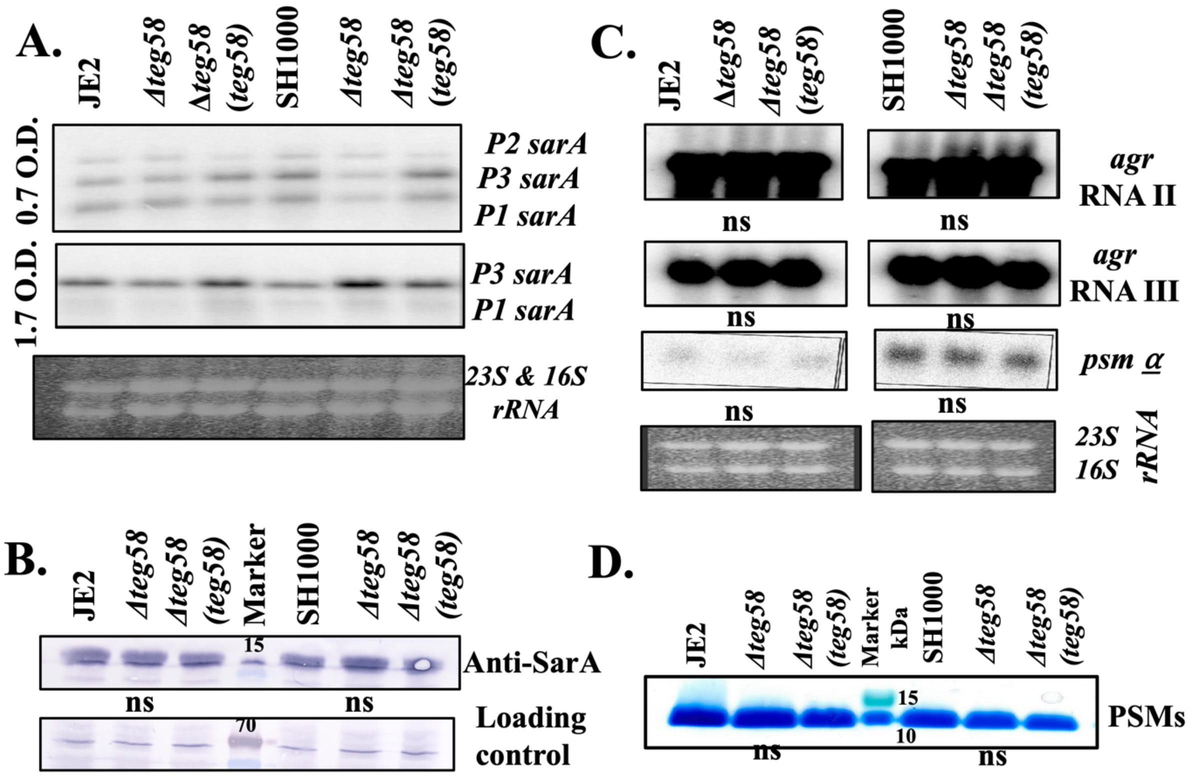

Figure S2. ***Teg58*** **phenotype is independent of SarA and** ***agr*** **systems.**

A. Transcription analysis of various *teg58* strains with a *sarA* probe at different phases of growth as indicated and cultures were grown under biofilm formation conditions. In all lanes, 10 µg of cellular RNA from the post exponential phase of growth was loaded onto each lane. The 23S and16S rRNA of an ethidium bromide-stained gel used for blotting are shown as the loading control. Δ*teg58*, 220-bp chromosomal deletion strain; Δ*teg58*(*teg58*), complemented with pSK236 with 450-bp *teg58* and its native promoter. Original blots are presented in Supplementary Material files Figure X6A. **B.** Western blot analysis of the same cultures as in panel A with anti-SarA monoclonal antibody. 10 µg of total cell-free lysate was loaded in each lane and non-specific bands (bottom panel) of the same blot showed as loading control. Original blots are presented in Supplementary Material Files Figure X6B. **C.** Transcript analysis of various *teg58* derivative strains with *agr* RNAII *agr* RNAIII, and *psm-α* genes. Total cellular RNA was isolated from biofilm cultures by scrapping cells from the wells. The relative band intensity of various lanes was determined by Image J software (NIH) and the statistically significant was calculated considering the wild type as 100. Original blots are presented in Supplementary Material Files Figure X6C and D. **D.** A comparative analysis of a Coomassie stained SDS-PAGE analysis of n-butanol extracts of the supernatant of various scrapped cells from biofilm formed wells as described in Methods and Materials. The same bands were also excised from gel and analyzed by Mass Spectroscopy to determine the identities of different peptides. Original blot is presented in Supplementary Material Files Figure X6E.

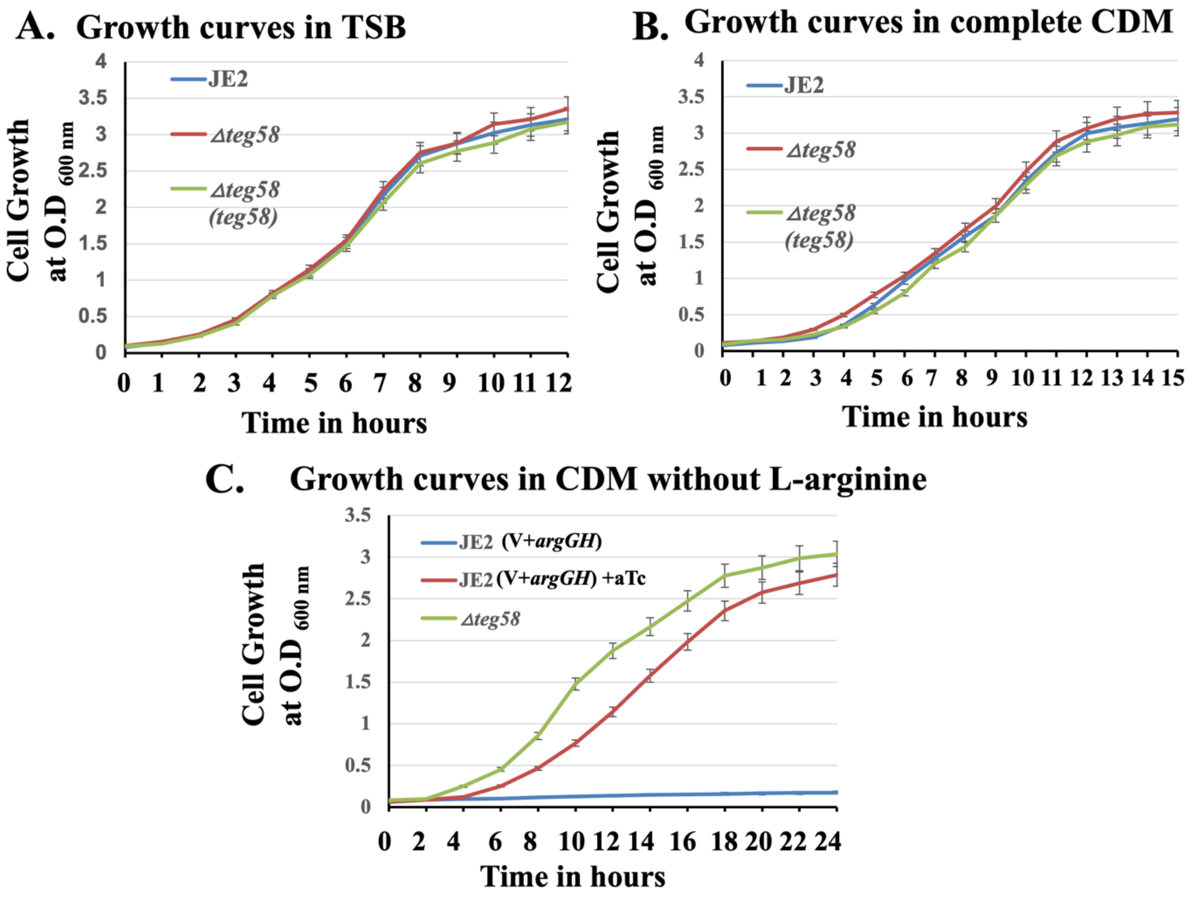

Figure S3. Growth analysis of the wild type, *teg58* mutant, and *trans*-complemented *teg58* mutant in various media. A. and B. Growth curves for various strains in TSB (A), and complete CDM (B). There was no growth defect in both tested media. **C.** Growth analysis of various strains in CDM without L-arginine. JE2 (V+*argGH*), indicates the wild type JE2 with pALC2073(V) with *argGH* without induction with anhydrotetracycline (aTc), JE2 (V+*argGH*) +aTc, indicates the wild type JE2 with pALC2073(V) with *argGH* with induction with 500 ng/ml anhydrotetracycline (aTc), and Δ*teg58*, an isogenic *teg58* mutant. Optical density at 600 nm was measured in Eppendorf Biophotometer plus.

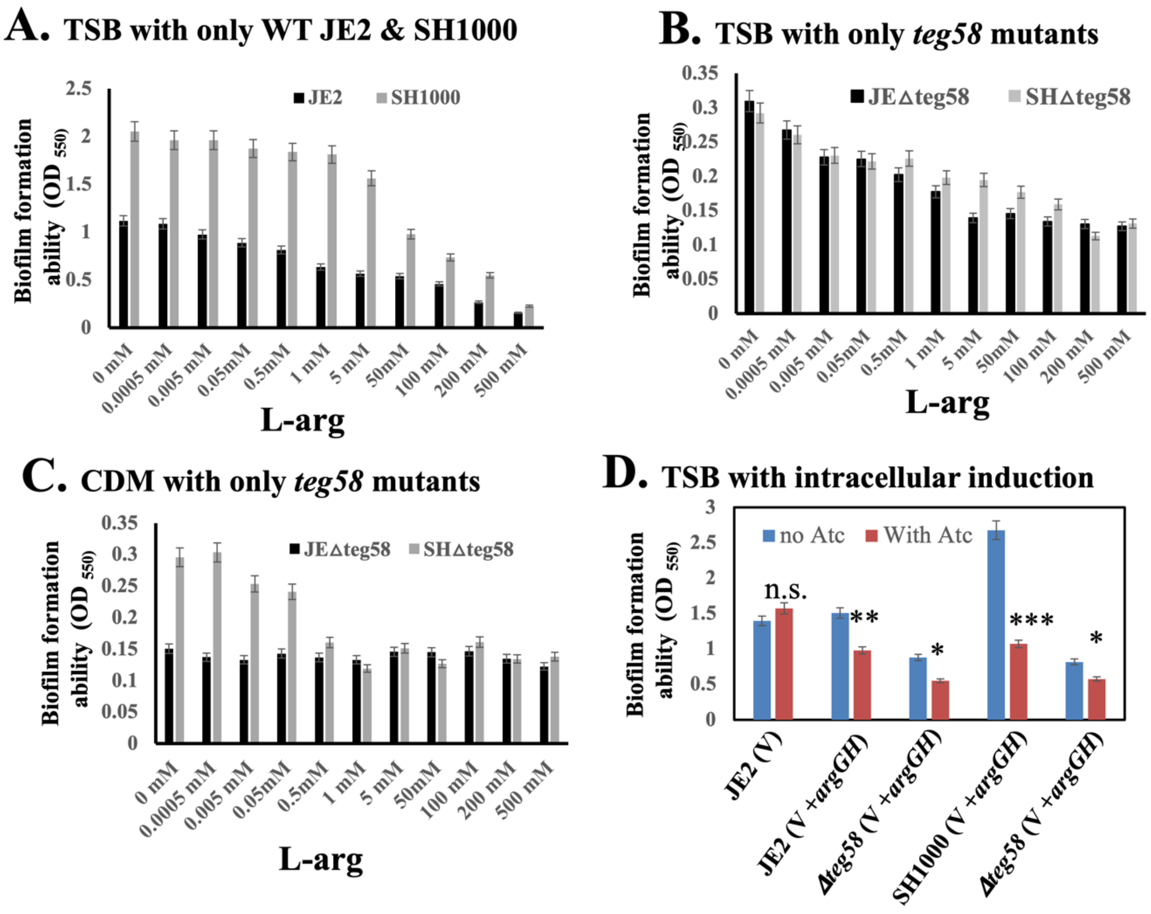

Figure S4. Inhibition of biofilm formation in various strains.

A. Biofilm formation for various wild type strains in TSB with added 0.25% glucose and various concentrations of exogenous L-arginine for 24 hours in a microtiter plate. **B** and **C.** Biofilm formation for various Teg58 mutant strains as indicated in TSB plus 0.25% glucose (**B**) and complete CDM (**C**) containing 1% glucose and various concentrations of exogenous L-arginine for 24 hours in a microtiter plate under different conditions. **D.** Biofilm formation for various strains as indicated in TSB with added 0.25% glucose for 24 hours in a microtiter plate under different conditions. V represents pALC2073 as the vector control; V+*argGH*, represents pALC2073 with *argGH* including ribosome binding site cloned downstream of *tetO*-inducible promoter; Atc (anhydrotetracycline) is the inducer for pALC2073. The asterisks in figure indicate statistical significance between uninduced and induced conditions, determined using Student *t*-test (*, $P < 0.05$; **, $P < 0.005$; ***, P <0.0001) or "n.s" indicates "not significant".

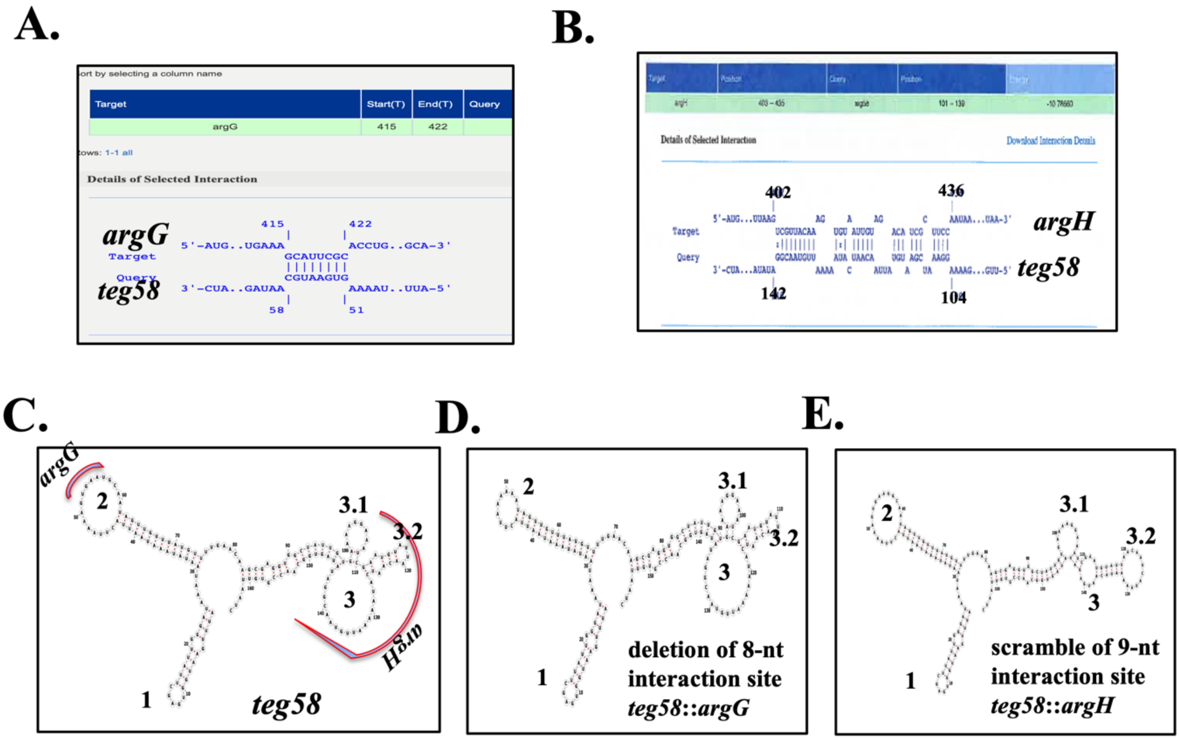

Figure S5. Proposed models for the interactions between *teg58* and the *argG* and *argH* genes. The region predicted to interact with *teg58* with *argG* mRNA (**A**) and *argH* mRNA (**B**) as determined by Freiburg IntaRNA Tools (rna.informatik.uni-freiburg.de).

C-E. Predicted secondary structure of *teg58* (**C**) and the mutated *teg58* as indicated using RNAfold webserver [1]. Sites of interaction are indicated with red signs in **C**. Three major folding hairpins are marked as 1,2, and 3, while two minors are the part of hairpin 3 marked as 3.1 and 3.2. Complete retention of the wild type of structure in D due to 8-nt deletion of *argG* and *teg58* interaction site, while some rearrangement of the hairpin 3 structure due to scrambling of 9-nt in the *argH* and *teg58* interaction region.

TABLE S1. **Bacterial strains, and plasmids used in this study**

Strain or plasmid	Description	Reference or Source
S. aureus		
JE2	MRSA JE2, USA300_FPR3757, a USA300 LAC derivative cured of its plasmids	2
SH1000	Wild type laboratory strain with a functional *rsbU*$^{+}$ derivative of 8325-4 *rsbU* defective strain	3
ALC8715	*teg58* mutant of JE2, chromosomal deletion of 220 bp containing 179 bp *teg58* sRNA along with 26 bp upstream and 15 bp downstream region.	This study
ALC8693	HG003 *sarA::ermC* (pSK236 P1 *sarA-myc*)	4
ALC8717	*teg58* mutant of SH1000, chromosomal deletion of 220 bp containing 179 bp *teg58* sRNA along with 26 bp upstream and 15 bp downstream region	This study
ALC8748	ALC8715 with pSK236 containing 450-bp *teg58* fragment DNA with its native promoter for *trans*-complementation.	This study
ALC8749	ALC8717 with pSK236 containing 450-bp *teg58* fragment DNA with its native promoter for *trans*-complementation.	This study
ALC 2732	*sarA* mutant of SH1000 *sarA::ermC*	5
AM 1262	Complemented with *sarA* into *geh* locus on chromosome of *sarA* mutant of SH1000 *sarA::ermC*	5
ALC8644	*sarA* mutant of JE2 *sarA::ermC*	This study
ALC8445	Complemented with *sarA* into *geh* locus on chromosome of *sarA* mutant of JE2 *sarA::ermC*	This study
ALC7076	SH1000 Δa*grA*, markerless deletion mutant of the *agrA* gene	This study
ALC7075	SH1000 Δ*sarA*, markerless deletion mutant of the *sarA* gene	This study
ALC8994	JE2 *argG::ermC,* transduced from NE mutant library strain	This study
ALC8995	JE2 *argH ::ermC,* transduced from NE mutant library strain	This study
ALC9012	SH1000 *argG::ermC,* transduced from NE mutant library strain	This study
ALC9013	SH1000 *argH ::ermC,* transduced from NE mutant library strain	This study
ALC9045	JE2 *argH::ermC* Δ*teg58* double mutant	This study
ALC9046	JE2 *argG::ermC* Δ*teg58* double mutant	This study
ALC9048	SH1000 *argH::ermC* Δ*teg58* double mutant	This study
ALC9049	SH1000 *argG::ermC* Δ*teg58* double mutant	This study
ALC9074	ALC8715 with pSK236 containing 450-bp *teg58* fragment DNA with its native promoter with deletion of 8 bp interaction site between *argG* and *teg58*.	This study

ALC9123	ALC8715 with pSK236 containing 450-bp *teg58* fragment DNA with its native promoter with scramble 9 bp interaction site between *argH* and *teg58*.	This study
ALC9076	ALC8717 with pSK236 containing 450-bp *teg58* fragment DNA with its native promoter with deletion of 8 bp interaction site between *argG* and *teg58*.	This study
ALC9125	ALC8717 with pSK236 containing 450-bp *teg58* fragment DNA with its native promoter with scramble 9 bp interaction site between *argH* and *teg58*.	This study
ALC9159	JE2 chromosomal deletion of 9-bp or three codons of the *argG* gene (nt 415-423) at *teg58-argG* interaction site.	This study
ALC9161	JE2 chromosomal mutation by scrambling 9 bp or three codons of argH gene (nt 1598-1606) at the interaction site between *argH* and *teg58*.	This study
ALC8909	JE2 containing *tetO* inducible pALC2073 vector.	This study
ALC9172	JE2 containing pALC2073 with 2.7 kb *argGH* genes with its ribosome binding site.	This study
ALC9173	ALC8715 containing pALC2073 with 2.7 kb *argGH* genes with its ribosome binding site.	This study
ALC9174	SH1000 containing pALC2073 with 2.7 kb *argGH* genes with its ribosome binding site.	This study
ALC9175	ALC8717 containing pALC2073 with 2.7 kb *argGH* genes with its ribosome binding site.	This study
ALC9271	JE2 Δ*arcA*: chromosomal in frame deletion of the *arcA* gene	This study
ALC9270	JE2Δ*teg58* (ALC8715) Δ*arcA,* double mutant	This study
ALC9315	JE2 Δ*rocF*: chromosomal in frame deletion of the *rocF* gene	This study
ALC9316	JE2Δ*teg58* (ALC8715) Δ*rocF,* double mutant	This study
ALC9319	JE2 Δ*argF*: chromosomal in frame deletion of the *argF* gene	This study
ALC9320	JE2Δ*teg58* (ALC8715) Δ*argF,* double mutant	This study
ALC9317	JE2 Δ*ureA*: chromosomal in frame deletion of the *ureA* gene	This study
ALC9318	JE2Δ*teg58* (ALC8715) Δ*ureA,* double mutant	This study
ALC9327	JE2 *nos::ermC,* transduced from NE mutant library strain NE27	This study
ALC9328	JE2Δ*teg58* (ALC8715) *nos::ermC*, , transduced from NE mutant library strain NE27	This study
E. coli		
XL-1 Blue	General cloning strain	Agilent
IM08B	General shuttle dam methylation strain between *E. coli* and *S. aureus, dam*$^{+}$ *dcm*$^{-}$ *hsdRM*-2CC8, *recA*$^{-}$	6
Plasmids		
pMAD	*E. coli-S. aureus* shuttle vector containing temperature-sensitive origin of replication (pE194^{ts}), *bgaB* , Erm^{r} Ap^{r}	7
pSK236	Shuttle vector containing pUC19 cloned into the *Hin*dIII site of pC194, Chlor^{r}, Ap^{r}	8

pALC1484	pSK236 with a promoterless *gfp*$_{uvr}$, a cycle-3 *gfp* allele	9
pALC2073	Anhydrotetracycline inducible *tetO* shuttle vector	10
pALC8876	pALC1484 containing 210 bp *teg58* promoter region at *EcoRI*-*XbaI* sites as pALC1484 ::P*teg58*-*gfp*$_{uvr}$	This study
pALC8652	pMAD containing 2.3 kb *teg58* chromosomal region with 220 bp deletion of sRNA *teg58*.	This study
pALC9085	pALC1484 containing 202 bp *argGH* promoter region at *EcoRI*-*XbaI* sites as pALC1484 ::P$_{argGH}$-*gfp*$_{uvr.}$	This study
pALC8725	pSK236 containing 450 bp *teg58* region including its native promoter for *trans*-complementation.	This study
pALC9151	pMAD containing 2.6 kb with *argGH* region with deletion of 9-nt or three codons of the *argG* gene (nt 415-423) at *teg58*-*argG* interaction site.	This study
pALC9152	pMAD containing 2.6 kb with *argGH* region with scrambling 9 bp or three codons of *argH* gene (nt 1598-1606) at the interaction site between *argH* and *teg58*.	This study
pALC9068	pSK236 containing 450-bp *teg58* fragment DNA with its native promoter with deletion of 8 bp interaction site between *argG* and *teg58*.	This study
pALC9069	pSK236 containing 450-bp *teg58* fragment DNA with its native promoter with scramble 9 bp interaction site between *argH* and *teg58*.	This study

Table S2. **Primers used in this study**

Name	DNA sequences
teg58 2.3 kb region F	AGG AGA GGA TCC CAA TTT CAA AAA TCG ACA TCA CG
teg58 2.3 kb region R	AGA AGA GGA TCC CAA AAC AGC TAT ATG TAT AAC
teg58 ko F	AAC AGT CGT AAA GAT GCT AAA TAA ATT G
teg58 ko R	TCT ATA GCA TGG CTT CTA TCA ATT A
teg58 sRNA F	ATT GTG TTT ATT TGA ATG GTG AGC TTA
teg58 sRNA R	AGC ACG TAT GAT TAA CAC GGT TGC CA
teg58 Primer Extension	ACA ATA GCC ACC TCG CTT GTT CA
teg58 Promoter EcoRI	AAG GAA GAA TTC CAT GCT ATA GAG TGT GGC TTT
teg58 Promoter XbaI	AAG GAA TCT AGA CTT TTA AGT AAT TAT TTA TAT TAT TTA TC
Teg58 Complement F	AAAG GAC GAA TTC ATT TCC AAC ACA GTT ATG AAA
Teg58 Complement R	AGAA GAG GAA TTC GAT TAC TTG GTA TTA AAA AAC ATA CTG
Teg58 T7 promoter for *in vitro* RNA synthesis	AGATGTAATACGACTCACTATAGG TTG AAT GGT GAG CTT ATT GGG ATA
teg58-argG 8bp deletion F	ATA GTT GTT TAT GTT TTG AAC AAG CGA
Teg58-*argG* 8 bp deletion R	TTT AAG TAA TTA TTT ATA TTA TTT ATC CCA
Teg58-*argH* 9 bp scramb F	TTA AAA ATA CAA TGA GAT ATA GTG GCA ACC GTG TTA A
Teg58 -*argH* 9bp scramble R	GAA ACT TAA TTG ATA GCA ATA GGT TTT CAC AAT AGC CAC CTC GCT
argGH 2073 F	AAG GGA ACTC GAG AAT GAG GTG AAT TTT ATG AAA GAG AA
argGH 2073 R	AGA GAAG GAA TTC GAG CAT TAA AAT ACC CAC AAT GTA
argGH promoter F	AGA GAAG GAA TTC ATT TAA TAT CTT AAT TTT ACT AA
a*rgGH* promoter R	AGGA GAA TCTAGATGT AGT TAT CTG AAT ATT TCG TAT GAA
argG 9nt deletion F	CCT GTA CGT GAG TGG GCA TGG A
argG 9nt deletion R	TTT CAA TGA TGG GTT TAA TGC TTT AA
argH 9nt scramble F	TCA CTA GTG TAA TTG TAG ACA TCG CTT CCA ATA
argH 9nt scramble R	GGCT CTT AAT TAA TGC GAT GAT ATC TTG CA
argG orf probe F	AGC ATA TTC AGG AGG ACT AGA TAC AA
argG orf probe R	ATT GCT CAT TGC TAT AGC CTC CTA GCA
argH orf probe F	AGC AAT AAA GCT TGG GGC GGT AGA
argH orf probe R	ATT GTG ATA GTA ATT GTT TAG CAA CAT CA
arg -1 T7 F	AGATGTAATACGACTCACTATAGGATGAAAGAGAAAATTGTTTTAGCA
arg-1 T7 R	GAA CGC ATC CTC TGG TGG CGC
arg-2 T7 F	AGATGTAATACGACTCACTAAGG GGTATGCA CATGGTTGTACTGGTAAA
arg-2 T7 R	ATC TTT CGT TAA CGT AAT CGT TTC TAA
arg-3 T7 F	AGATGTAATACGACTCACTATAGGAACACCAGATACTGCTGATGAAAT CA
arg-3 T7 R	ACTCTTCAGGTTGTACTTCAAATCTAC
arg-4 T7 F	AGATGAATACGACTCACTATAGGATGAGCAATAAAGCTGG GGCGGTA
arg-4 T7 R	TAA ACT ATC TTC AAA TCG TTG TTG GTC
arg-5 T7 F	AGATGTAATACGACTCACTATAGGGCCTAGTTATACTCATTTACAGCGTGCA
arg-5 T7 R	ACT AAA TAA TCT GCT AGT TCC GTT GCA
hla RT-F	AGT CAG CTC AGT AAC AAC AAC ACT A
hla RT-R	AGC ACC TTC TTC GCT ATA AAC TCT A
lukD RT F	AGG ATA AAA GTT ATG ACA AAG ATA CGT TAG
luk D RT R	CGC CCC AAT AAA ACT GTG AG
luk F RT F	TGC TCC TGA TAA TCA ATT GCC A
luk F RT R	ACG AAA GAA AGA CGC ATT TAT GGT
hlb RT-F	GTG ATA ATG ATG GTG AAA AAA ACA
hlb RT-R	AGT CTC CAG TTT GGA TAC AAA ACG GTC
hly III RT F	ATT GGT AAT GCG GCA TCT CAT
*hly*III RT R	AAC CTA TCC AAC CAC CTA CAA C

hlgA RT F	AAT ATC GGC GGA AAC TTC CAA TCA
hlgA RT R	TCA AAC TCG CTT TTA TCA CCT TTA
hlgC RT F	AGA GAT AGC TTC CAC CCA ACA
hlgC RT R	TTT ATA ATT TGC GCC TGC CCA GTA
hlgB RT F	CGT TCC AGA CAG TGA GTT ACC A
*hlgB*RT R	GTC CGT CTA AAT AAC TGT TGC CA
coa RT- F	AGC TGA AGA AAC AAC ACA ACC AG
coa RT R	AGC TTC TGT TGT TTC AGT TGC AAG A
HP(Sao01113) RT F	AAC AAT TCT ATT ACT ACT TTG TAT A
HP (Sao01113) RT R	ATG CTC TAA AAC GAC AAC CTC
spa RT F	ATT ACG CAA GTG TGC TGT ATT CTA
spa RT R	AGT GTT GAG GCG TTT CAG AAG TTG T
argG RT F	CAT CAT TGA AAG CAT TCG CAC
argG RT R	CAC ATT CAT TCG CTC TGC C
rpoB RT F	AACGTCGTCTATCAGCATTAGG
rpoB RT R	TTTGGTCCCTCAGGTGTTTC
arcA F	AAGGAAGGATCCAATTGTTTCTACAATAGTTAAAAAGCA
arcA R	AGAGAAGGATCCTATAACTAGTCCAATTAAAGA
arcA ko F	ATCTTTGAATTCATTTAGAGTATAGACAA
arcA ko R	AATTATAGAAACGAGGGTGAAAATAA
rocF F	AGAAGAGGATCCTATTAAGTTCTCTATTTAAATGTTGAA
rocF R	AGGAGAGGATCCATCAAATGTTACCGAAATATCACC
rocF ko F	AGCGTCCCCCTTTGCTCTATGTAA
rocF ko R	ATGATTTGTAGTGTATAAAGTATATTT
argF F	AGGAGAGGATCCAAACACTTGAATTATGACTATA
argF R	AGGAGAGGATCCTTCAATTCAATTACTTTTTAATCACAGTA
argF ko F	AATAACAACTCCTTTTATTTTAGTTACA
argF ko R	ATATAAGGAAGTGAATATGATGGCGAA
ureA F	AGAGAAGGATCCGTATCTACAAATTTAACCTGT
ureA R	AGGAGAGGATCCGCGATATCTTCAGGAATAGCCGCATTTA
ureA ko F	AATTTCATATTAGATACAATTAC AAAA
ureA ko R	AAGGAGGAAAAAGATCATGAGCTTTAA
arcA RT F	TCC ACA AGC CTC AAT AGG AC
arcA RT R	AAT GGG CAA TCT CGA TCT AAC
arcB RT F	TGC GTT TAC AGT TGC GTC
arcB RT R	TGC CAA TCG TCT GTT AAT CC
argF RT F	ACA CTC TCC GAG GAT TTA AAA C
argF RT R	ATC ATA CAT TCC ACC AAG CAC
rocF RT	GTA ATC AAG CCA GAA AAC ATC G
rocF RT R	ACC ACC CAA AAC TCT AGT ACC
arcD RT F	AAA GGA GGC AAC ACA TTA CC
arcD RT R	CGA TAC CGA TAA ACA CCC AAA C
ureA RT F	TTA CAC AAC GAG AGC AAG AC
ureA RT R	TCG CTG ATT AAA GCT AAT GCC
nos RT F	ATG GTG CTA AAA TGG CTT GG
nos RT R	GGT CCA TCC TTT GGA GCA TA

References

1. Wright, P. R. *et al*. CopraRNA and IntaRNA: predicting small RNA targets, networks and interaction domains. *Nucleic Acids Res.* **42**, W119-W123 (2014).
2. Fey, P. D. *et al.* A genetic resource for rapid and comprehensive phenotype screening of nonessential *S. aureus* genes. *mBio* **4,** e00537-12 (2013).
3. Horsburgh, M. J. *et al.* σ^B modulates virulence determinants expression and stress resistance: characterization of a functional *rsbU* strain of *Staphylococcus aureus* 8325-4. *J. Bacteriol.* **184**, 5457–5467 (2002).
4. Charlotte, O. *et al.* Expanding the *Staphylococcus aureus* SarA regulon to small RNAs. *mSystems* ***6,*** e00713-21 (2021).
5. Ballal, A. & Manna, A. C. Control of thioredoxin reductase (*trxB*) transcription by SarA in *Staphylococcus aureus*. *J. Bacteriol*. **192**, 336-345 (2010).
6. Monk, I. R. *et al.* Complete bypass of restriction systems for major *Staphylococcus aureus* lineages. *mBio* **6**, e00308-15. doi:10.1128/mBio.00308-15 (2015).
7. Arnaud, M., Chastanet, A. & Debarbouille, M. New vector for efficient allelic replacement in naturally nontransformable, low-GC content Gram-positive bacteria. *Appl. Environ. Microbiol.* **70**, 6887-6891 (2004).
8. Gaskill, M. E. & Khan, S. A. Regulation of the enterotoxin B gene in *Staphylococcus aureus*. *J. Biol. Chem*. **263**, 6276–6280 (1988).
9. Cheung, A. L., Nast, C. C. & Bayer, A. S. Selective activation of *sar* promoters with the use of green fluorescent protein transcriptional fusions as the detection system in the rabbit endocarditis model. *Infect. Immun*. **66**, 5988–5993 (1998).
10. Bateman, B. T. *et al.* Evaluation of a tetracycline-inducible promoter in *Staphylococcus aureus in vitro* and *in vivo* and its application in demonstrating the role of *sigB* in microcolony formation. *Infect. Immun*. **69**, 7851–7857 (2001).
