# Supplementary material for: Teg58, a small regulatory RNA, is involved in regulating arginine biosynthesis and biofilm formation in Staphylococcus aureus
Source: Sci Rep. 2022 Sep 2;12:14963. doi: 10.1038/s41598-022-18815-3 (PMC9440087; doi:10.1038/s41598-022-18815-3)
Supplement: Supplementary file 2 — Supplementary Information 2. [file 41598_2022_18815_MOESM2_ESM.pdf]

ORIGINAL IMAGES:

Teg58, a Small Regulatory RNA, is Involved in regulating Arginine Biosynthesis and Biofilm Formation in *Staphylococcus aureus*

Adhar C Manna[1]*, Stefano Leo[2], Sergey Girel[3,4], Víctor González-Ruiz[3,4], Serge Rudaz[3,4], Patrice Francois[2], and Ambrose L. Cheung[1]

[1] Department of Microbiology & Immunology, Geisel School of Medicine at Dartmouth, Hanover, New Hampshire 03755, USA

[2] Genomic Research Laboratory, Service of Infectious Diseases, Geneva University Hospitals and University Medical Center, Rue Michel-Servet 1 CH-1211 Geneva 4, Switzerland

[3]Institute of Pharmaceutical Sciences of Western Switzerland (ISPSO), [4]School of Pharmaceutical Sciences, University of Geneva, Geneva, Switzerland. University Medical Center, Rue Michel-Servet 1, 1211 Geneva 4, Switzerland

Running title: sRNA Teg58 involve in biofilm formation

*Address correspondence to Adhar C. Manna, Adhar.C.Manna@Dartmouth.edu.

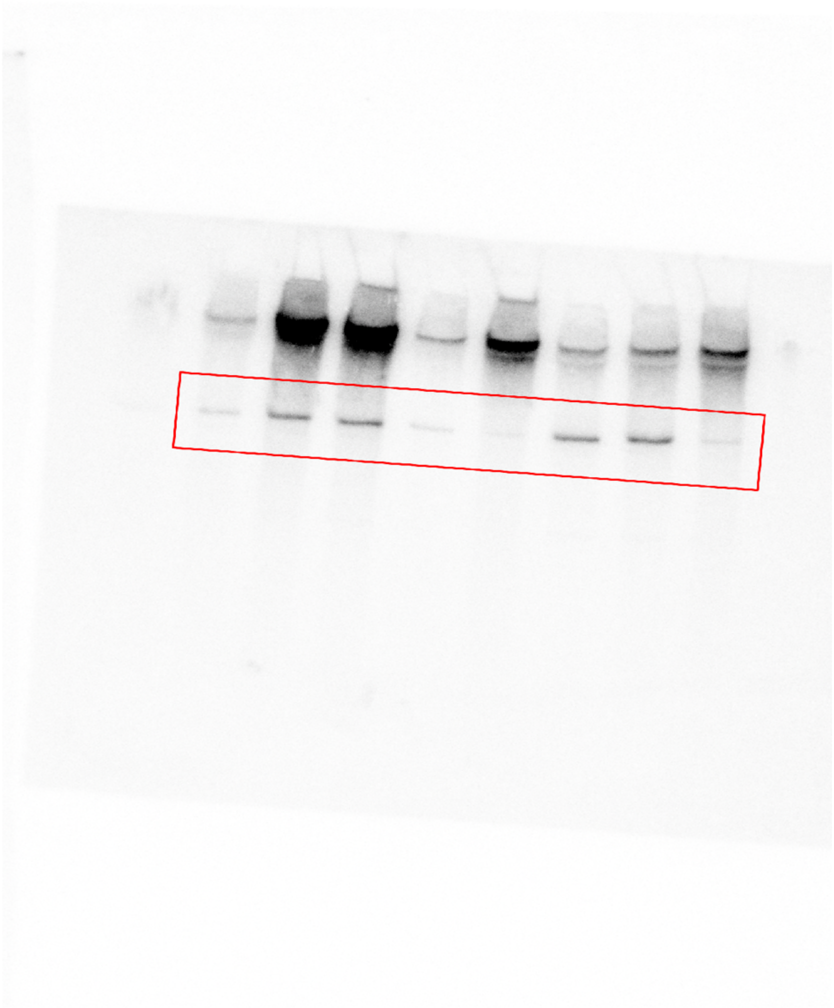

Figure X1A. Fig.2 A. Panel 1/Top. Original PhosphoImager scan image for Teg58. Red box region was cropped for the manuscript main figure 2A panel top.

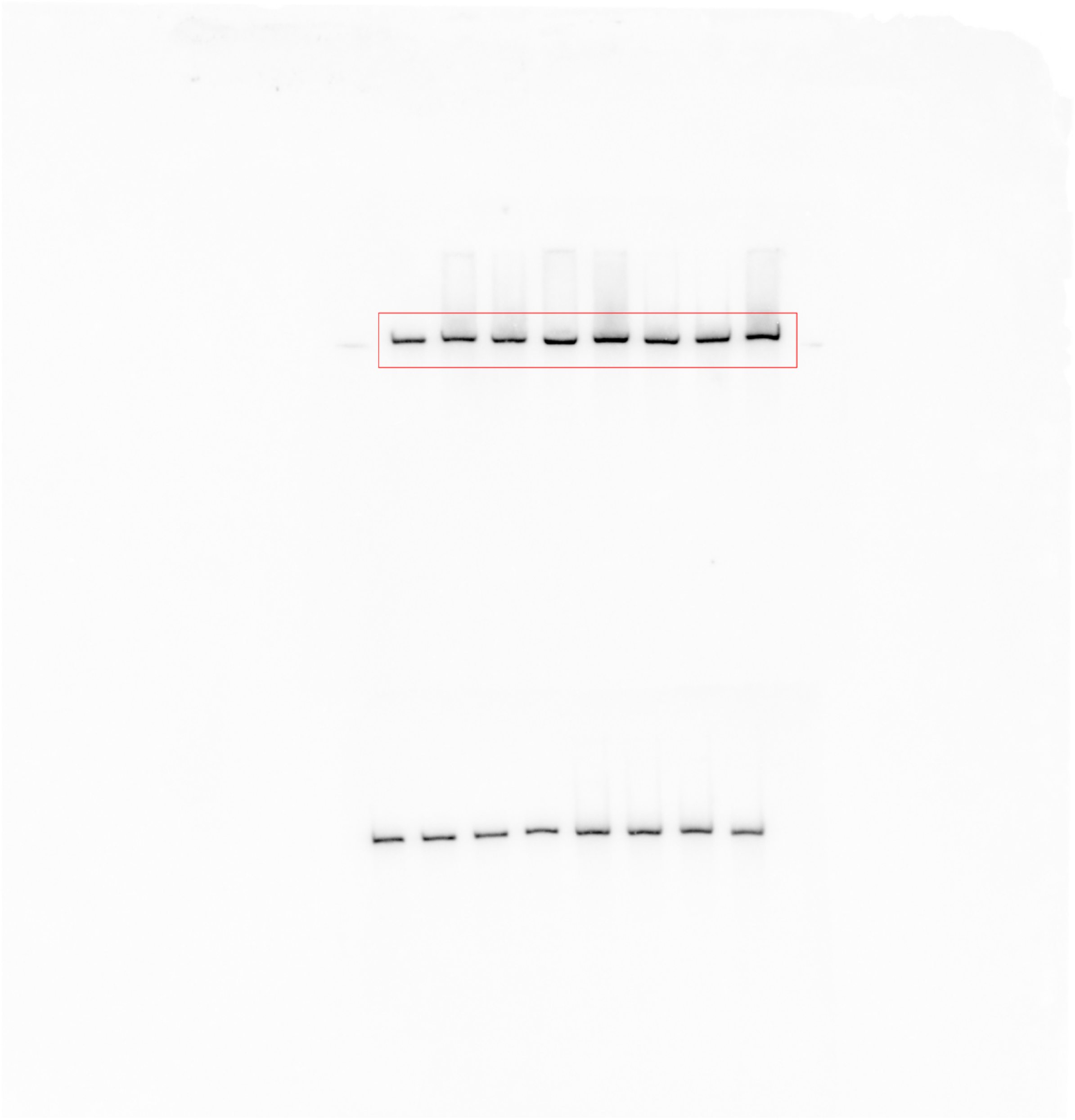

Figure X1B. Fig.2 A. Panel 2/ bottom. Original PhosphoImager scanned images for Tm. Red box region was cropped for the manuscript main figure 2A panel bottom.

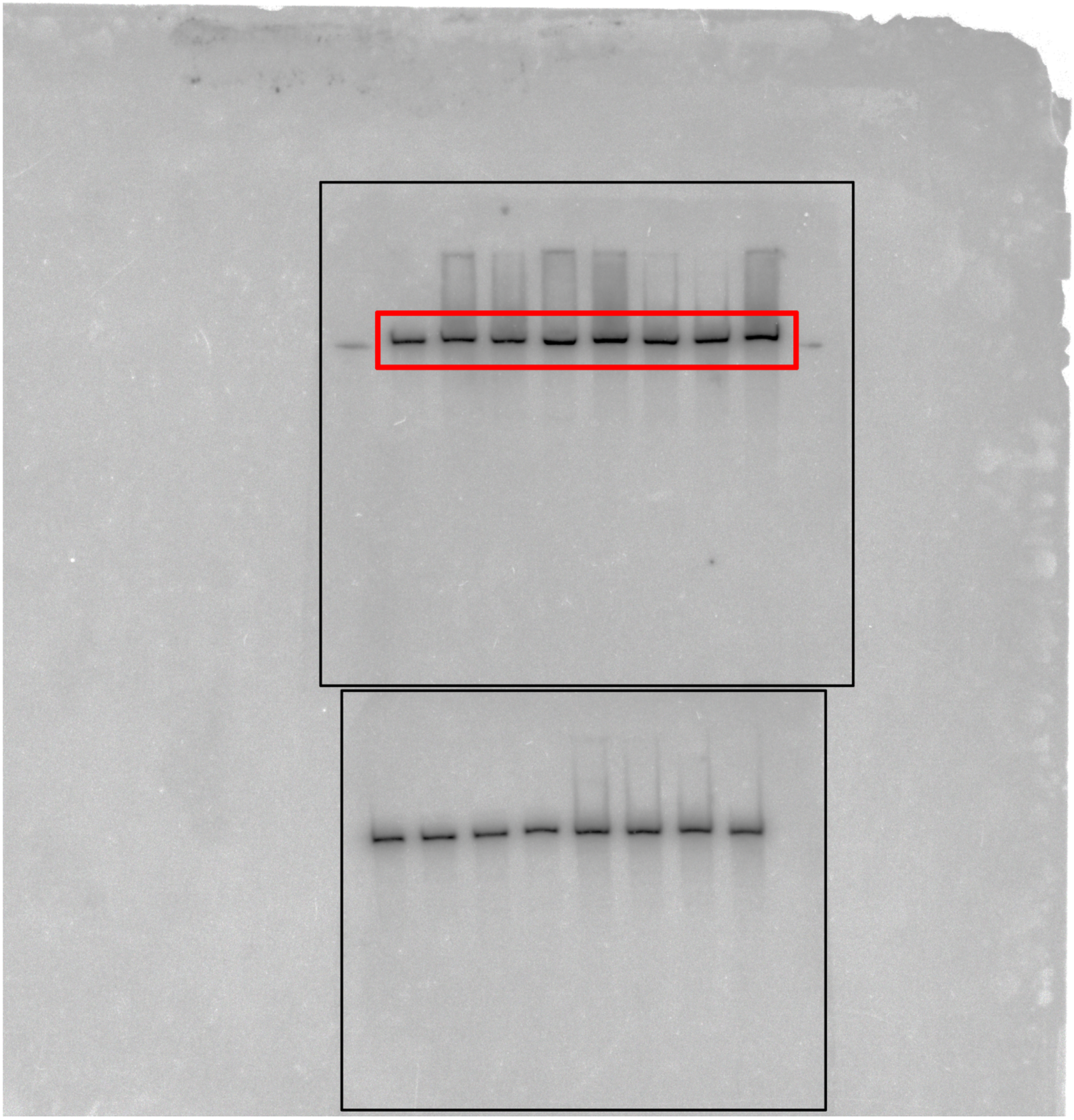

Different Exposure

Figure X1B1. Fig.2 A. Panel 2/ bottom. Original PhosphoImager scanned images for Tm. Red box region was cropped for the manuscript main figure 2A panel bottom.

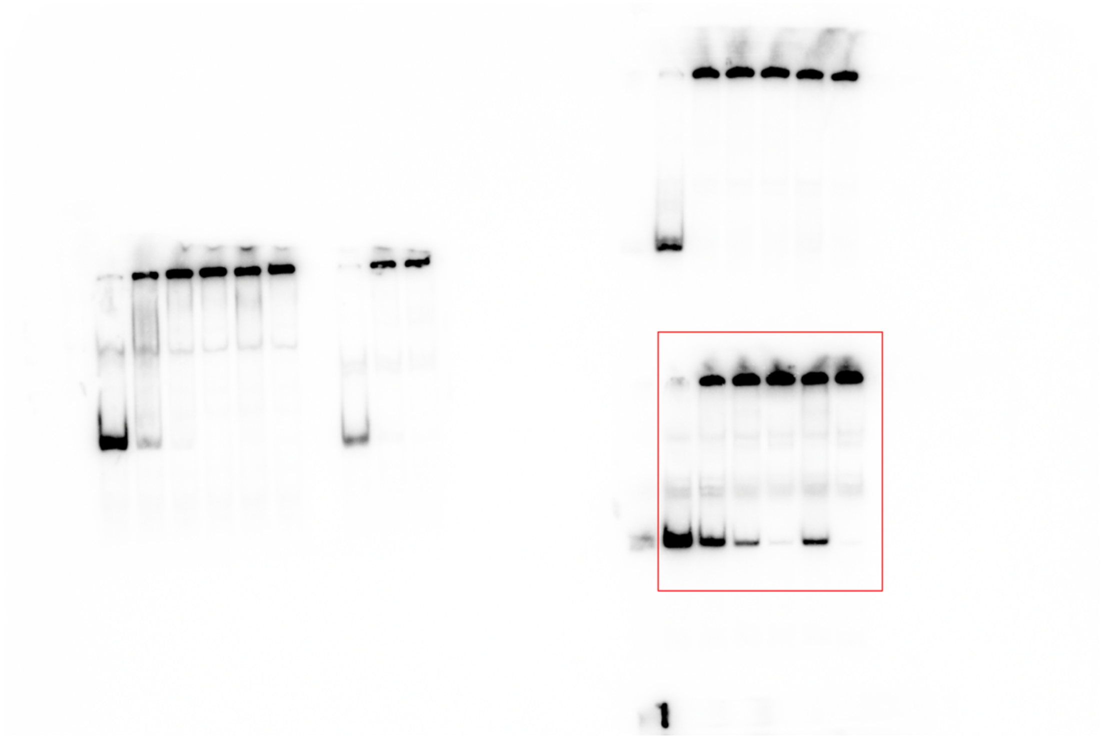

Figure X1C. Fig.2 C. Original PhosphoImager scanned images for gel shift experiment. Red box region was cropped for the manuscript main Figure 2C.

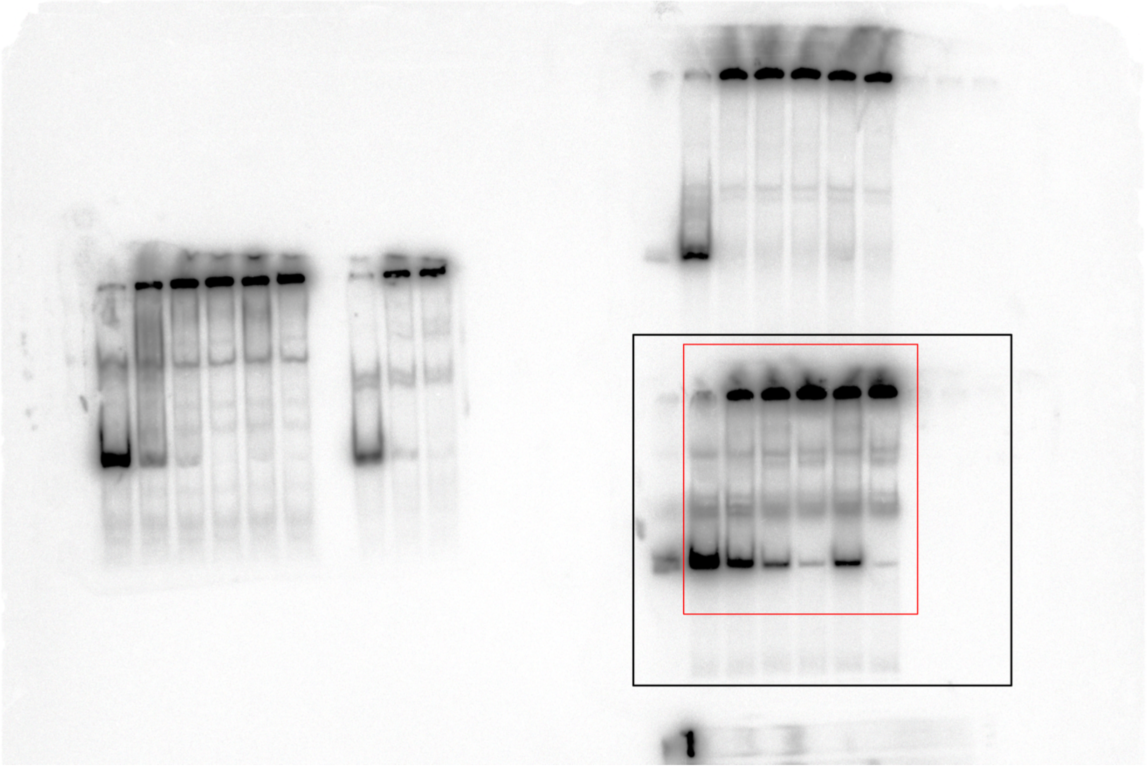

Different Exposure for **Figure X1C. Fig.2 C. Original PhosphoImager scanned images for gel shift experiment.**

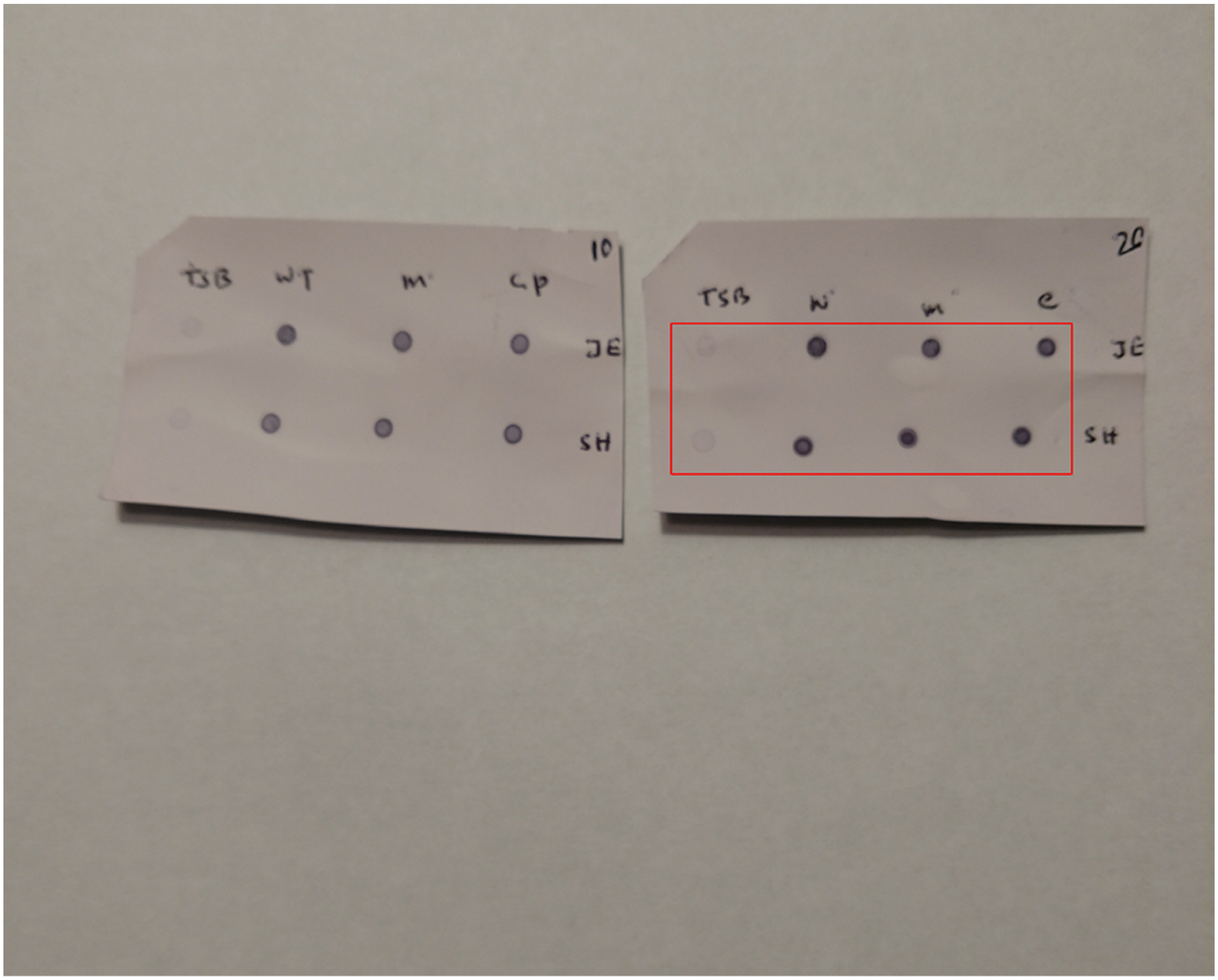

Figure X2. Fig.3C. Original scanned images for Dot blot with anti-PIA antibodies. Red box region was cropped for the manuscript main Figure 3C. Other blot with half amount of the cell-wall associated PIA loaded.

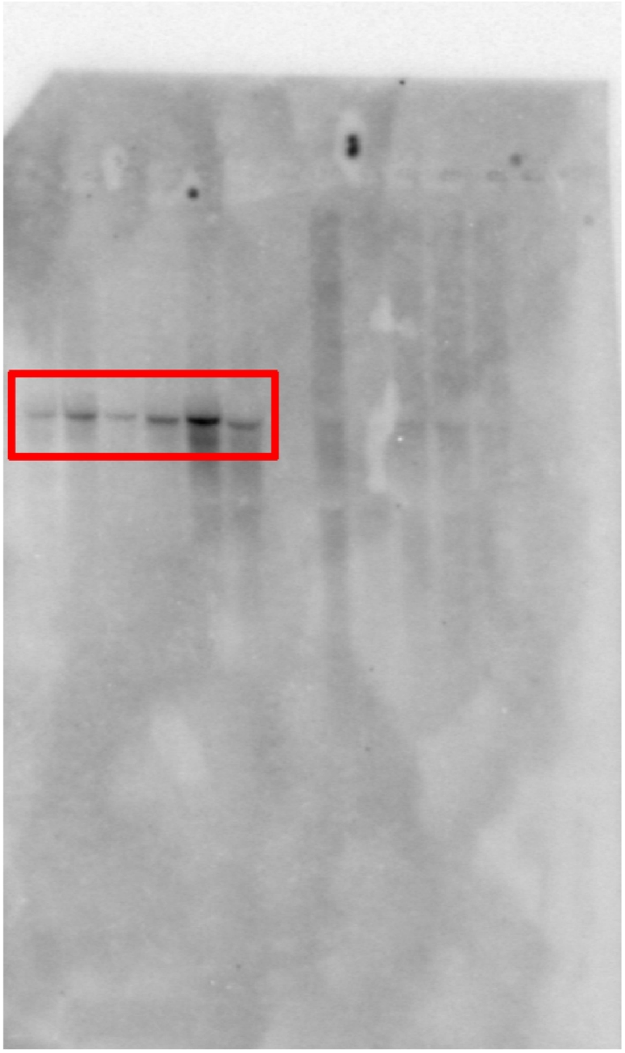

Figure X3A. Fig. 4 A. Panel 1 from the top. Original PhosphoImager scanned image for *argG*. Red box region was cropped for the manuscript main figure 4A panel 1 from the top.

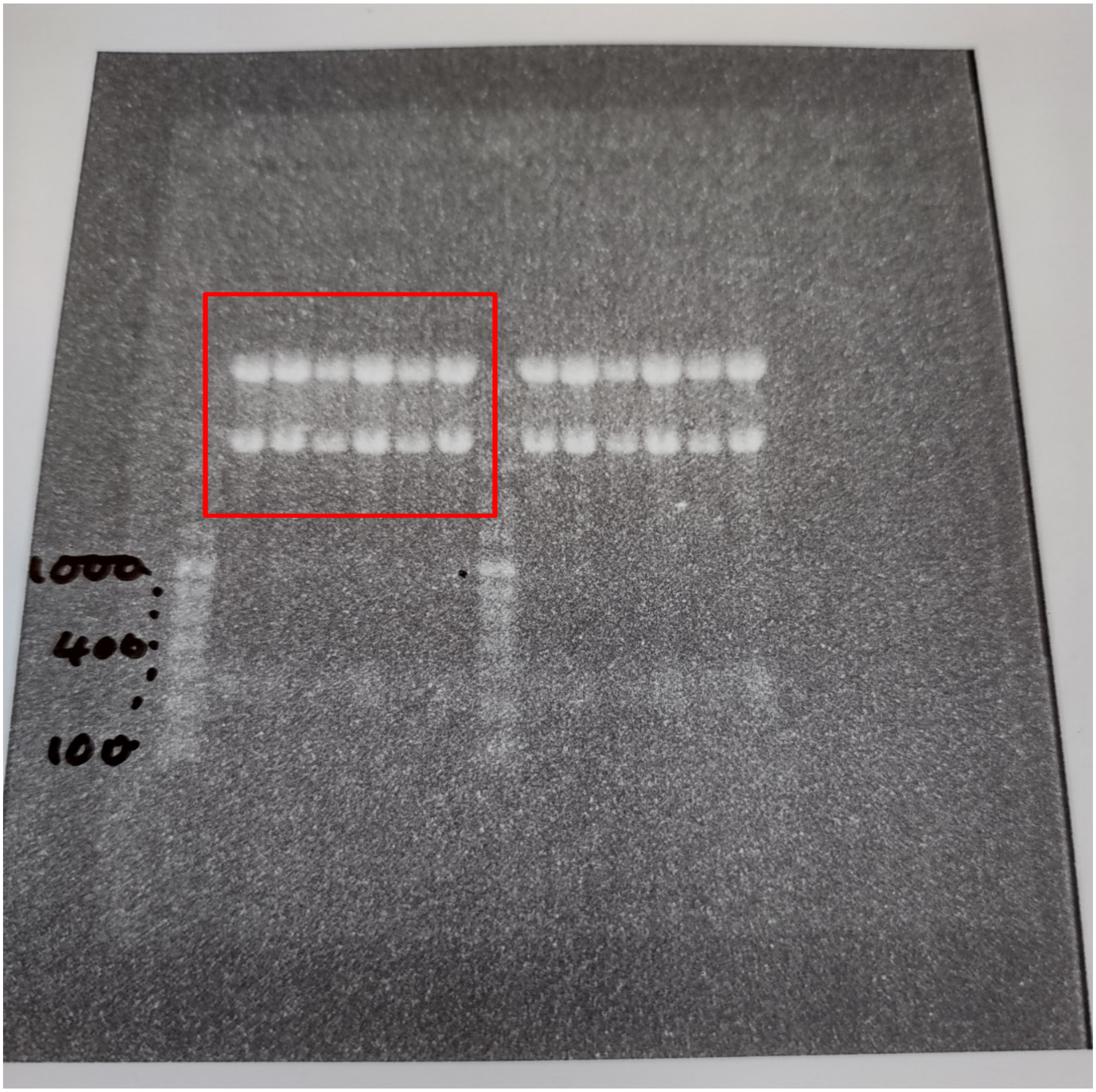

Figure X3B. Fig. 4 A. Panel 2 from the top. Original ethidium bromide strained Agarose gel scanned image for *argG*. Red box region was cropped for the manuscript main figure 4A panel 2 from the top.

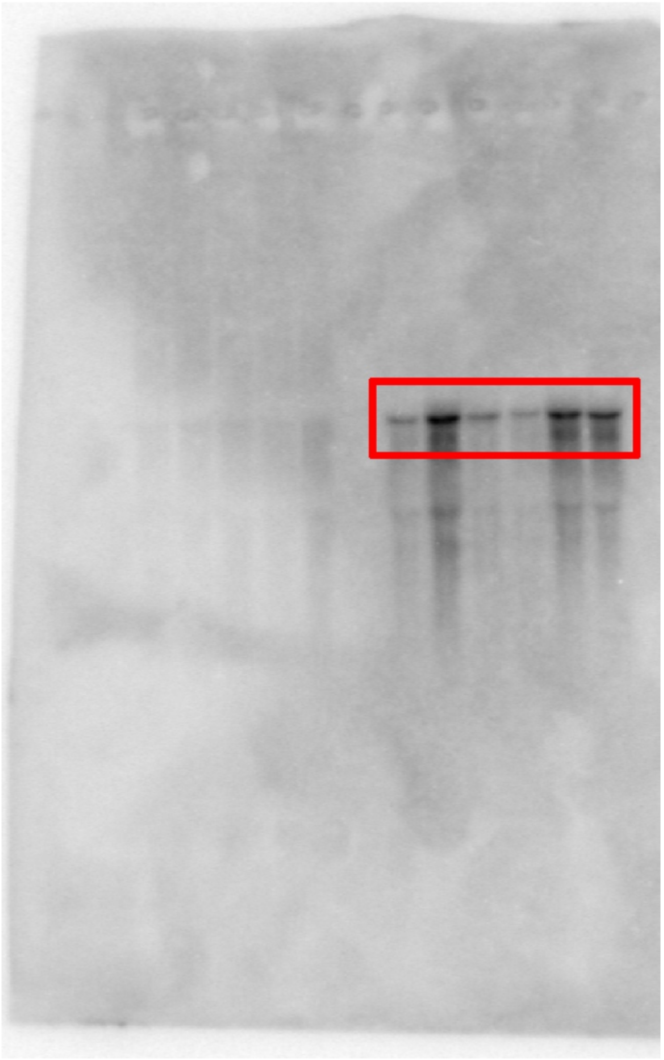

Figure X3C. Fig. 4 A. Panel 3 from the top. Original PhosphoImager scanned image for *argH*. Red box region was cropped for the manuscript main figure 4A panel 3 from the top.

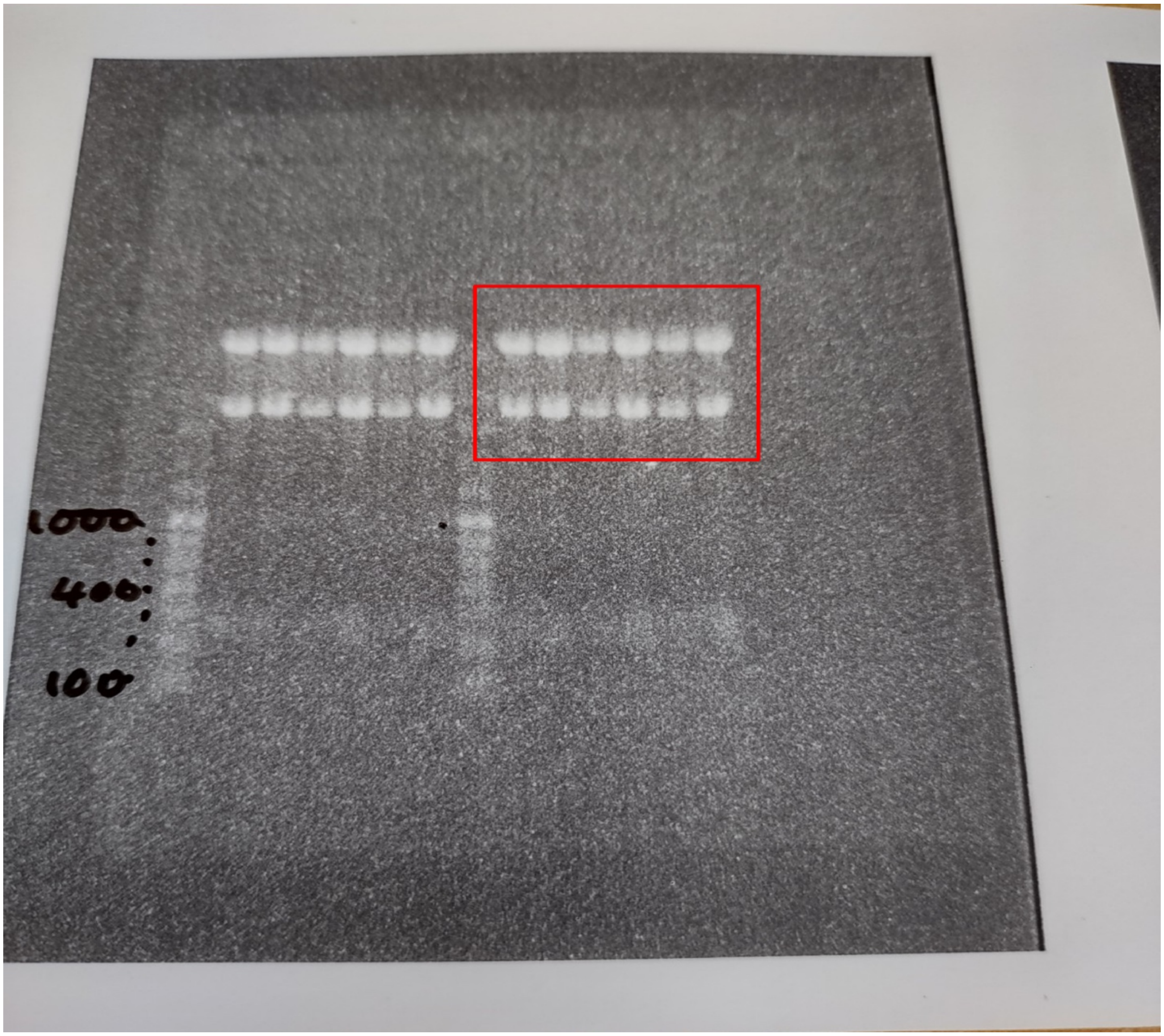

Figure X3D. Fig. 4 A. Panel 4 from the top. Ethidium bromide strained Agarose gel scanned image for *argH*. Red box region was cropped for the manuscript main Figure 4A panel 4 from the top. This scanned image is the same as Figure X3B (due to non-availability of image). All RNA-agarose gels (Fig.4A) were loaded equal amount of total cellular RNAs and run simultaneously under the same conditions single power supply and photographed. Visually corresponding number lanes are having similar amount of total RNAs.

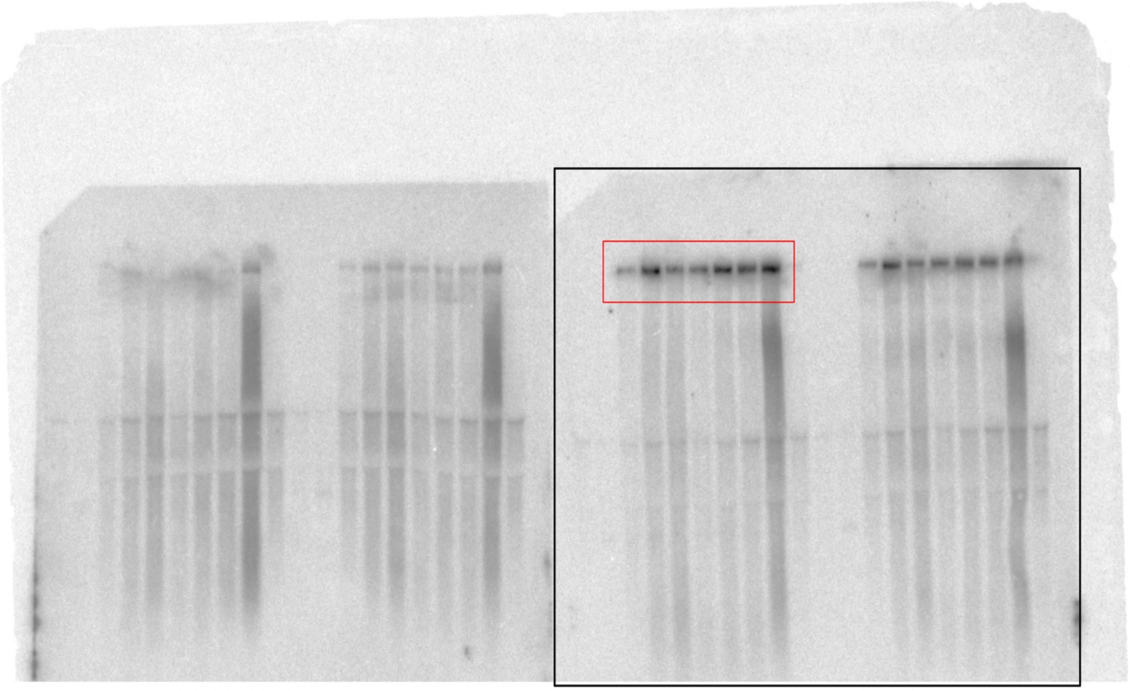

Figure X4A. Fig. 5D. Top Panel. Original PhosphoImager scanned images for Northern blot with *argG.* Red box region was cropped for the manuscript main figure 5D top panel.

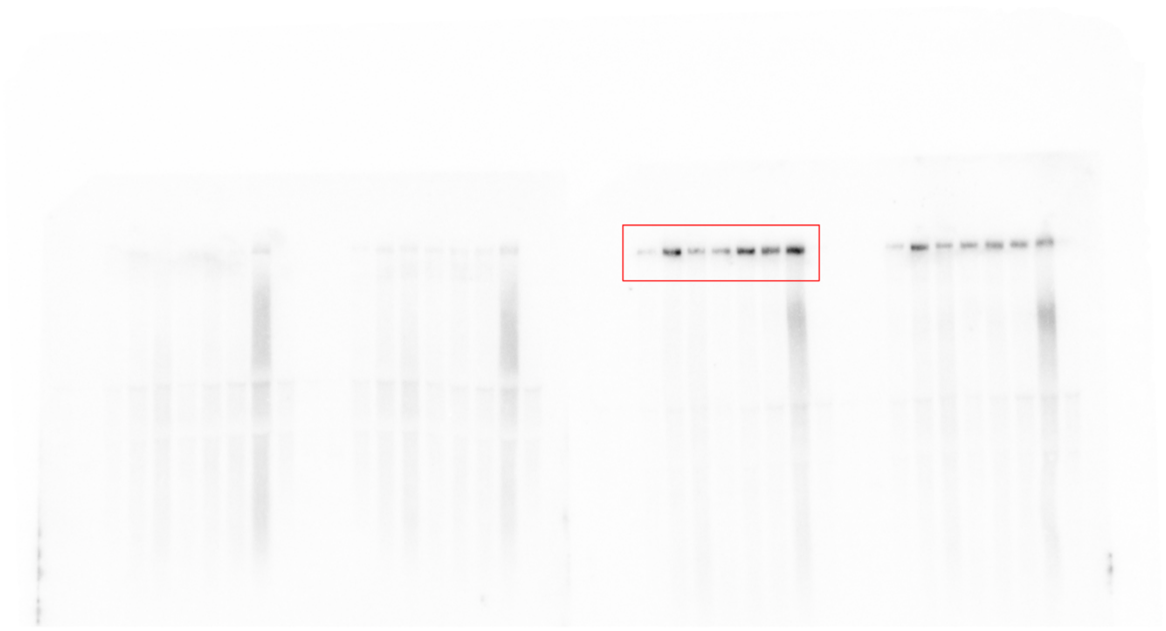

Different exposure (low contrast) for Figure X4A.

Figure X4A-1. Fig. 5D. Top Panel. Original PhosphoImager scanned images for Northern blot with *argG.* Red box region was cropped for the manuscript main figure 5D.

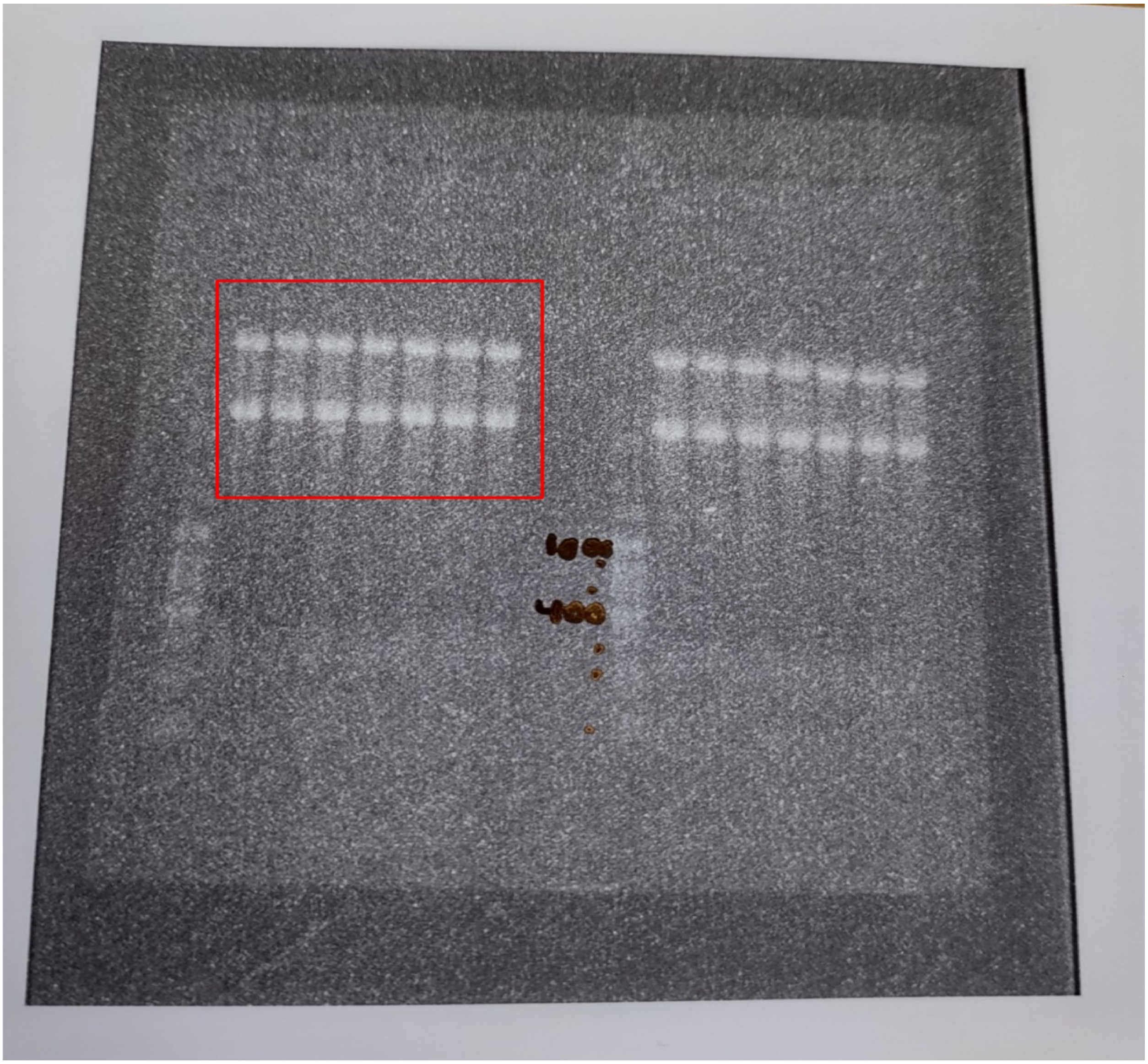

Figure X4B. Fig. 5D. panel 2. Original ethidium bromide strained Agarose gel scanned image for Northern blot with *argG.* Red box region was cropped for the manuscript main figure 5D panel 2.

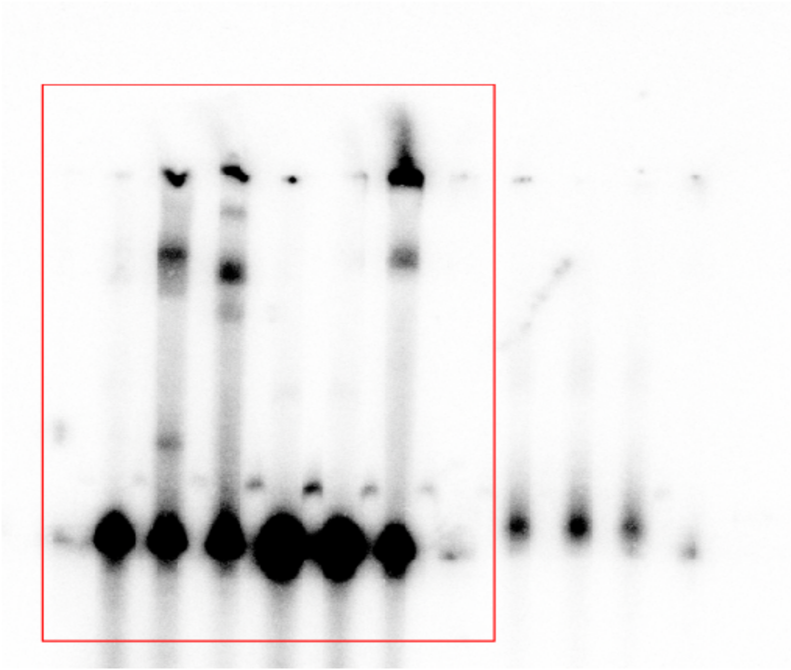

Different Exposure (low contrast) above of Figure X5A

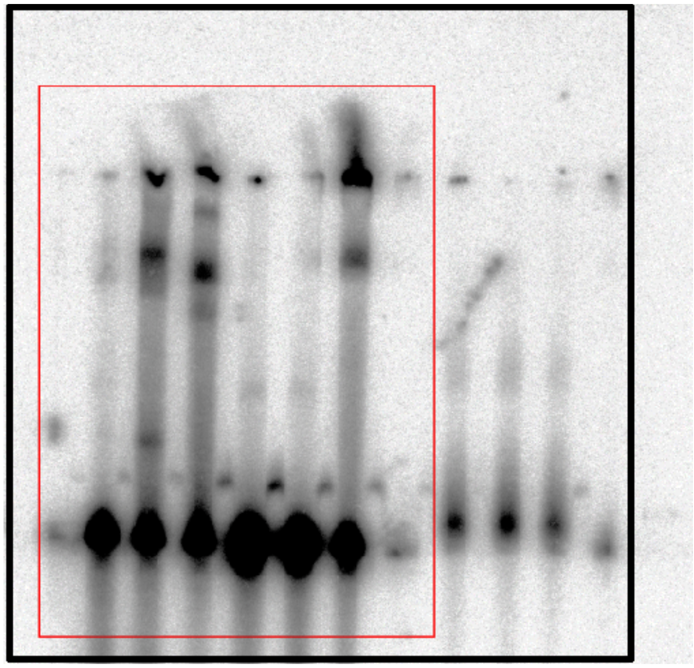

Figure X5A. Original PhosphoImager scanned image for mobility shift for RNA-RNA interaction. Red box region was cropped for the manuscript main Figure 6B.

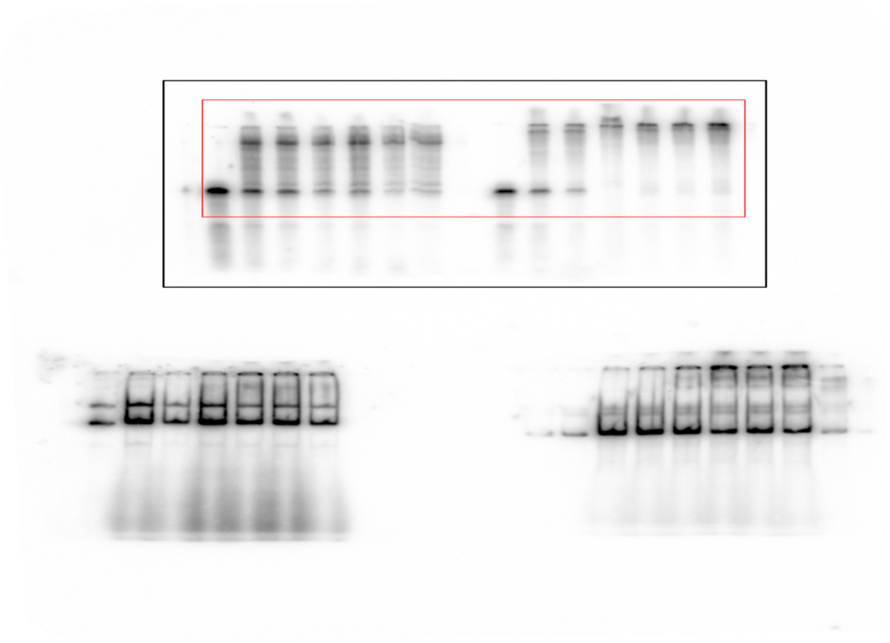

Figure X5B. Original PhosphoImager scanned images for mobility shift for RNA-RNA interaction - titration. Red box region was cropped for the manuscript main Figure 6C.

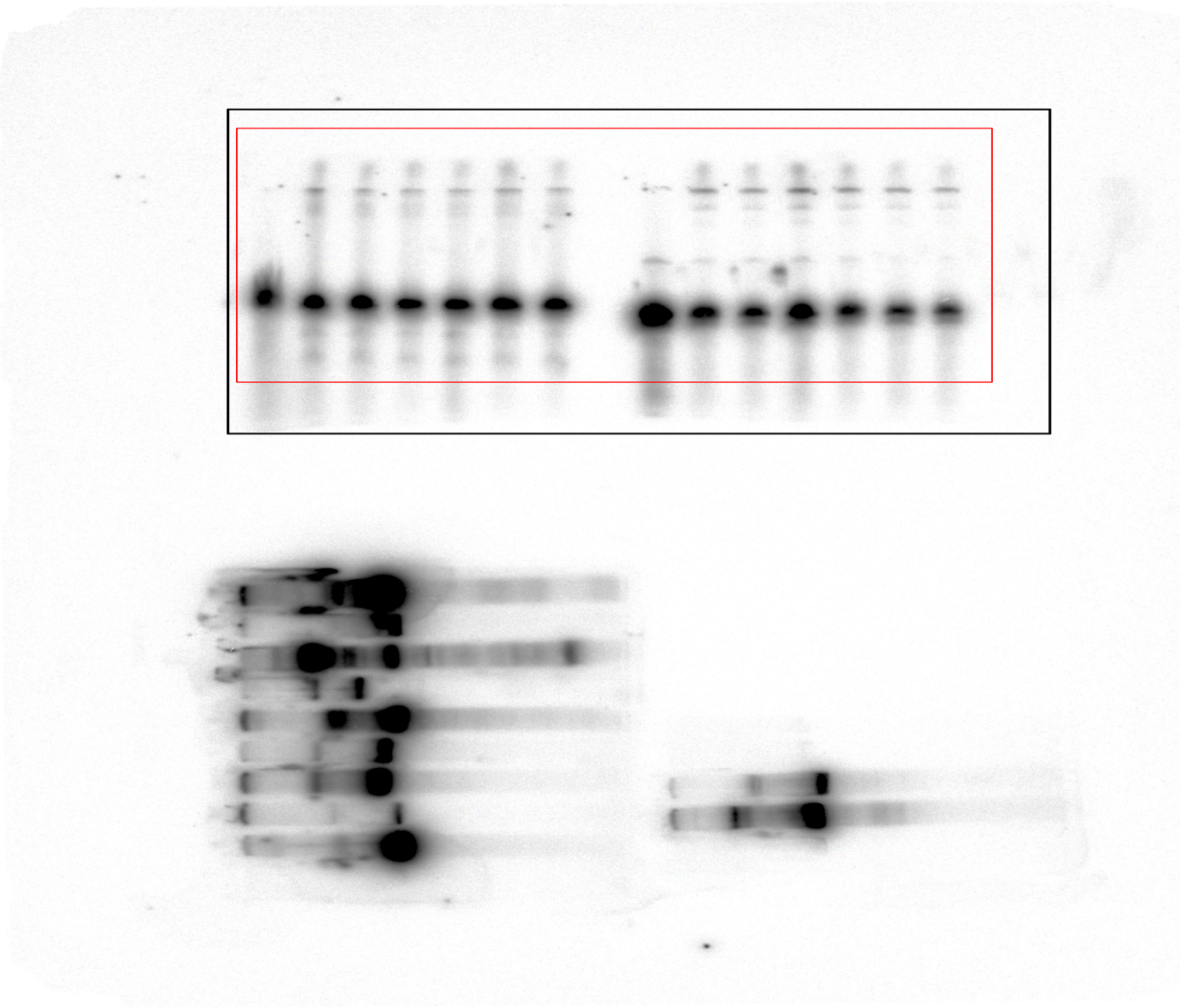

Figure X5 C. Original PhosphoImager scanned images for mobility shift for RNA-RNA interaction with mutated probes. Red box region was cropped for the manuscript main Figure 6D.

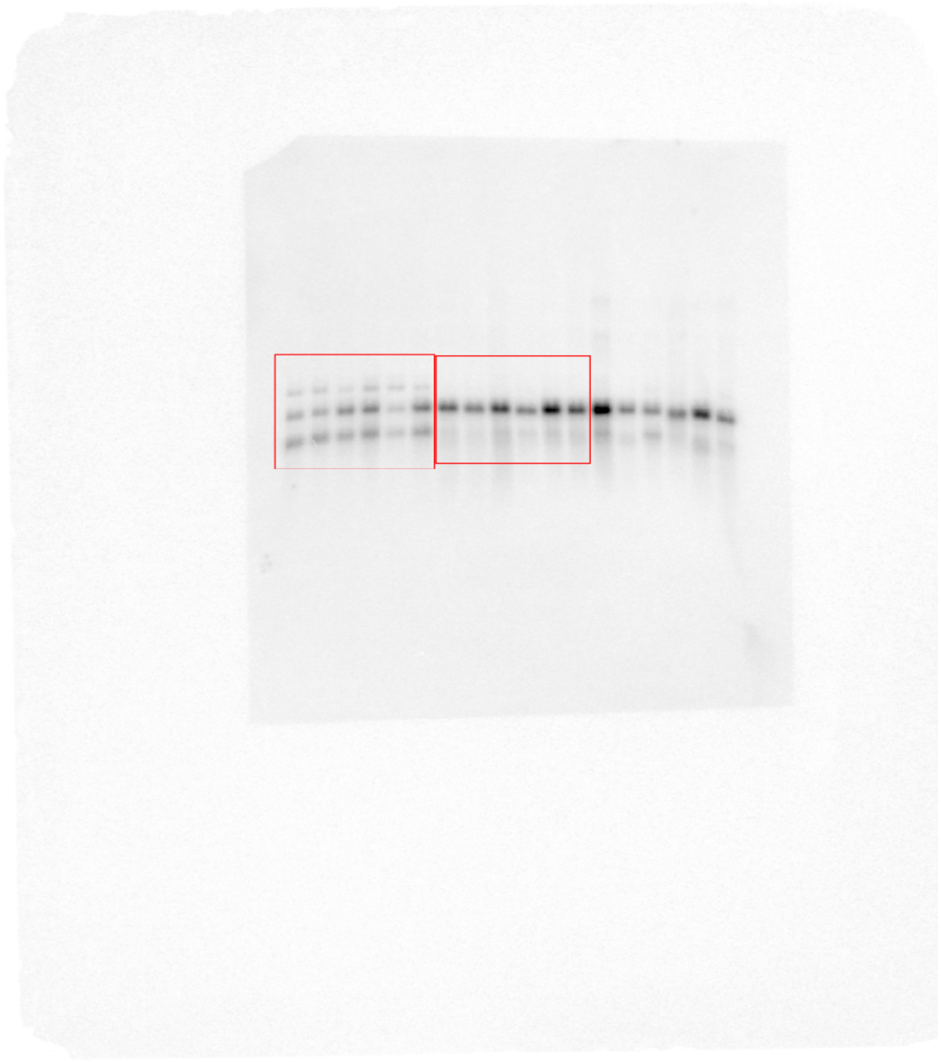

Figure X6A. Original PhosphoImager scanned image for Northern blot with *sarA* probe. Red box regions were cropped for the manuscript main Figure S2A.

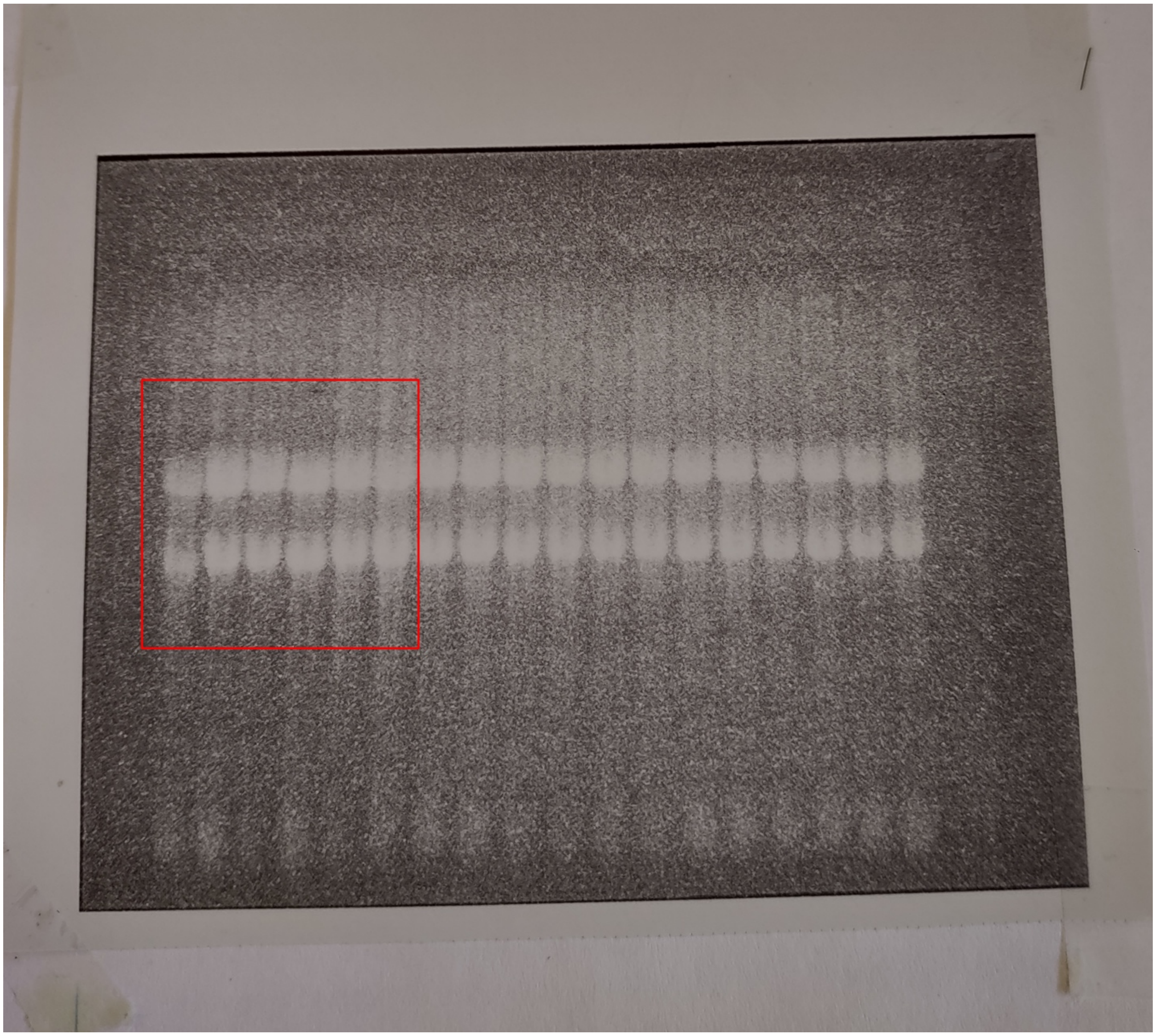

Figure X6B. Original ethidium bromide strained Agarose gel scanned image for Northern blot with *sarA* probe. Red box regions were cropped for the manuscript main Figure S2A.

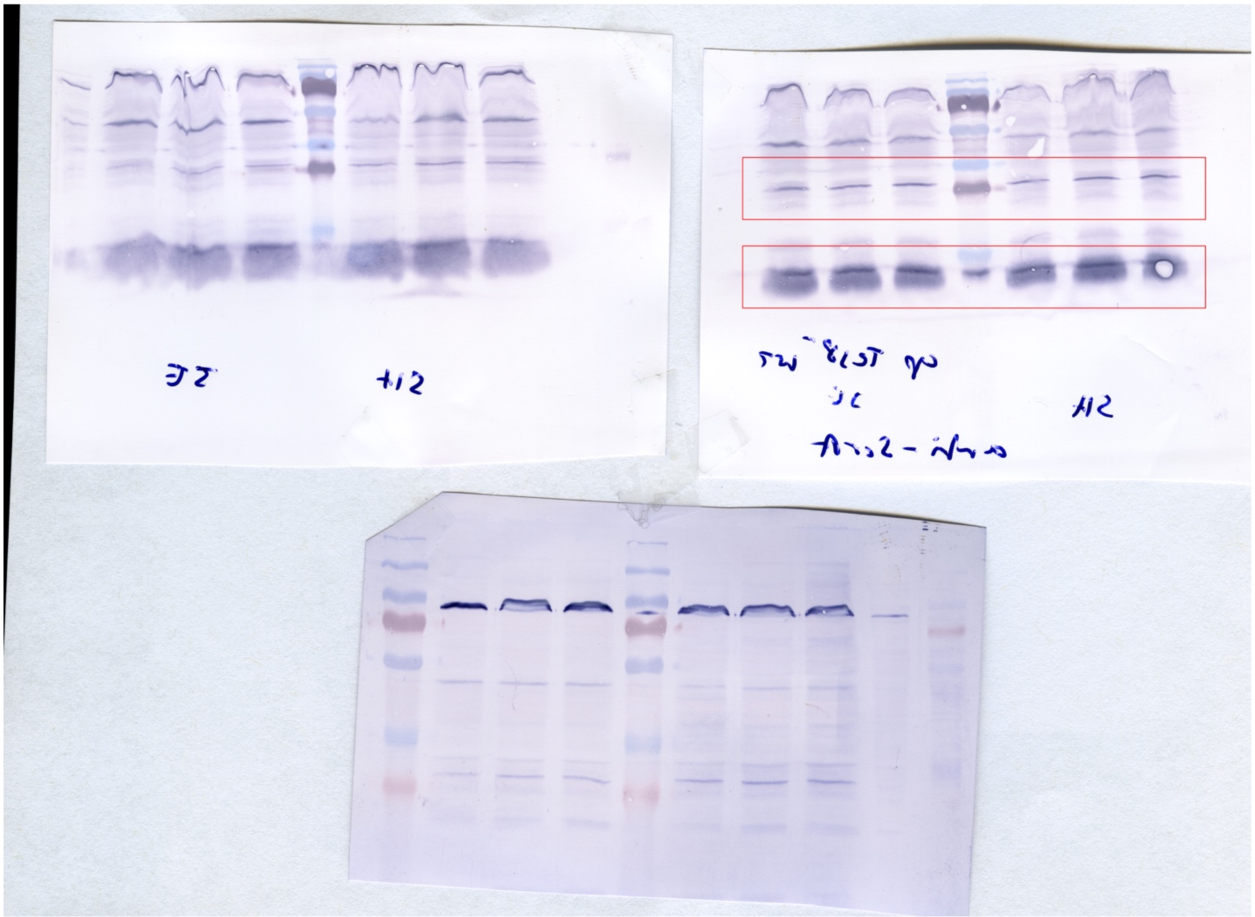

Figure X6 C. Original scanned images for Western blot with anti-SarA antibody. Red box regions were cropped for the manuscript main Figure S2B.

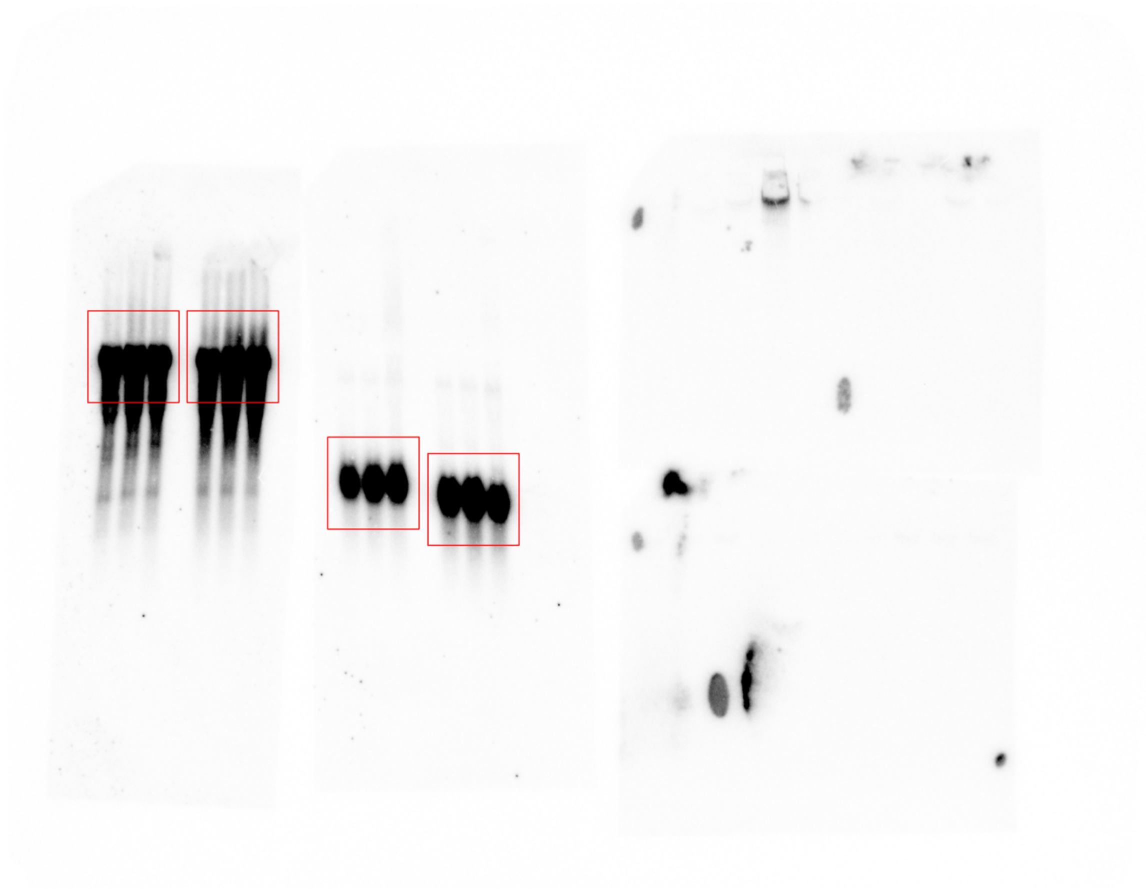

Figure X6 D. Original PhosphoImager scanned image for Northern blot with *agr* probes. Red box regions were cropped for the manuscript main Figure S2C.

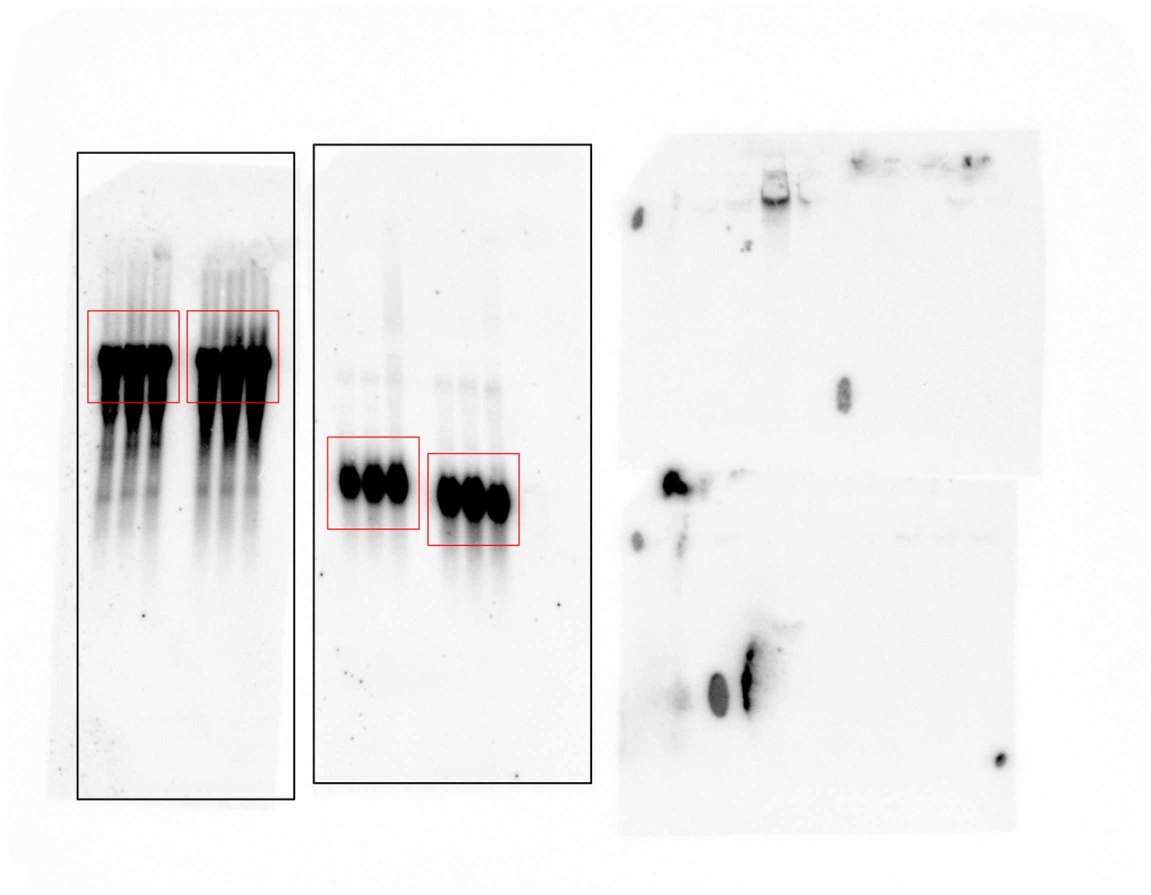

DIFFERENT EXPOSURE

Figure X6 D1. Original PhosphoImager scanned image for Northern blot with *agr* probes. Red box regions were cropped for the manuscript main Figure S2C.

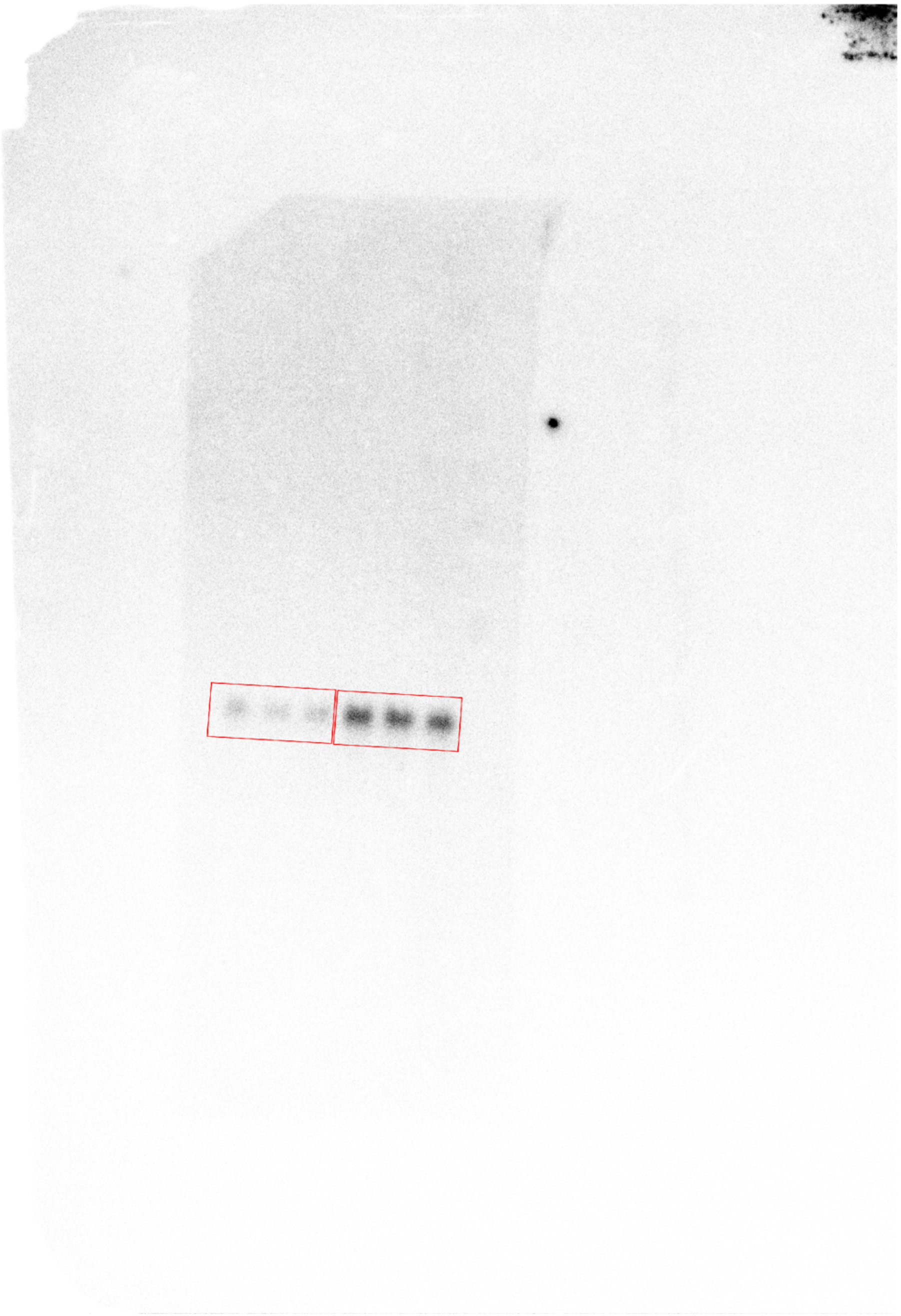

Figure X6 E. Original PhosphoImager scanned image for Northern blot with *psm-α* probe. Red box regions were cropped for the manuscript main Figure S2C.

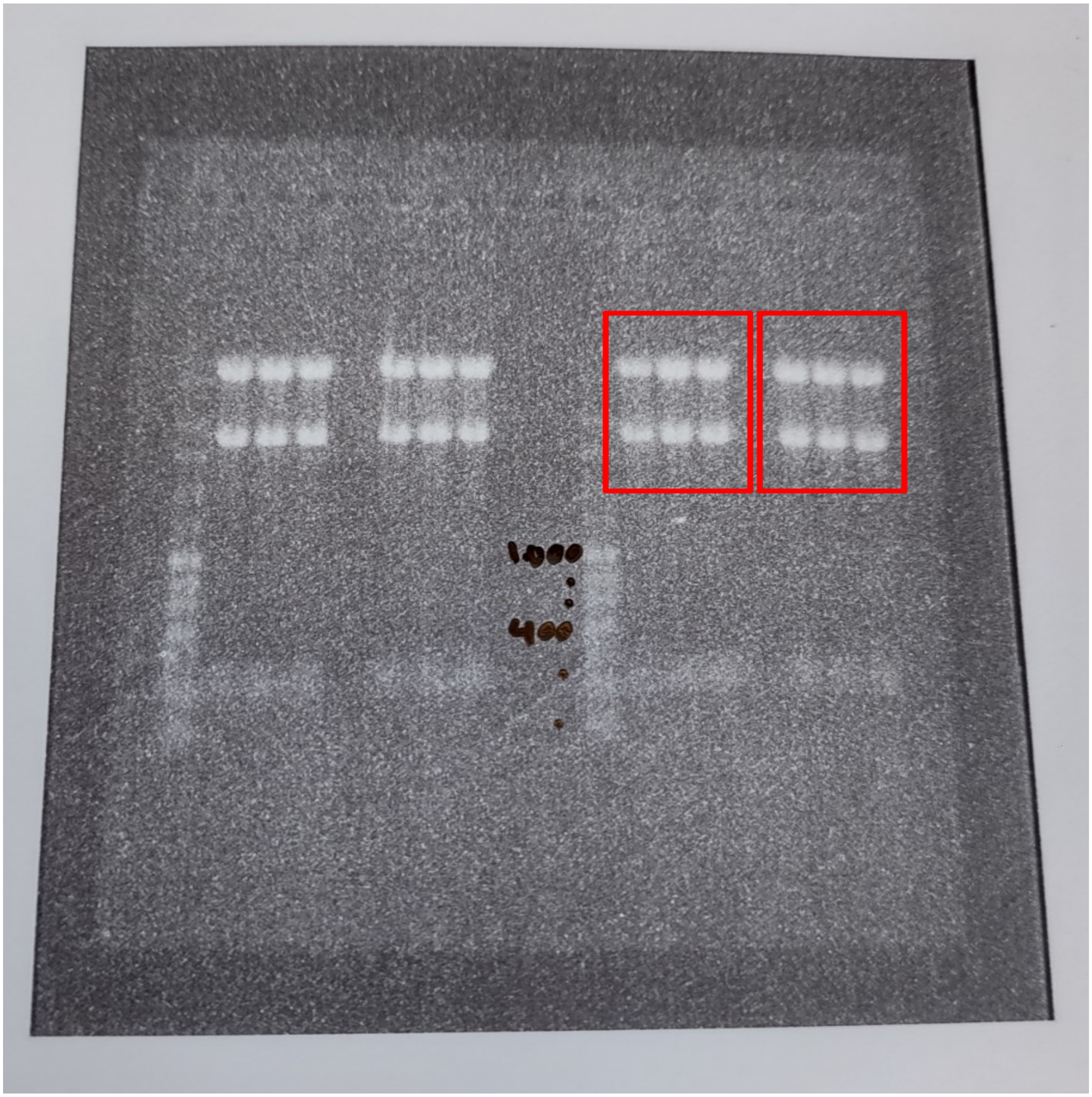

Figure X6 F. Original ethidium bromide strained Agarose gel scanned image for Northern blot with *agr* or *psm-α* probe. Red box regions were cropped for the manuscript main Figure S2C.

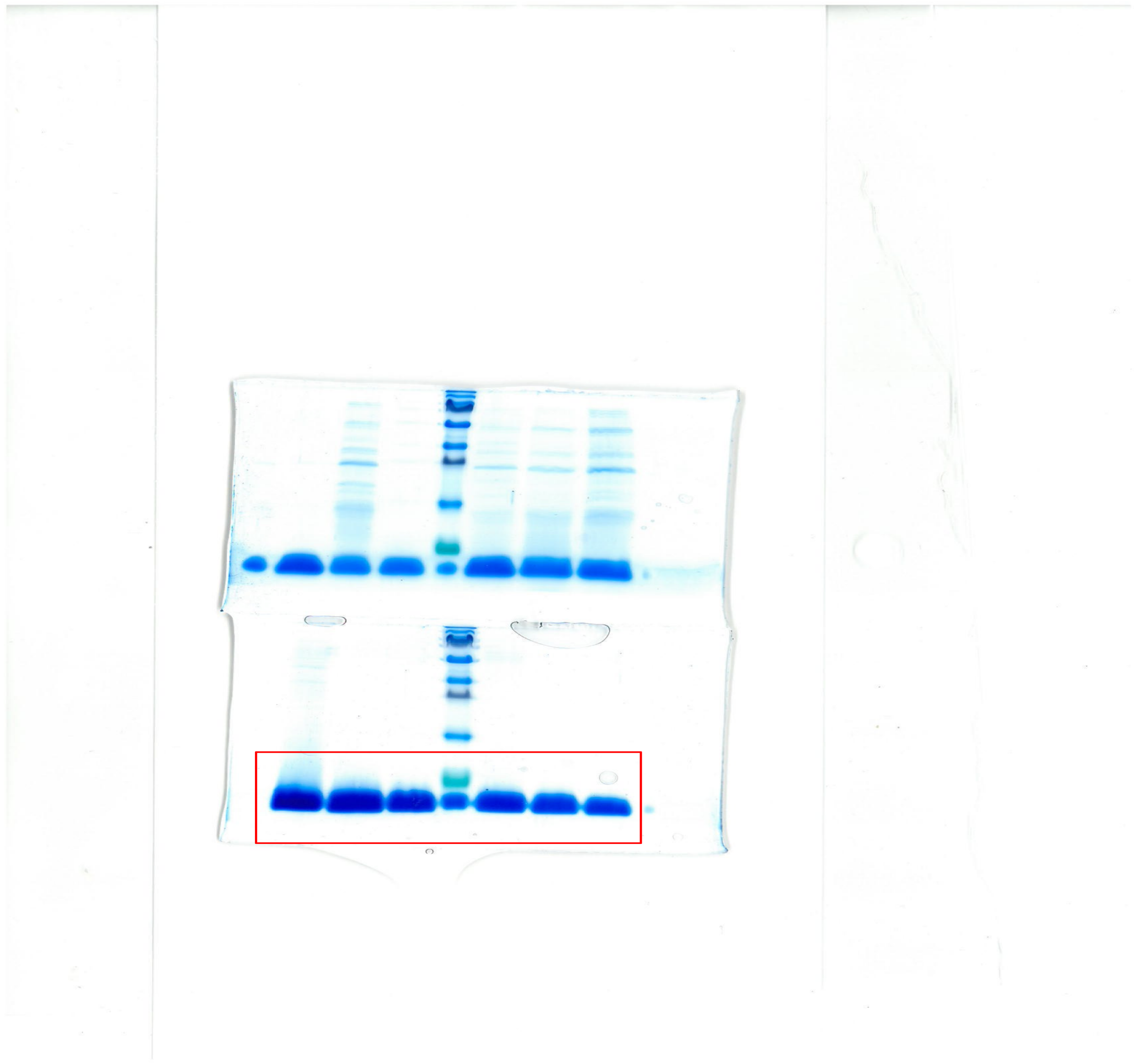

Figure X6 G. Original scanned images for Polyacrylamide-SDS gel. Red box region was cropped for the manuscript main Figure S2D.
